# Reduced blood-stage malaria growth and immune correlates in humans following RH5 vaccination

**DOI:** 10.1016/j.medj.2021.03.014

**Published:** 2021-06-11

**Authors:** Angela M. Minassian, Sarah E. Silk, Jordan R. Barrett, Carolyn M. Nielsen, Kazutoyo Miura, Ababacar Diouf, Carolin Loos, Jonathan K. Fallon, Ashlin R. Michell, Michael T. White, Nick J. Edwards, Ian D. Poulton, Celia H. Mitton, Ruth O. Payne, Michael Marks, Hector Maxwell-Scott, Antonio Querol-Rubiera, Karen Bisnauthsing, Rahul Batra, Tatiana Ogrina, Nathan J. Brendish, Yrene Themistocleous, Thomas A. Rawlinson, Katherine J. Ellis, Doris Quinkert, Megan Baker, Raquel Lopez Ramon, Fernando Ramos Lopez, Lea Barfod, Pedro M. Folegatti, Daniel Silman, Mehreen Datoo, Iona J. Taylor, Jing Jin, David Pulido, Alexander D. Douglas, Willem A. de Jongh, Robert Smith, Eleanor Berrie, Amy R. Noe, Carter L. Diggs, Lorraine A. Soisson, Rebecca Ashfield, Saul N. Faust, Anna L. Goodman, Alison M. Lawrie, Fay L. Nugent, Galit Alter, Carole A. Long, Simon J. Draper

**Affiliations:** 1 The Jenner Institute, University of Oxford, Oxford OX3 7DQ, UK; 2 Laboratory of Malaria and Vector Research, NIAID/NIH, Rockville, MD 20852, USA; 3 The Ragon Institute of MGH, MIT, and Harvard, Cambridge, MA 02139, USA; 4 Department of Biological Engineering, Massachusetts Institute of Technology, Cambridge, MA 02139, USA; 5 Department of Parasites and Insect Vectors, Institut Pasteur, 25-28 Rue du Dr Roux, 75015 Paris, France; 6 Centre for Clinical Infection and Diagnostics Research, King’s College London and Guy’s & St Thomas’ NHS Foundation Trust, Westminster Bridge Road, London SE1 7EH, UK; 7 NIHR Wellcome Trust Clinical Research Facility, University Hospital Southampton NHS Foundation Trust, Faculty of Medicine, University of Southampton, Southampton SO16 6YD, UK; 8 ExpreS^2^ion Biotechnologies, SCION-DTU Science Park, Agern Allé 1, Hørsholm 2970, Denmark; 9 Clinical BioManufacturing Facility, University of Oxford, Oxford OX3 7JT, UK; 10 Leidos Life Sciences, Fredrick, MD, USA; 11 USAID, 1300 Pennsylvania Ave. NW, Washington, DC 20004, USA

**Keywords:** vaccine, malaria, Plasmodium falciparum, RH5, blood-stage, CHMI, systems serology, clinical trial

## Abstract

**Background:**

Development of an effective vaccine against the pathogenic blood-stage infection of human malaria has proved challenging, and no candidate vaccine has affected blood-stage parasitemia following controlled human malaria infection (CHMI) with blood-stage *Plasmodium falciparum*.

**Methods:**

We undertook a phase I/IIa clinical trial in healthy adults in the United Kingdom of the RH5.1 recombinant protein vaccine, targeting the *P. falciparum* reticulocyte-binding protein homolog 5 (RH5), formulated in AS01_B_ adjuvant. We assessed safety, immunogenicity, and efficacy against blood-stage CHMI. Trial registered at ClinicalTrials.gov, NCT02927145.

**Findings:**

The RH5.1/AS01_B_ formulation was administered using a range of RH5.1 protein vaccine doses (2, 10, and 50 μg) and was found to be safe and well tolerated. A regimen using a delayed and fractional third dose, in contrast to three doses given at monthly intervals, led to significantly improved antibody response longevity over ∼2 years of follow-up. Following primary and secondary CHMI of vaccinees with blood-stage *P. falciparum*, a significant reduction in parasite growth rate was observed, defining a milestone for the blood-stage malaria vaccine field. We show that growth inhibition activity measured *in vitro* using purified immunoglobulin G (IgG) antibody strongly correlates with *in vivo* reduction of the parasite growth rate and also identify other antibody feature sets by systems serology, including the plasma anti-RH5 IgA1 response, that are associated with challenge outcome.

**Conclusions:**

Our data provide a new framework to guide rational design and delivery of next-generation vaccines to protect against malaria disease.

**Funding:**

This study was supported by USAID, UK MRC, Wellcome Trust, NIAID, and the NIHR Oxford-BRC.

## Highlights

The RH5.1/AS01_B_ vaccine is safe, well tolerated, and immunogenic in healthy adults
A delayed fractional third dose significantly improves antibody response longevity
*In vivo* blood-stage *P. falciparum* growth rate is significantly lower in vaccinees
*In vitro* IgG-mediated growth inhibition activity is associated with challenge outcome

## Context and significance

A highly effective vaccine against the human malaria parasite *Plasmodium falciparum* is urgently needed. One vaccine strategy aims to prevent parasite growth in the blood, protecting against clinical disease; however, this has proved exceptionally challenging. Here we show that a candidate vaccine (reticulocyte-binding protein homolog 5.1 [RH5.1]/AS01_B_) is safe in a phase I/IIa clinical trial and identify a vaccination regimen that improves the durability of the human antibody response, which is critical for long-term protection. Following experimental challenge of vaccinated adults with malaria, we observed that the vaccine could reduce parasite growth in the blood and identified immune responses that could predict how well the vaccine performs. These data will help guide the design of improved vaccines in the future.

## Graphical abstract

**Figure:**
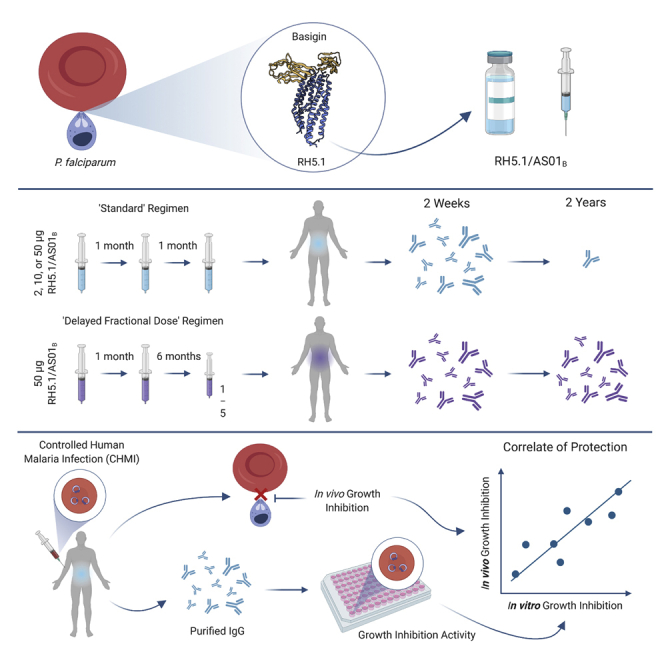

## Teaser

Minassian et al. report that the RH5.1/AS01_B_ vaccine against blood-stage *Plasmodium falciparum* malaria is safe and immunogenic in a phase I/IIa clinical trial. They demonstrate a significantly reduced blood-stage parasite growth rate in vaccinees following controlled human malaria infection and identify that *in vitro* antibody-mediated growth inhibition activity is associated with challenge outcome.

## Introduction

Despite major advances in malaria control, estimates in 2018 suggest that there were still 228 million clinical cases leading to 405,000 deaths.^1^ Consequently, there remains a pressing need for a highly effective and durable vaccine.^2^ Encouragingly, whole-parasite and subunit strategies targeting the invasive sporozoite and/or liver stage of *Plasmodium falciparum* have shown moderate levels of efficacy in field trials.^3^ However, these approaches continue to face various challenges related to durability or breadth of protection and immunopotency in target populations. They also necessitate sterilizing immunity to prevent the subsequent pathogenic blood stage of infection. An alternative and complementary approach is to vaccinate against the blood-stage merozoite leading to inhibition of erythrocyte invasion and, thus, control and/or clearance of blood-stage parasitemia, protecting against morbidity and mortality and reducing transmission.^3^ However, historical efforts to develop anti-merozoite vaccines have been thwarted by substantial levels of target antigen polymorphism,^4^ redundancy of erythrocyte invasion pathways,^5^ and a poor understanding of immune mechanisms that can provide *in vivo* protection in humans.

Nonetheless, substantial progress has been made in recent years following identification of an essential, highly conserved, antibody-susceptible, heterotrimeric protein complex used by *P. falciparum* merozoites to invade erythrocytes.^6^ Vaccine development efforts are most advanced for one component of the complex, called RH5.^3^ This vaccine target binds basigin on the erythrocyte surface,^7^ an indispensable receptor-ligand interaction linked to host erythrocyte tropism of *P. falciparum*^8^ and one the parasite has evolved to shield from the immune system. Indeed, RH5 appears to be poorly immunogenic in the context of natural malaria infection,^9^, ^10^, ^11^, ^12^ likely explaining its relatively high degree of sequence conservation. Conversely, vaccination of mice, rats, and rabbits with full-length RH5 (RH5_FL) induces high levels of functional antibodies that inhibit *in vitro* growth of all tested *P. falciparum* laboratory lines and isolates,^9^^,^^13^, ^14^, ^15^ notably with higher efficiency than other historical target antigens, such as merozoite surface protein 1 (MSP1) and apical membrane antigen 1 (AMA1).^13^^,^^16^ Additionally, significant *in vivo* protection has been demonstrated against a stringent blood-stage *P. falciparum* challenge in *Aotus* monkeys.^17^ These data provided momentum to advance RH5-based vaccines into clinical testing.

Expression of RH5_FL protein proved challenging for a number of years, and, consequently, the first vaccine regimen that progressed to phase Ia clinical testing (VAC057; ClinicalTrials.gov: NCT02181088) utilized a recombinant prime-boost virus-vectored delivery platform that enabled *in situ* expression of RH5_FL by virally infected cells.^11^ This regimen was well tolerated and induced functional antibodies that exhibited cross-strain *in vitro* growth inhibition activity (GIA). However, although the levels of anti-RH5_FL serum immunoglobulin G (IgG) in this trial greatly exceeded those observed in naturally exposed African adults, they still only peaked at moderate concentrations of ∼9 μg/mL.^11^ Notably, our study in *Aotus* monkeys predicted these to fall below a protective immunological threshold, and we therefore elected not to proceed to efficacy testing by blood-stage controlled human malaria infection (CHMI).^18^ Indeed, protection in *Aotus* monkeys was strongly associated with anti-RH5_FL serum IgG antibody concentration and *in vitro* GIA measured using purified IgG,^17^ with high-level protection only achieved when using a recombinant RH5_FL protein-in-adjuvant formulation.^17^ Subsequently, we successfully expressed recombinant RH5_FL by using a *Drosophila* S2 stable cell line^19^^,^^20^ (a platform compatible with human delivery) and were therefore able to biomanufacture a soluble protein vaccine, called RH5.1.^21^ We hypothesized that this antigen, when presented with a potent adjuvant, could induce significantly higher levels of anti-RH5_FL antibodies than virus-vectored RH5 (VV-RH5) and therefore conducted the VAC063 phase I/IIa clinical trial (ClinicalTrials.gov: NCT02927145) to determine safety, immunogenicity, and efficacy against blood-stage CHMI of RH5.1 formulated in GlaxoSmithKline’s (GSK) adjuvant system AS01_B_.

## Results

### The RH5.1/AS01_B_ vaccine is safe and immunogenic

**Figure 1:**
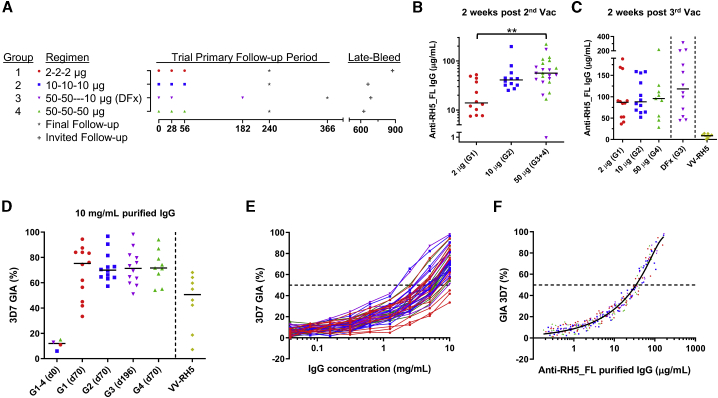
Antibody immunogenicity of RH5.1/AS01_B_ (A) Timing of immunizations and follow-up in groups 1–4. All antigen doses were formulated in 0.5 mL AS01_B_. (B and C) Median and individual anti-RH5_FL serum total IgG responses 14 days after two vaccinations (Vacs; day 42, B) and after three Vacs (day 70 or day 196, C). Both datasets were analyzed separately by Kruskal-Wallis test with Dunn’s multiple comparisons test; ∗∗p < 0.01. Historical data for the VV-RH5 vaccine^11^ were not included in the analysis and are shown for comparison only. (D) *In vitro* GIA of purified IgG assessed at 10 mg/mL against 3D7 clone *P. falciparum* parasites. Individual data and medians are shown for each group at the stated time-point; pooled sera were used for each group at baseline (day 0). Historical data for VV-RH5 were included as before. (E) Dilution series of purified IgG for all group 1–4 samples starting from 10 mg/mL. (F) Relationship between GIA data from the dilution series shown in (E) and concentration of anti-RH5_FL purified IgG used in the assay as measured by ELISA. A non-linear regression curve is shown for all samples combined (solid line, r^2^ = 0.96, n = 279). The EC_50_ (concentration of anti-RH5_FL polyclonal IgG that gives 50% GIA, dashed line) was calculated.

Fifty healthy adult volunteers were enrolled, across three sites in the United Kingdom, into the phase Ia arm of the VAC063 clinical trial, which assessed the RH5.1/AS01_B_ vaccine in an open-label, dose-escalation study (Figure 1A and S1). Three immunizations at monthly intervals with 2, 10, or 50 μg RH5.1 in 0.5 mL AS01_B_ (groups 1, 2, and 4) were compared with a delayed fractional dosing (DFx) regimen, where two monthly immunizations of 50 μg RH5.1 were followed by a third immunization at one-fifth dose (10 μg RH5.1) in 0.5 mL AS01_B_ and delayed to 6 months after the first immunization (group 3). Forty-seven of 50 (94%) volunteers received all scheduled vaccinations, and 44 completed follow-ups.

All vaccinations in groups 1–4 were well tolerated (Figure S2, Data S1A and S1B), and no safety concerns arose during the trial period. The reactogenicity of the vaccine was similar to that seen in previous malaria vaccine trials using the same adjuvant in healthy adults in the United Kingdom,^18^^,^^22^ with the booster vaccinations generally associated with a higher grading of systemic adverse events (AEs) and local redness (STAR Methods, safety analysis). A single occurrence of scalp psoriasis in one group-3 volunteer was regraded retrospectively as a suspected unexpected serious adverse reaction (SUSAR) on the basis of (1) protocol-specified AEs of special interest for the AS01_B_ adjuvant and (2) temporal onset 1 month after the second vaccination, making a causal relationship “possible” despite being unlikely clinically.

The anti-RH5 total IgG serum antibody concentration (in micrograms per milliliter) was assessed over time by ELISA against RH5_FL recombinant protein. Two priming immunizations induced antigen-specific IgG responses in all volunteers, with a clear dose response on day 42 (Figure 1B). By 2 weeks after the third monthly immunization (day 70), responses had equalized across the doses, with median (range) antibody levels of 91 (29–219) μg/mL in groups 1, 2, and 4 combined (n = 33). Two weeks after the final boost in the DFx regimen (day 196), responses trended higher, with median levels of 118 (45–314) μg/mL (n = 12) in group 3 (Figure 1C). Following each of the two booster immunizations, a burst of antibody-secreting cells (ASCs) was measured in the peripheral blood by ELISPOT; these were largely comparable across the vaccine doses and regimens (Figure S3A). Similar comparability was seen for interferon γ (IFN-γ) T cell responses in peripheral blood mononuclear cells (PBMCs), as measured by *ex vivo* ELISPOT and flow cytometry, 2 weeks after the third immunization (Figures S3B–S3D). In comparison with the previously reported first-generation VV-RH5 vaccine,^11^ the serum IgG concentrations induced by RH5.1/AS01_B_ were ∼10-fold higher, with the opposite trend observed for IFN-γ T cell responses (Figures 1C and S3B). We also assessed serum antibody isotypes and subclasses by ELISA. Here, the anti-RH5_FL response was mainly composed of IgG1, IgG3, IgA, and IgM with little detectable IgG2 or IgG4 (Figure S4A), and this profile was consistent over time and vaccine dose/regimen.

To assess functional antibody activity, sera were analyzed by the GIA Assay Reference Center at the NIH. IgG was purified from each sample prior to testing against 3D7 clone *P. falciparum* parasites at a starting concentration of 10 mg/mL total IgG and in the absence of complement. Pooled samples from groups 1–4 prior to vaccination (day 0) did not demonstrate any GIA above baseline. Samples from group 1–4 volunteers taken 2 weeks after the final immunization showed *in vitro* GIA, with median levels ranging between 70%–75% across all four groups (Figure 1D)—the highest levels of GIA reported in this assay following human vaccination.^11^^,^^18^^,^^23^, ^24^, ^25^ We confirmed that GIA decreased as purified IgG was diluted in the assay (Figure 1E). GIA was also related to RH5_FL-specific IgG concentration (Figure 1F), as seen previously for this antigen following VV-RH5 immunization,^11^ as well as following human immunization with other antigens, such as MSP1 and AMA1.^16^^,^^18^^,^^23^^,^^24^ For RH5.1/AS01_B_, the concentration of RH5_FL-specific polyclonal IgG required to give 50% GIA (EC_50_) was 34 μg/mL (95% confidence interval [CI], 33–36); this “quality” readout of vaccine-induced IgG was identical across the different dosing groups and regimens.

### The DFx regimen improves antibody response longevity

**Figure 2:**
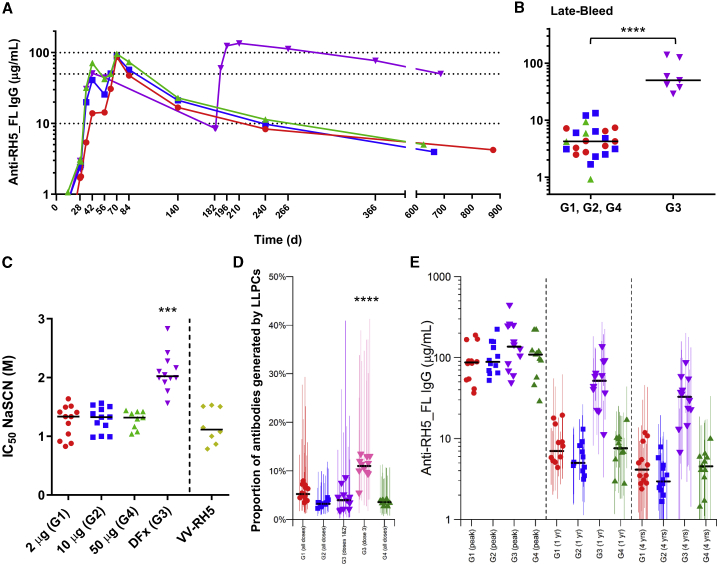
Assessment of the DFx regimen (A) Median anti-RH5_FL serum total IgG responses for groups 1–4 over time. Individual responses are shown in Figure S4B. (B) Median and individual anti-RH5_FL serum total IgG responses at the time of the late bleed. Statistical analysis was performed using a Mann-Whitney test, ∗∗∗∗p < 0.0001. (C) Avidity of serum total IgG responses 14 days after three immunizations (day 70 or day 196) was assessed by NaSCN displacement RH5_FL ELISA and is reported as the molar concentration of NaSCN required to reduce the starting optical density (OD) in the ELISA by 50% (IC_50_). Individual responses over time are shown in Figure S4C. Historical data for the VV-RH5 vaccine^11^ are shown for comparison. Kruskal-Wallis test with Dunn’s multiple comparison test, ∗∗∗p < 0.001 for group 3 versus groups 1, 2, and 4. (D) Estimated proportion of antibodies generated from LLPCs; for group 3, it was possible to provide separate estimates of the proportion following the first and second doses (purple) and following the third dose (pink). The long-lived response following the third dose in group 3 is significantly greater than in other groups; ∗∗∗∗p < 1 × 10^−6^ in all cases by one-sided t test. (E) Anti-RH5_FL antibody levels at peak and 1 and 4 years following the third vaccine dose. Peak antibody levels are based on the maximum measured values (day 70 or day 84 for groups 1, 2, and 4 and on day 196 or day 210 for group 3). Antibody levels at 1 and 4 years are based on model estimates and are presented with 95% CI.

All volunteers were followed up for 6 months after the final immunization, at which point it became apparent that the longevity of the anti-RH5_FL IgG antibody response was strikingly different following the DFx regimen (group 3) (Figures 2A and S4B). We therefore invited all volunteers who completed follow-up to return for a “late bleed” (approximately 1.5–2.5 years after their final vaccination), of whom 30 of 44 (68%) consented. Serum IgG responses at this late time point were significantly and ∼10-fold higher in group 3 compared with groups 1, 2, and 4. In group 3, the median (range) of the serum IgG response was 50 (29–140) μg/mL (n = 7), in contrast to the monthly dosing regimen of groups 1, 2, and 4, where the response was 4 (0.9–13) μg/mL (n = 23) (Figure 2B). The anti-RH5_FL serum IgG antibodies induced by the DFx regimen also showed significantly higher avidity (as measured by a sodium thiocyanate [NaSCN] displacement ELISA) compared with antibodies induced by the monthly dosing regimen or viral vectors (Figure 2C). Notably, the substantial increase in IgG avidity in the DFx group was coincident with administration of the delayed fractional third immunization and was maintained thereafter (Figure S4C), whereas avidity in the other groups did not change over time.

We observed no association between the magnitude of the RH5-specific IFN-γ T cell response and the improved maintenance and avidity of the serum IgG response induced by the DFx regimen. Peripheral blood IFN-γ T cell responses in group 3 decreased significantly from day 56 to the time of the delayed third immunization (day 182), consistent with a natural contraction of these responses over time (Figure S5A). These responses were of a similar magnitude across the groups and regimens 2 weeks after the final boost (Figure S3B) and still detectable ∼2 years later in the memory phase at the time of the late bleed (Figure S5B). More detailed phenotyping of the peripheral anti-RH5.1 specific CD4^+^ T follicular helper (Tfh) cell population, induced 2 weeks after the final boost, also revealed no difference between monthly vaccine dosing versus the DFx regimen (Figures S5C and S5D). However, we did note that the proportion of Tfh2 cells within the RH5.1-specific Tfh cell population, the subset regarded as the best at providing B cell help during generation of humoral responses,^26^ was significantly greater in the delayed fractional dose vaccinees (Figure S5E). This suggested a possible qualitative improvement in the Tfh cell response when using the DFx regimen as opposed to monthly dosing, even when the magnitude of the total Tfh cell response is comparable. Given these observations relating to Tfh2 cells, we hypothesized that the DFx regimen could favor induction of a greater proportion of long-lived plasma B cells (LLPCs) over short-lived plasma B cells compared with the monthly dosing regimen, leading to improved maintenance of the anti-RH5_FL serum IgG response over time. To address this, we undertook a modeling analysis of the serum IgG kinetics (Data S2A). This revealed that the estimated proportion of antibody generated by vaccine-induced LLPCs was significantly higher following the delayed fractional vaccine dose in group 3 (Figure 2D). A key factor driving the improved longevity of the vaccine-induced antibody response was thus an ∼2-fold quantitative increase in the proportion of antibody generated by the LLPC population. Moreover, modeling of the antibody response suggested that the improved longevity seen with the DFx regimen would be maintained up to 4 years (Figure 2E).

### Vaccinated and challenged volunteers show inhibition of parasite growth *in vivo*

**Figure 3:**
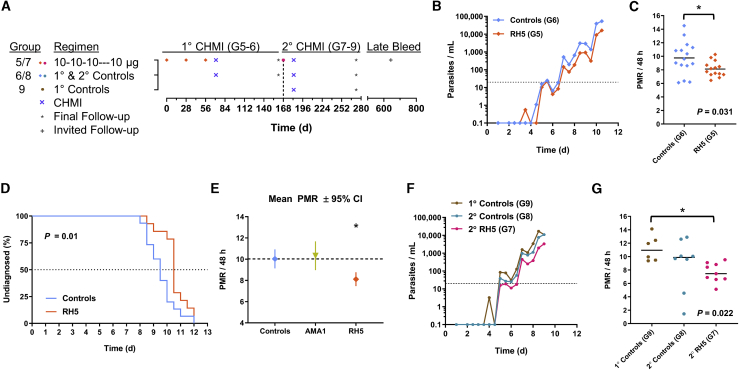
Results of primary and secondary blood-stage CHMI (A) Timing of immunizations, CHMIs, and follow-up in groups 5–9. All antigen doses were formulated in 0.5 mL AS01_B_. (B) qPCR data for the VAC063A phase IIa study; group 5 (n = 14) and group 6 (n = 15). Median parasitemia is shown over time for each group. The lower limit of quantification is indicated by the dotted line at 20 p/mL; values below this level are plotted for information only. Time = days after blood-stage CHMI. (C) Primary efficacy endpoint analysis of PMR, showing each individual plus the mean. Both datasets are normally distributed (D’Agostino-Pearson test); ∗p = 0.031 using two-tailed t test with Welch’s correction for non-equal variances (F test; p = 0.008). (D) Kaplan-Meier plot of time to diagnosis in days for the VAC063A study. Median time to patent parasitemia was 9.5 days for control individuals and 10.5 days for vaccinees. Secondary pre-specified efficacy analysis in the protocol compared time to diagnosis between the groups; p = 0.01, Mann-Whitney test. (E) Post hoc analysis combining the VAC063A dataset with the AMA1/AS01_B_ trial (VAC054) data^18^. Mean PMR ± 95% CI is shown for control individuals (n = 15 from VAC063A and n = 15 from VAC054), AMA1 vaccinees (n = 12 from VAC054), and RH5 vaccinees (n = 14 from VAC063A). ∗p < 0.05 for RH5 versus AMA1 and control individuals, using one-way ANOVA with Bonferroni correction for multiple comparisons. (F) qPCR data for the VAC063B phase IIa study shown as in (B); group 7 (n = 9), group 8 (n = 8), and group 9 (n = 6). (G) Secondary efficacy endpoint analysis of PMR, showing each individual, plus the median. ∗p = 0.022; Kruskal-Wallis test with Dunn’s multiple comparison test.

At the time of proceeding to the phase IIa arm of the VAC063 clinical trial, immunogenicity data were only available for groups 1, 2, and 4. Given that, following three monthly immunizations, no substantial differences were observed across the three doses of RH5.1, we elected to proceed with the 10-μg dose in the challenge study (called VAC063A). A new cohort of 17 volunteers (group 5) was recruited and vaccinated three times at monthly intervals with 10 μg RH5.1 formulated in 0.5 mL AS01_B_ (Figure 3A and S6), and these volunteers showed an AE profile comparable with that seen previously with the identical regimen in group 2 (Figures S7 and S2). Three vaccinees withdrew during the immunization phase; thus, 14 of these volunteers, along with 15 unvaccinated infectivity control individuals (group 6), subsequently underwent primary blood-stage CHMI with 3D7 clone *P. falciparum* parasites 14 days after the final immunization (day 70), using an established protocol^18^ (Figure S6).

All volunteers developed blood-stage parasitemia following CHMI and were subsequently drug treated according to a diagnostic algorithm based on patency by thick-film microscopy and/or pre-defined thresholds of parasites per mL (p/mL) blood by quantitative PCR (qPCR) and/or symptoms. The protocol pre-specified primary analysis for vaccine efficacy was comparison of the parasite multiplication rate (PMR) between the two groups. The PMR for each volunteer was calculated using a protocol pre-specified linear model fitted to log_10_-transformed qPCR data^27^ (Figures 3B and S8A; Data S1C). This analysis showed a significant reduction (p = 0.031) in the mean PMR between the two groups (Figure 3C), with a group 6 (infectivity control individuals) mean of 9.75 (95% CI, 8.39–11.11; standard deviation [SD] = 2.46) and a group 5 (vaccinees) mean of 8.12 (95% CI, 7.47–8.77; SD = 1.13). This reduction in PMR by ∼20% (from ∼10-fold per 48 h in control individuals to ∼8-fold in vaccinees) was also evident by significantly lower parasitemia at individual time points (Figure S8B) and would also predict an approximately 1-day delay to diagnosis at a threshold of 10,000 p/mL, as was indeed observed by the protocol pre-specified secondary efficacy analysis (Figure 3D). A post hoc analysis combined these PMR data with those from our previous VAC054 phase I/IIa clinical trial of the FMP2.1 AMA1/AS01_B_ protein/adjuvant vaccine that was assessed for efficacy in an identical manner.^18^ This combined analysis of 30 controls, 12 AMA1 vaccinees, and 14 RH5 vaccinees showed a significantly lower mean PMR in RH5 vaccinees compared with AMA1 vaccinees and the control individuals (Figures 3E and S8C). These data provide the first evidence of a significant *in vivo* effect of a malaria vaccine candidate on blood-stage parasite growth in humans following CHMI.

In light of these data, we invited group 5 and 6 volunteers to return for a re-challenge study (VAC063B). Eight control individuals consented (now referred to as group 8), along with nine vaccinees who were re-boosted with a single 10-μg dose RH5.1 formulated in 0.5 mL AS01_B_ approximately 16 weeks after their last immunization (now referred to as group 7). The AE profile of this delayed fourth RH5.1 immunization was comparable with the second immunization (Figure S7). Fourteen days after vaccinating group 7, all volunteers underwent secondary blood-stage CHMI, exactly as before, alongside six new malaria-naive primary CHMI control volunteers (group 9) (Figures 3A and S6). All volunteers developed blood-stage parasitemia following CHMI and were drug treated according to a diagnostic algorithm based on qPCR and/or symptoms, except for one group 8 volunteer who requested treatment on day of challenge (dC)+20 (Figures 3F, S8D, and S8E; Data S1D). qPCR-derived PMR again constituted the efficacy endpoint, and data were analyzed for all volunteers. The new group 9 primary controls showed parasite growth dynamics highly similar to the VAC063A study control individuals, with a mean PMR of 11.22 (95% CI, 9.20–13.23; SD = 1.92). Notably, one of the eight unvaccinated control individuals (group 8) undergoing secondary CHMI showed a dramatically reduced PMR (1.45-fold growth/48 h), with parasites not detectable by qPCR until dC+19. A second group-8 volunteer also showed evidence of reduced growth, but the remaining six volunteers in group 8 had PMRs in line with the primary control individuals (group 9). In contrast, 8 of 9 RH5 vaccinees showed PMRs that were lower than any of the primary controls, with an overall significantly reduced mean PMR of 7.62 (95% CI, 6.51–8.73; SD = 1.44) in comparison with group 9 (Figure 3G; adjusted p = 0.022 using Kruskal-Wallis test with Dunn’s multiple comparisons post hoc test). The observed PMRs in group 7 and 8 volunteers also correlated with their PMRs from the primary CHMI (Figure S8F). These data confirm the ability of RH5 vaccination to significantly affect *in vivo* blood-stage parasite growth in humans.

### Anti-RH5 antibody responses can boost following repeat vaccination after CHMI

**Figure 4:**
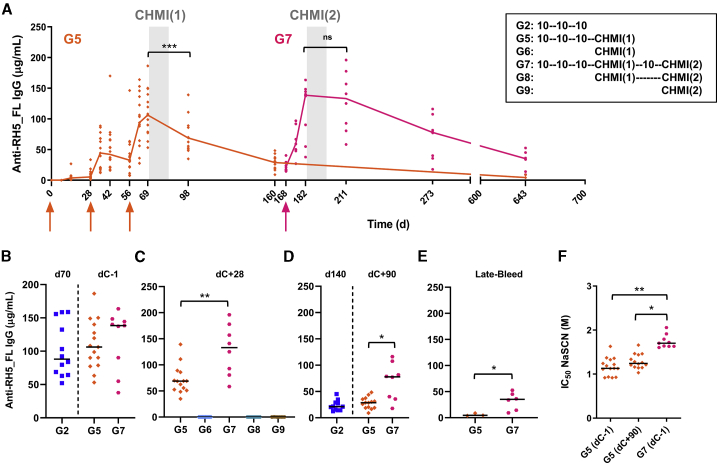
Antibody responses after CHMI and the fourth booster immunization (A) Median and individual anti-RH5_FL serum total IgG responses shown for groups 5 and 7 over time (note that a subset of group 5 vaccinees became group 7; Figure S6). Gray shading indicates periods of CHMI, and arrows indicate Vacs. The legend indicates the sequence of 10-μg RH5.1/AS01_B_ Vacs as well as the first and second CHMIs, as relevant to each group. (B–E) Individual and median responses are shown for the indicated groups and time points. dC, day of challenge. ∗p < 0.05, ∗∗p < 0.01; Wilcoxon matched-pairs signed-rank test in (C) and (D). ∗p < 0.05, Mann-Whitney test in (E). (F) Avidity of serum total IgG responses in groups 5 and 7 at the indicated time points was assessed by NaSCN displacement RH5_FL ELISA. ∗p < 0.05, ∗∗p < 0.01; Friedman test for paired samples with Dunn’s multiple comparisons test.

We next assessed the anti-RH5_FL serum antibody response over the course of both CHMI studies (Figure 4A). The levels induced in group 5 vaccinees by dC-1 (prior to the first CHMI) were highly similar to those measured in the original group 2 vaccinees on day 70, confirming comparability across these replication cohorts (Figures 4B and S9A). Also, despite CHMI and as seen for group 2, the peak responses in group 5 soon waned, resulting in significantly lower responses by 28 days after CHMI (dC+28) (Figure 4A). The fourth RH5.1/AS01_B_ immunization nevertheless re-boosted these antibodies, which trended toward higher levels by dC-1 for the secondary CHMI (Figure 4B). Intriguingly, after the second CHMI, there was no significant change in anti-RH5_FL serum IgG levels between dC-1 and dC+28 for the group 7 vaccinees. These responses were significantly higher than when measured in the same individuals 28 days after their primary infection (Figures 4A and 4C). A possible explanation is that they were naturally boosted or maintained by the second CHMI. However, there was no evidence of natural boosting in the primary CHMI (given comparable antibody levels in groups 2 and 5 regardless of CHMI, approximately 3 months after their last immunization) (Figure 4D). There was also no detectable induction of *de novo* anti-RH5_FL serum IgG responses in control volunteers after one or two rounds of CHMI (Figure 4C), consistent with RH5’s known poor immunogenicity in the context of natural malaria infection^9^, ^10^, ^11^, ^12^^,^^28^ and primary CHMI.^29^ Moreover, the anti-RH5_FL serum IgG in group 7 was also better maintained over the next 1–2 years (Figures 4D and 4E), closely mirroring our previous observations in group 3 using the DFx regimen. These data therefore suggest that the fourth delayed (but non-fractional) immunization in group 7 led to improved longevity of the anti-RH5_FL serum IgG response. Consistent with this, the anti-RH5_FL IgG responses in group 5/7 volunteers also showed a significant increase in avidity immediately after the fourth immunization (Figure 4F), just as we observed previously with the DFx regimen (Figure 2C).

### *In vitro* growth inhibition correlates with *in vivo* growth inhibition

**Figure 5:**
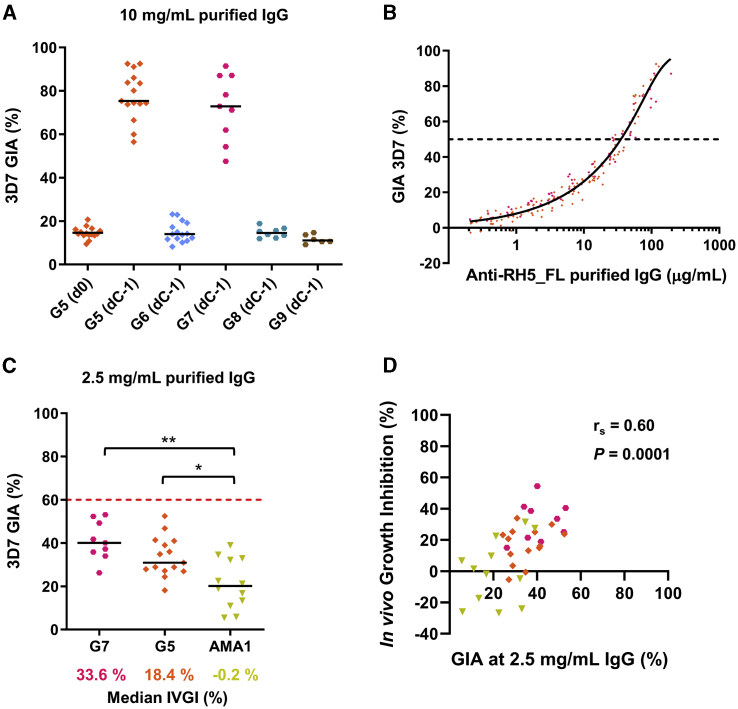
Analysis of *in vitro* GIA versus IVGI (A) *In vitro* GIA of purified IgG assessed at 10 mg/mL against 3D7 clone *P. falciparum* parasites. Individual data and medians are shown for each group (Figure 3A) at the stated time points. (B) Relationship between GIA and concentration of anti-RH5_FL purified IgG used in the assay, as measured by ELISA. A non-linear regression curve is shown for all samples combined (solid line, r^2^ = 0.97, n = 180). The EC_50_ (dashed line) was calculated. (C) *In vitro* GIA as in (A), using purified IgG assessed at 2.5 mg/mL. Historical data from the AMA1/AS01_B_ (VAC054) trial^18^ are included. The median percent IVGI observed in each group following blood-stage CHMI is indicated below the graph. The red dashed line at 60% GIA indicates the threshold level required for protection in *Aotus* monkeys.^17^^,^^30^ ∗p < 0.05, ∗∗p < 0.01; Kruskal-Wallis test with Dunn’s multiple comparisons test. (D) Correlation of % IVGI observed in each individual following blood-stage CHMI versus their individual *in vitro* GIA measured at dC−1 using 2.5 mg/mL purified IgG. Spearman’s rank correlation coefficient and p value are shown; n = 35. Colored symbols are the same as those used in (C) for AMA1, group 5 (G5), and G7.

We next assessed functional *in vitro* GIA for groups 5–9 as done previously, using purified IgG from the dC-1 time point at a starting concentration of 10 mg/mL. There was no GIA above baseline prior to primary or secondary challenge of any control volunteers, in contrast to high levels in groups 5 and 7 (Figure 5A). Titration of IgG again showed that GIA was related to RH5_FL-specific IgG concentration, with an antigen-specific EC_50_ of 35 μg/mL (95% CI, 33–37), identical to that observed previously in groups 1–4 (Figure 5B). To assess the relationship between *in vitro* GIA and *in vivo* outcome following CHMI, we calculated the *in vivo* growth inhibition (IVGI) for each vaccinated volunteer as the percentage of reduction in the PMR in each individual relative to the mean in the malaria-naive control group. In this post hoc analysis, we included all vaccinated volunteers assessed over three blood-stage CHMI studies (group 5, group 7, and our previously reported AMA1/AS01_B_ vaccine trial^18^). Data from previous *Aotus* monkey *P. falciparum* challenge studies suggested that levels of *in vitro* GIA of more than 60% at 2.5 mg/mL purified IgG are associated with a protective outcome following blood-stage vaccination.^17^^,^^30^ Consistent with this, the median levels of IVGI in each group increased as their *in vitro* GIA increased when using 2.5 mg/mL purified IgG in the assay, although no individual measured more than 60% (Figure 5C). Moreover, there was a highly significant correlation between the *in vitro* assay of GIA and IVGI (Figure 5D), as also seen for the same analysis of GIA versus time to/day of diagnosis (DOD) (Figure S9B). Given that the GIA assay uses a normalized concentration of purified IgG, we also related these results to each individual’s serum IgG concentration (Figures S9C and S9D), and the correlation remained highly significant (Figure S9E). These data provide the first strong evidence that the *in vitro* GIA assay can correlate with outcome against blood-stage *P. falciparum* CHMI in vaccinated humans; this is highly consistent with previous non-human primate studies that also assessed MSP1, AMA1, or RH5 vaccine efficacy against blood-stage malaria challenge.^17^^,^^30^^,^^31^

### The anti-RH5 plasma IgA1 response is associated with delayed time to diagnosis

Despite observing a significant correlation between *in vitro* GIA and IVGI after blood-stage CHMI, we hypothesized that the vaccine-induced polyclonal anti-RH5 antibody may have additional Fc-mediated effector functionalities that are not measured in the standardized GIA assay but still relevant to mediating protection against merozoite invasion. The GIA assay provides a neutralization-type readout using purified IgG lacking immune cells, complement, and other vaccine-induced antibody isotypes/subclasses. To address this limitation of the GIA assay, we also undertook an exploratory analysis using a systems serology approach.^32^^,^^33^ Here we initially measured a diverse array of antibody features and functions prior to using these datasets for computational modeling. Our aim was to decipher the most important antibody feature sets that are associated with reduced parasite growth *in vivo*, identifying key immune parameters for future experimental vaccine testing.

**Figure 6:**
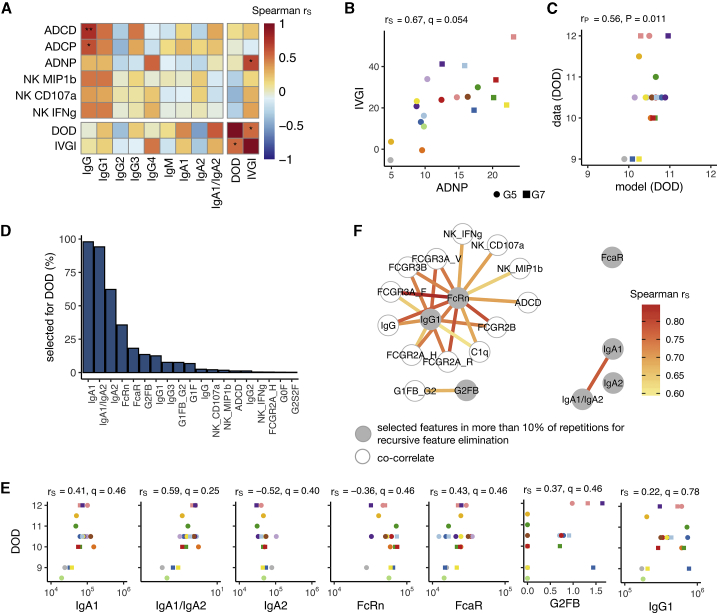
Systems serology analysis of CHMI outcome in RH5 vaccinees (A) Correlation heatmap showing the Spearman rank correlation coefficients (r_S_) between Fc functions and titers as well as the rank correlation of the features to the CHMI readouts of DOD and IVGI. ∗q < 0.1, ∗∗q < 0.01 (Benjamini-Hochberg procedure for multiple testing correction; the correction was done within the groups of comparison; 54 comparisons for Fc functions/titer, 40 for features/DOD, and 40 for features/IVGI). (B) Scatterplot to show the relationship between ADNP score and IVGI. Each color corresponds to one volunteer, and the shape indicates the group: G5 (n = 13) or G7 (n = 7). (C) Prediction for the random forest regression model plotted against the data for DOD. The model was obtained using leave-one-volunteer-out cross-validation. The Pearson correlation coefficient (r_P_) and p value are shown. (D) The antibody features in the predictive model for DOD are ranked according to how often they were chosen for 100 repetitions of recursive feature elimination (RFE) in the leave-one-volunteer-out cross-validation. (E) Graphs showing DOD versus systems serology assay data for the seven antibody features that were chosen in more than 10% of the elimination procedures (D). (F) The co-correlates network shows the pairwise correlation of features. The nodes correspond to features and the edges to Spearman rank correlations between the features. Only significant correlations (Benjamini-Hochberg q < 0.05) between features that are selected in more than 10% of the RFE procedures and all other features are shown. G2FB Fc glycan, fucosylated 2-galactose with bisecting N-acetylglucosamine.

Initially, we sought to (1) quantify the post-vaccination plasma levels of RH5-specific antibodies of each major isotype and subclass; (2) evaluate the capacity of anti-RH5 antibody to bind Fc receptors (FcRs) and activate natural killer (NK) cells, neutrophils, monocytes, and the complement cascade; and (3) characterize the glycosylation profile of the anti-RH5 IgG Fc domains, which is known to influence these Fc-mediated functions.^34^ We then performed computational analyses with these *in vitro* assay datasets for RH5 vaccinee (groups 5 and 7) dC-1 plasma samples and the DOD and IVGI efficacy measures (Figures 6A and S10A; Data S2B). Correlation analysis confirmed a positive relationship between the DOD and IVGI efficacy measures (as would be expected) and identified a strong positive correlation between antibody-dependent neutrophil phagocytosis (ADNP) and IVGI (Figures 6A and 6B). Computational analysis was subsequently able to generate a model that could predict values within a cross-validation framework that were consistently and significantly correlated (p = 0.011) with the DOD efficacy readout data measured in the trial (Figure 6C). The same modeling analysis using the IVGI efficacy readout but marginally missed significance (p = 0.053) (Figures S10B–S10E).

We therefore proceeded to identify the set of *in vitro* systems serology features that are most important for the DOD model. These are shown in decreasing order of importance (Figure 6D), along with correlations between DOD and the top seven features (Figure 6E): magnitude of the anti-RH5 IgA1 response, IgA1:IgA2 ratio, magnitude of anti-RH5 IgA2, binding of RH5-specific antibody to FcRn, anti-RH5 antibody binding to FcαRI, proportion of RH5-specific IgG Fc glycans that are fucosylated 2-galactose with bisecting N-acetylglucosamine (G2FB), and magnitude of anti-RH5 IgG1. Although the FcRn and IgG1 readouts are highly correlated with many other features, the IgA readouts, FcαRI binding, and G2FB glycan results show greater independent importance (Figure 6F). These data therefore indicate a relationship between an increased anti-RH5 IgA1 response and delayed time to diagnosis and highlight this response for future investigation.

## Discussion

Development of an effective blood-stage vaccine against *P. falciparum* has proved challenging. Historical protein-in-adjuvant vaccine candidates against the MSP1, AMA1, or MSP3-GLURP antigens have shown no efficacy,^35^^,^^36^ strain-specific efficacy,^37^ or low-level efficacy^38^ in phase IIb field trials in African children or infants. The two vaccines to report efficacy signals in the field underwent retrospective testing by CHMI in adults, but no differences were observed between vaccinees and control individuals,^18^^,^^39^ suggesting that the challenge model may pose a higher bar for efficacy even when using vaccine-homologous parasites. Consistent with this, no other blood-stage *P. falciparum* subunit vaccine candidate has significantly reduced the *in vivo* PMR (in vaccinees compared with control individuals) following CHMI in malaria-naive adults, including other vaccines formulated in AS01_B_ (ruling out an adjuvant effect).^25^^,^^40^, ^41^, ^42^ Reduced PMRs (as measured by qPCR and compared with malaria-naive adults) are nevertheless observed in semi-immune African adults following natural infection^43^ or CHMI.^44^^,^^45^ Consequently, our data with RH5.1/AS01_B_ provide the first demonstration of a significant *in vivo* effect on PMR following vaccination and primary blood-stage CHMI, defining a milestone for the blood-stage malaria vaccine field. These findings were confirmed when a greater significant effect on *in vivo* PMR was also seen in a subset of nine vaccinees who received a fourth booster vaccination followed by secondary CHMI. However, the overall vaccine-induced effect was modest and only led to a 1- to 2-day delay in time to diagnosis, as would be predicted when reducing the growth rate from an average of ∼10-fold to ∼7- to 8-fold per 48 h.^3^ Improved next-generation vaccine immunogens and/or formulations targeting RH5 or its invasion complex are now required to achieve protection via more substantial reductions in PMR.

We also identified the *in vitro* assay of GIA as a highly significant predictor of IVGI, following a post hoc analysis of data across three blood-stage *P. falciparum* CHMI studies, using vaccines targeting RH5 or AMA1. This quantitative association was nearly identical to that we reported previously following vaccination and *P. falciparum* challenge of *Aotus* monkeys.^17^ More recently, we confirmed this association as a mechanistic correlate in *Aotus* monkeys; i.e., one that can cause *in vivo* protection via passive transfer of a GIA-positive, RH5-specific IgG monoclonal antibody (mAb),^46^ with similar results observed in humanized mice.^47^ Full protection of *Aotus* monkeys required a serological threshold level of GIA,^17^^,^^30^ which we approached in this trial of adults in the United Kingdom but did not exceed. Nonetheless, our analysis of RH5.1/AS01_B_ now provides defined benchmark levels of GIA and IVGI in humans; these can guide interpretation of future clinical trials of populations living in malaria-endemic areas as well as endeavors to develop improved RH5-based vaccines.

Our systems serology analysis also identified other antibody feature sets, most frequently including the plasma anti-RH5 IgA1 response, that are associated with challenge outcome. These data provide important evidence to support the notion that “neutralization” of erythrocyte invasion by IgG (as measured by the GIA assay) may not be the only relevant component of functional vaccine-induced immunity against the merozoite. The relationship between ADNP and IVGI implies that the role of IgA1 could be related to activation of neutrophils, the most abundant immune cells in the blood and, therefore, intuitively the most likely cells to be in close proximity to a rapidly invading merozoite. Previous work has reported that antibody-dependent respiratory burst (ADRB) activity from neutrophils can be associated with clinical immunity to malaria.^48^ Further studies and novel assay development are now required to interrogate mechanism-of-action hypotheses for vaccine-induced RH5-specific IgA1, replicate these findings, and synthesize the relative contributions of different antibody effector functions against the blood-stage merozoite.

Our data also confirm the quantitative challenge of achieving high-level antibody-mediated protection against the merozoite. Protection of *Aotus* monkeys necessitated more than 300 μg/mL anti-RH5 IgG or more than 60% GIA using 2.5 mg/mL purified IgG in the assay,^17^ and, although we approached these immunological thresholds in humans, a minimum 3-fold quantitative improvement is still required to exceed them. Nevertheless, the relationship reported here in humans between *in vitro* GIA and IVGI following CHMI is highly similar to that we reported previously for *Aotus* monkeys vaccinated with RH5 and then challenged with blood-stage *P. falciparum*,^17^ suggesting a strong degree of comparability across these two species.

Addressing the challenge of how to further improve the quantity and/or quality of the vaccine-induced anti-merozoite antibody response is now of strategic importance. First, evidence from clinical trials of pre-erythrocytic malaria subunit vaccine candidates suggests that substantially higher antibody responses (in the range of 5- to 10-fold) are observed in the target population (African infants) compared with malaria-naive adults.^49^, ^50^, ^51^ Consequently the first phase Ib data of the VV-RH5 vaccine and RH5.1 protein vaccine in African infants are awaited eagerly (ClinicalTrials.gov: NCT03435874 and NCT04318002). These will provide important insight regarding whether the efficacy of RH5-based vaccines is potentially underestimated by CHMI in adults in the United Kingdom because of relatively lower levels of antibody response. A second strategy to improve RH5-based vaccines would be to incorporate the other, more recently described antigens CyRPA and RIPR, which associate with RH5 to form a heterotrimeric protein complex; like RH5, both of these antigens are highly conserved and essential and can induce high levels of growth-inhibitory antibodies following vaccination of animals.^6^^,^^52^ Finally, rational improvements of the design and delivery of the RH5 immunogen should also be explored. Strategies to achieve substantial improvements in the quantity and/or quality of vaccine-induced polyclonal anti-RH5 IgG would include analysis of vaccine-induced anti-RH5 human mAbs^53^ to inform structure-based vaccine design^54^ coupled with improved delivery of novel RH5 immunogen arrays on virus-like particles.^55^ In-depth analysis of the RH5 antibody epitope specificities induced by the RH5.1/AS01_B_ vaccine, linked to structure and functional outcome, will be essential to guide these future approaches to improve the vaccine by rational design.

However, even when protective levels of antibody are achieved, these levels of functional antibody must also be maintained if they are to provide a useful duration of immunity. Our CHMI data suggest that RH5-based vaccines cannot rely on “natural boosting” following malaria exposure, fully consistent with the poor immunogenicity of RH5 in the context of malaria infection. Nevertheless, given the potential of malaria infection to have immunomodulatory effects on B cell responses,^56^ our finding that CHMI did not negatively affect vaccine-induced antibody titers or the ability to boost existing anti-RH5 IgG responses is encouraging for future vaccine use in malaria-endemic areas. Furthermore, our assessment of the DFx regimen builds on the experiences of the RTS,S/AS01 vaccine,^57^ aligns with emerging strategies for seasonal malaria vaccination,^58^ and offers significant promise for induction of more durable malaria immunity. Although not definitive, our data from the delayed fourth RH5.1/AS01_B_ booster vaccination in the vaccinated and challenged cohort also suggest that the delay (as opposed to dose fractionation) is the major contributor to this effect. This is also consistent with our observations that RH5.1 vaccine doses ranging from 2–50 μg showed comparable immunogenicity, albeit after monthly as opposed to DFx dosing.

Regardless, the DFx regimen showed higher average anti-RH5 IgG responses at the peak as well as significantly improved anti-RH5 IgG avidity and longevity, with modeling of the antibody kinetics at 4 years suggesting that an ∼10-fold quantitative improvement would be maintained (compared with the monthly dosing regimen). Further quantitative and qualitative analyses of the underlying cellular responses are now warranted in the DFx regimen vaccinees, given that our data indicate (1) that an increased proportion of the antibody response is generated by the LLPC population and (2) a RH5-specific Tfh2 cell skew, again similar to a recent report for RTS,S/AS01.^59^ Notably, improved anti-RH5 IgG avidity did not affect *in vitro* GIA; however, this observation is consistent with recent human anti-RH5 mAb data indicating that speed of binding (i.e., antibody on-rate) is a key determinant of GIA potency as opposed to off-rate/overall affinity or strength of binding.^53^ Nevertheless, improved antibody avidity is associated with increased anti-sporozoite vaccine efficacy against CHMI,^57^ suggesting that regimen optimization could add significant value to ongoing efforts to improve other malaria subunit vaccines. Our data also pose a wider challenge to the traditional dosing and boosting regimens used for human vaccine development. Substantial gains in vaccine-induced efficacy and response longevity could be achieved via simple modulation and optimization of dose and delivery regimen.

### Limitations of study

Our study has certain limitations. First, although powered for a certain effect size in the primary CHMI, the trial still has a relatively small sample size. This is fairly common for early-phase malaria vaccine clinical trials that seek to demonstrate proof of concept for vaccine safety, dosing, regimen, and/or efficacy using the CHMI model. Also, some analyses, including secondary CHMI and late bleed to assess antibody response longevity after ∼1.5–2.5 years, were amended into the trial protocol in light of promising data arising in the original part of the study. This relied on volunteers being able and willing to return to the trial and led to an inevitable reduction in sample size. Second, although our analysis of efficacy in the primary CHMI study was pre-specified, our investigation of immune responses that were associated with *in vivo* outcome following CHMI was post hoc and exploratory. Our conclusions could therefore be strengthened in the future by a replication cohort with larger sample size to assess vaccine efficacy, alongside pre-specified analyses of the immunological responses identified here that were associated with CHMI outcome measures. Third, our systems serology assays to measure antibody function utilized recombinant RH5 protein as opposed to merozoites; although technically challenging, on-going development of these assays to incorporate whole parasites should be an improvement. Finally, blood-stage CHMI remains a model to investigate blood-stage vaccine efficacy and was used here in malaria-naive adults in the United Kingdom. It is ultimately imperative to understand how these observations, using the CHMI model, relate to efficacy against natural mosquito-borne infection in African infants, the target population for such a vaccine.

## STAR★Methods

### Key resources table

**Table:** 

REAGENT or RESOURCE	SOURCE	IDENTIFIER
**Antibodies**		

Alkaline phosphatase-conjugated goat anti-human IgG (γ-chain)	Sigma	A3187
biotin-conjugated mouse anti-human IgG1 Fc (clone HP6070)	ThermoFisher	MH1515
biotin-conjugated mouse anti-human IgG2 (clone HP6002)	ThermoFisher	05-3540
biotin-conjugated mouse anti-human IgG3 (clone HP6050)	Sigma	B3523
biotin-conjugated mouse anti-human IgG4 (clone HP6025)	Sigma	B3648
Alkaline phosphatase-conjugated goat polyclonal anti-human IgA α-chain	Sigma	A9669
Biotin-conjugated goat polyclonal anti-human IgM μ-chain	Sigma	B1265
Polyvalent goat-anti human Ig	ThermoFisher	H17000
Anti-human IgG (γ-chain) antibody conjugated to alkaline phosphatase	Merck	401442
IFN-γ capture monoclonal antibody (1-D1K)	Mabtech	3420-2A
IFN-γ biotinylated detection monoclonal antibody (7-B6-1)	Mabtech	3420-2A
Anti-human CD183-APC (clone: 1C6/CXCR3)	BD Biosciences	550967
Anti-PD1-BV421 (clone: EH12.2H7)	Biolegend	329920
Anti-CD14-BV510 (clone: M5E2)	Biolegend	301842
Anti-CCR6-BV711 (clone: G034E3)	Biolegend	353436
Anti-CD3-BV605 (clone: UCHT1)	Biolegend	300460
Anti-CD137-BV650 (clone: 4B4-1)	Biolegend	309828
Anti-CD45RO-BV785 (clone: UCHL1)	Biolegend	304234
Anti-CD19-BV510 (clone: SJ25C1)	BD Biosciences	562947
Anti-CD69-PE-Cy5 (clone: FN50)	BD Biosciences	555532
Anti-CXCR5-AF700 (clone: RF8B2)	BD Biosciences	565191
Anti-CD4-APC-H7 (clone: SK3)	BD Biosciences	641398
Anti-OX40-PE (clone: L106)	BD Biosciences	340420
Anti-CD25-PE-Cy7 (clone: 2A3)	BD Biosciences	335824
Anti-IFN-γ-PerCP-Cy5.5 (clone: 42.B3)	Life Technologies	45-7319-42
Anti-CD8-PE-TR (clone: 3B5)	Life Technologies	MHCD 0817
Anti-ICOS-biotin (clone: ISA-3)	Invitrogen	13-9948-82
APC-Cy7 anti-huCD14	BD Biosciences	557831
PE-Cy7 anti-huCD3	BD Biosciences	563423
PE-Cy7 anti-huCD56	BD Biosciences	335791
BV421 anti-huMIP-1b	BD Biosciences	562900
Pacific Blue anti-huCD66b	Biolegend	305112
APC-Cy7 anti-huCD3	Biolegend	300426
BV605 anti-huCD107a	Biolegend	328634
PE anti-huIFN-g	Biolegend	506507
FITC-conjugated, goat anti-guinea pig complement C3 polyclonal antibody	MP Biomedical	0855385
PE-conjugated secondary antibodies total huIgG	Southern Biotech	9040-09
PE-conjugated huIgM	Southern Biotech	9020-09
PE-conjugated huIgA1	Southern Biotech	(#9130-09),
PE-conjugated huIgA2	Southern Biotech	9140-09
PE-conjugated huIgG1	Southern Biotech	9052-09
PE-conjugated huIgG2	Southern Biotech	9070-09
PE-conjugated huIgG3	Southern Biotech	9210-09
PE-conjugated huIgG4	Southern Biotech	9200-09

**Biological samples**		

Human blood products	This study	ClinicalTrials.gov NCT02927145
*P. falciparum* (clone 3D7) parasitized red blood cells	QIMR Berghofer Medical Research Institute, Brisbane	^60^
FCS	Labtech	N/A

**Chemicals, peptides, and recombinant proteins**		

Recombinant RH5.1 protein (full-length PfRH5 aaE26-Q526 based on 3D7 clone of *P. falciparum* with C-terminal E-P-E-A C-tag)	University of Oxford	^21^
AS01_B_ adjuvant	GlaxoSmithKline (GSK)	N/A
Dimethyl sulfoxide (DMSO)	Sigma	D2650
RH5.1 peptides, 20mers overlapping by 10aa	NEO Scientific, USA	
Brefeldin A	Life Technologies	00-4506-51
Staphylococcal enterotoxin B	Sigma	S-4881
Cytofix/Cytoperm	BD Biosciences	554714
Live/Dead Aqua	Invitrogen	L34966
Anti-ICOS-biotin (clone: ISA-3)	Invitrogen	13-9948-82
Sodium thiocyanate (NaSCN)	Sigma	251410
Biotinylated RH5	University of Oxford	
Guinea pig complement	CedarLane	CL4051
Gelatin veronal buffer containing calcium and magnesium	Boston Bioproducts	IBB-300
GolgiStop	BD	554724
FCGR2A avi tag	Duke Human Vaccine Institute Protein Production facility	N/A
FCGR2B avi tag	Duke Human Vaccine Institute Protein Production facility	N/A
FCGR3A avi tag	Duke Human Vaccine Insitute Protein Production facility	N/A
FCGR3B avi tag	Duke Human Vaccine Insitute Protein Production facility	N/A
Purified human C1q protein	Sigma	C1740
EZ-Link-NHS-LC-LC-Biotin	Pierce	A35358
Streptavidin-PE	Prozyme	PJ31S
Streptavidin coated microspheres	New England Biolabs	S1420S
IdeZ enzyme	New England Biolabs	P0770S
PNGase F	Applied Biosystems	A28404
Bir A Ligase	Avidity	BirA500
Streptavidin-BB515	BD	564453

**Critical commercial assays**		

QIAGEN DSP DNA Midi Kit	QIAGEN	937255
RosetteSep human NK cell enrichment cocktail	StemCell	15065
Glycan Assure kit	Thermo	A28676
TaqMan Universal PCR master mix	Applied Biosystems	4440038

**Experimental models: Cell lines**		

RH5.1 production cell line: *Drosophila melanogaster* Schneider 2 (S2) cell line	ExpreS2ion Biotechnologies, Denmark	^21^
THP-1 cells	ATCC	TIB-202

**Experimental models: Organisms/strains**		

*P. falciparum* 3D7 clone parasites	GIA Reference Center	N/A

**Oligonucleotides**		

Forward primer (*5′GTAATTGGAATGATAGG* *AATTTACAAGGT 3′)*	Applied Biosystems	N/A
Reverse primer (*5′ TCAACTACGAACGTTTT* *AACTGCAAC 3′)*	Applied Biosystems	N/A
TaqMan FAM-NFQ-MGB Probe (*5′ FAM- AACAATTGGAGGGCAAG-NFQ-MGB 3′)*	Applied Biosystems	4316033

**Recombinant DNA**		

Parasite or Plasmid DNA qPCR standards	University of Oxford	N/A

**Software and algorithms**		

ABI StepOne Plus machine with v2.3 software	Applied Biosystems	4376598
Gen5 ELISA software v3.04	Biotek, UK	N/A
GraphPad Prism version 8.3.1 for Windows	GraphPad Software Inc.	N/A
FlowJo v10, Treestar	Treestar	N/A
R notebook (analysis_Rh5.R.md)		Data S2
BD FACSDiva8.0 Software	BD Biosciences	N/A
R version 3.3.3	R foundation for Statistical Computing, Vienna, Austria	N/A

**Other**		

ABI StepOne Plus machine	Applied Biosystems	N/A
Biotek Elx808 reader	Biotek	N/A
Fortessa X20 flow cytommeter	BD Biosciences	N/A
QIAsymphony SP robot	QIAGEN	N/A
ELISPOT counter	Autoimmun Diagnostika, Germany	N/A
Agilent 1260 HPLC system	Agilent, UK	N/A
Bio-Monolith Protein G column	Agilent	N/A
Intellicyt iQue Screener PLUS flow cytometer	Intellicyt	N/A
ABI 3500xL Genetic Analyzer + software	Applied Biosystems	N/A

### Resource availability

#### Lead contact

Further information and requests for resources and reagents should be directed to and will be fulfilled by the lead contact, Simon Draper (simon.draper@ndm.ox.ac.uk).

#### Materials availability

This study did not generate new unique reagents.

#### Data and code availability

The published article includes the R notebook (analysis_Rh5.Rmd) for the Systems Serology analysis (see Data S2B).

### Experimental model and subject details

#### VAC063 Study Design, Subject Details and Study Procedures

*RH5.1/AS01*_*B*_ *Vaccine.* The RH5.1 protein consists of the entire full-length ectodomain of the PfRH5 antigen (amino acids E26 – Q526) with the sequence based on the 3D7 clone of *P. falciparum*. The vaccine was produced from a stable *Drosophila melanogaster* Schneider 2 (S2) cell line, and also contained an N-terminal 18 αα BiP insect signal peptide (MKLCILLAVVAFVGLSLG) that is cleaved off as the protein is secreted from the cell, and a C-terminal four amino acid (E-P-E-A) “C-tag” used for affinity purification.^20^ The cell line system called ExpreS^2^ was provided by ExpreS^2^ion Biotechnologies in Denmark.^19^ All four putative N-linked glycosylation sequons (N-X-S/T) were mutated Thr to Ala – as performed for a previous PfRH5 protein vaccine produced in mammalian HEK293 cells and tested in rabbits^9^ and *Aotus* monkeys.^17^

RH5.1 was manufactured under Good Manufacturing Practice (GMP) by the Clinical Biomanufacturing Facility (CBF) in Oxford in 2015 and protein vaccine stability was monitored over time, as previously reported in detail.^21^ The doses used in this study were 2 μg, 10 μg and 50 μg (nominal doses as the actual doses may have varied slightly dependent on dilution, mixing and administration). The RH5.1 vaccine was stored at the clinical facilities between −70°C and −90°C. The adjuvant system AS01_B_ was provided by GlaxoSmithKline (GSK) and stored as a 0.5 mL extractable liquid in a mono-dose glass vial at +2 to +8°C before use. The RH5.1 protein and AS01_B_ adjuvant were mixed in the clinic prior to use and injected (within 1 h) intramuscularly into the non-dominant deltoid (carried out as per local Standard Operating Procedures MC031 and VC002).

*Study Design and Approvals.* VAC063 was a first-in-human, open-label, non-randomized, multi-center, dose escalation Phase I/IIa clinical trial evaluating the safety, immunogenicity and efficacy of the recombinant blood-stage malaria protein RH5.1 formulated in GSK’s adjuvant system AS01_B_.^21^ The study was conducted in the UK at the Centre for Clinical Vaccinology and Tropical Medicine (CCVTM), University of Oxford (Phase I/IIa), the NIHR Wellcome Trust Clinical Research Facility (WTCRF) in Southampton (Phase Ia) and Guys and St Thomas’ NIHR Clinical Research Facility, London (Phase I/IIa). All volunteers recruited were healthy, malaria-naive adults (males and females) aged between 18 and 45 years. Vaccine efficacy was assessed using blood-stage controlled human malaria infection (CHMI) by injection of parasitized red blood cells,^18^ as described below. Blood-stage CHMI and follow-up for all challenged volunteers took place at the CCVTM at the University of Oxford. There were nine study groups across two phases of the trial, with a total of 88 volunteers enrolled (33 male and 55 female); 50 in Phase Ia, 38 in Phase IIa (see Figures S1 andS6 for more detail). The trial was registered on ClinicalTrials.gov (NCT02927145) and was conducted according to the principles of the current revision of the Declaration of Helsinki 2008 and in full conformity with the ICH guidelines for Good Clinical Practice (GCP). All volunteers signed written consent forms, and consent was checked to ensure volunteers were willing to proceed before each vaccination and prior to CHMI.

The study received ethical approval from the UK NHS Research Ethics Service (Oxfordshire Research Ethics Committee A, Ref 16/SC/0345), and was approved by the UK Medicines and Healthcare products Regulatory Agency (Ref 21584/0362/001-0011).

The primary objectives of the trial were to i) assess the safety of the RH5.1/AS01_B_ vaccine in healthy volunteers at different doses; ii) assess the *in vitro* GIA against 3D7 clone *P. falciparum* parasites of IgG purified from the serum of vaccinees; and iii) to establish whether the RH5.1/AS01_B_ vaccine could demonstrate a reduced parasite multiplication rate (PMR) in vaccinated subjects compared to infectivity controls in a blood-stage CHMI model (PMR being the primary efficacy endpoint). Secondary objectives were to assess immunogenicity and the durability of any reduction in PMR after a secondary blood-stage CHMI four months after the primary challenge.

The study was conducted in healthy volunteers aged 18 - 45 and was in two parts (Figures S1 and S6). The first part was a dose escalation / dose finding study (Phase Ia), examining the safety and immunogenicity of the vaccine (Groups 1-4, recruited and vaccinated at the CCVTM, Oxford; Guys and St Thomas’ NIHR CRF, London; and the NIHR WTCRF, Southampton). Participants in Groups 1, 2, and 4 received three vaccinations with RH5.1/AS01_B_ at days 0, 28 and 56, with a dose escalation of RH5.1 from 2 μg (Group 1), to 10 μg (Group 2), to 50 μg (Group 4), each administered in 0.5 mL AS01_B_. Participants in Group 3 received the same initial two 50 μg doses of RH5.1 as Group 4 (in 0.5mL AS01_B_) but then received a final fractional (one fifth) dose (10 μg RH5.1) given at day 182 (rather than 50 μg RH5.1 given at day 56 in Group 4). This delayed fractional dose (DFx) group was included due to reported improvements with the DFx regimen when using the pre-erythrocytic malaria vaccine RTS,S/AS01.^57^^,^^61^ Nominal study vaccination days are reported throughout; a window period of ± 7 days was permitted in the protocol. Volunteers in Groups 1-4 were enrolled between 17 October 2016 and 6 December 2017.

The second part of the study evaluated vaccine efficacy using a blood-stage CHMI methodology,^18^ taking forward the optimal vaccine dose (selected from Groups 1, 2 and 4 only) as determined by the Phase Ia. Volunteers for the Phase IIa (Groups 5-8) were recruited at the CCVTM, Oxford, and at Guy’s and St Thomas’ NIHR CRF, London and were enrolled between 4 September 2017 and 13 November 2017. Seventeen volunteers (Group 5) received three successive vaccinations with 10 μg RH5.1 in 0.5 mL AS01_B_ at 0, 28 and 56 days. Fourteen of these vaccinated volunteers went on to receive an intravenous blood-stage CHMI in parallel with fifteen unvaccinated malaria-naive infectivity control volunteers (Group 6), and any efficacy (assessed by a reduction in the PMR) was reported. This arm of the CHMI study was also termed “VAC063A.” Nine of these fourteen Group 5 vaccinees (Group 7) then went on to receive a fourth and final booster vaccination with RH5.1/AS01 _B_ (again at a dose of 10 μg RH5.1 in 0.5 mL AS01_B_) approximately four months after their third vaccination. Two weeks later they were administered a second homologous intravenous blood-stage challenge, in parallel with eight of the previous control volunteers (Group 8) and six new malaria-naive controls (Group 9, who were recruited only from Oxford and enrolled on 5 March 2018). This arm of the CHMI study was termed “VAC063B.” This second CHMI was to investigate the durability of any vaccine effect in parallel with any protective effect of a prior homologous challenge. All volunteers were challenged and followed up at the CCVTM, Oxford.

Allocation to study groups was sequential from Group 1 to Group 3. Groups 3 and 4 were then recruited simultaneously. Volunteers, where possible, were able to choose to which group they were allocated. For safety reasons the first volunteer who received a new vaccine dose was vaccinated alone and there was at least a 48 h gap before subsequent volunteers were vaccinated. A further two volunteers could be vaccinated 48 h after the first, and then at least another 48 h gap had to elapse before the remaining volunteers receiving that dose of vaccine could be vaccinated. Safety stopping and holding rules were used in this study to ensure participant safety, particularly given that this was a first-in-human dose escalation study, as detailed below.

*Stopping / Holding Rules.* Safety reviews took place prior to each dose escalation.

The following holding rules were applied to study Groups 1-5 and Group 7 (i.e., vaccinees). The study would have been put on hold if any of the following criteria were reached:

Solicited local adverse events:

If more than 25% of doses of a vaccine are followed by the same Grade 3 solicited local adverse event beginning within 2 days after vaccination (day of vaccination and one subsequent day) and persisting at Grade 3 for > 72 h.

Solicited systemic adverse events:

If more than 25% of doses of a vaccine are followed by the same Grade 3 solicited systemic adverse event beginning within 2 days after vaccination (day of vaccination and one subsequent day) and persisting at Grade 3 for > 48 h.

Unsolicited adverse events:

If more than 25% of volunteers develop the same Grade 3 unsolicited adverse event (including the same laboratory adverse event) that is considered possibly, probably or definitely related to vaccination and persists at Grade 3 for > 48 h.

A serious adverse event considered possibly, probably or definitely related to vaccination occurs.

Individual stopping rules (applied to all vaccinated individuals):

In addition to the above stated group holding rules, stopping rules for individual volunteers were applied (i.e., indications to withdraw individuals from further vaccinations). Volunteers would have been withdrawn from further vaccinations if any of the events listed below had occurred and were considered possibly, probably or definitely related to vaccination:

Local Reactions: Injection site ulceration, abscess or necrosis.
Laboratory AEs: The volunteer develops a Grade 3 laboratory adverse event considered possibly, probably or definitely related within 7 days after vaccination and persisting continuously at Grade 3 for > 72 h.
Solicited systemic adverse events: The volunteer develops a Grade 3 systemic solicited adverse event considered possibly, probably or definitely related within 2 days after vaccination (day of vaccination and one subsequent day) and persisting continuously at Grade 3 for > 72 h.
Unsolicited adverse events: The volunteer has a Grade 3 adverse event, considered possibly, probably or definitely related to vaccination, persisting continuously at Grade 3 for > 72 h.
The volunteer has a serious adverse event considered possibly, probably or definitely related to vaccination.
The volunteer has an acute allergic reaction or anaphylactic shock following the administration of the vaccine investigational product.

Safety reviews were carried out by the Local Safety Monitor (LSM) prior to each dose escalation (after the first six vaccinations in each of the dose groups), and no concerns were raised with any of the vaccine doses.

*Monitoring.* The LSM provided safety oversight, and Good Clinical Practice (GCP) compliance was independently monitored by the University of Oxford Clinical Trials and Research Governance (CTRG) Office.

*Inclusion and Exclusion Criteria.* A medical history and physical examination were conducted at the screening visit, as well as baseline blood tests including a full blood count; urea and electrolytes; liver function tests; and hepatitis B virus (HBV), hepatitis C virus (HCV) and human immunodeficiency virus (HIV) serology. Dipstick urinalysis for all volunteers and pregnancy testing for all female volunteers were conducted at screening, as well as an electrocardiogram for all volunteers due to undergo CHMI (Groups 5-9). Pregnancy testing was also carried out in female volunteers prior to each vaccination, prior to CHMI and prior to initiation of antimalarial therapy. A full list of inclusion and exclusion criteria is shown below:

*—Inclusion criteria.* Volunteers had to satisfy all the following criteria to be eligible for the study:

Healthy adults aged 18 to 45 years.
Able and willing (in the Investigator’s opinion) to comply with all study requirements.
Willing to allow the Investigators to discuss the volunteer’s medical history with their General Practitioner (GP).
For females only, willingness to practice continuous effective contraception (see below) during the study and a negative pregnancy test on the day(s) of screening and vaccination, and on the day prior to blood-stage CHMI, and prior to the start of antimalarial treatment for Groups 5-9 volunteers.
Agreement to refrain from blood donation during the course of the study.

Provide written informed consent.

*—Additional Inclusion Criteria for Groups 5 - 9.* Agreement to permanently refrain from blood donation, as per current UK Blood Transfusion and Tissue Transplantation Services guidelines.^62^

Reachable (24 hours a day) by mobile phone during the period between CHMI and completion of antimalarial treatment.
Willingness to take a curative anti-malaria regimen following CHMI.
Answer all questions on the informed consent questionnaire correctly.
For Groups 7-9: completion of primary challenge, curative anti-malarials and follow-up (up until at least the dC+28 visit)

*—Exclusion Criteria.* Volunteers were not eligible to participate if any of the following applied:

Participation in another research study involving receipt of an investigational product in the 30 days preceding enrolment, or planned use during the study period.
Prior receipt of an investigational vaccine likely to impact on interpretation of the trial data, as assessed by the investigator. For Group 7 volunteers undergoing re-challenge, this exclusion criterion does not extend to the RH5.1/AS01_B_ vaccine previously received.
Any medical condition that in the judgment of the Investigator would make intramuscular (IM) injection unsafe.
Administration of immunoglobulins and/or any blood products within the three months preceding the planned administration of the vaccine candidate.
Any confirmed or suspected immunosuppressive or immunodeficient state, including HIV infection; asplenia; recurrent, severe infections and chronic (more than 14 days) immunosuppressant medication during the period starting six months prior to the first vaccine dose. For corticosteroids, this will mean prednisone 20 mg/day (for adult subjects), or equivalent. Inhaled and topical steroids are allowed.
Administration of long-acting immune-modifying drugs at any time during the study period (e.g., infliximab).
Chronic use of antibiotics with antimalarial effects (e.g., tetracyclines for dermatologic patients, sulfa for recurrent urinary tract infections, etc.).
History of malaria chemoprophylaxis within 60 days prior to vaccination.
History of allergic disease or reactions likely to be exacerbated by any component of the vaccine.
Any history of anaphylaxis in relation to vaccination.
Pregnancy, lactation or willingness/intention to become pregnant during the study.
History of cancer (except basal cell carcinoma of the skin and cervical carcinoma *in situ*).
History of serious psychiatric condition likely to affect participation in the study.
Any other serious chronic illness requiring hospital specialist supervision.
Suspected or known current alcohol abuse as defined by an alcohol intake of greater than 42 units every week.
Suspected or known injecting drug abuse in the 5 years preceding enrolment.
Seropositive for hepatitis B surface antigen (HBsAg).
Seropositive for hepatitis C virus (antibodies to HCV) at screening (*unless* has taken part in a prior hepatitis C vaccine study with confirmed negative HCV antibodies prior to participation in that study, and negative HCV RNA PCR at screening for this study).
History of clinical malaria (any species; not applicable to prior CHMI for Groups 7, 8 and 9).
Travel to a malaria endemic region during the study period or within the previous six months.
Any clinically significant abnormal finding on screening biochemistry or hematology blood tests or urinalysis.
Any other significant disease, disorder or finding which may significantly increase the risk to the volunteer because of participation in the study, affect the ability of the volunteer to participate in the study or impair interpretation of the study data.
Inability of the study team to contact the volunteer’s GP to confirm medical history and safety to participate.

*—Additional Exclusion Criteria for Groups 5-9.* Use of systemic antibiotics with known antimalarial activity within 30 days of CHMI (e.g., trimethoprim-sulfamethoxazole, doxycycline, tetracycline, clindamycin, erythromycin, fluoroquinolones and azithromycin).

History of sickle cell anemia, sickle cell trait, thalassaemia or thalassaemia trait or any hematological condition that could affect susceptibility to malaria infection.
Laboratory evidence of glucose-6-phosphate dehydrogenase (G6PD) deficiency at screening.
Laboratory evidence of haemoglobinopathy at screening.
Use of medications known to cause prolongation of the QT interval *and* existing contraindication to the use of Malarone.
Use of medications known to have a potentially clinically significant interaction with Riamet *and* Malarone.
Contraindications to the use of both Riamet *and* Malarone.
Any clinical condition known to prolong the QT interval.
Family history of congenital QT prolongation or sudden death.
Positive family history in both 1st and 2nd degree relatives < 50 years old for cardiac disease.
History of cardiac arrhythmia, including clinically relevant bradycardia.
Volunteer unable to be closely followed for social, geographic or psychological reasons.

*Safety Analysis.* Following each vaccination, volunteers completed an electronic diary card for 28 days with any AE data. Participants were asked to record both solicited and unsolicited AEs. Data on solicited AEs were collected for 7 days after each vaccination, whereas details of any unsolicited AEs were collected for the duration of each diary. Solicited AEs were those expected following an intramuscular vaccination and included local AEs (pain, erythema, warmth, swelling and itching) and systemic AEs (headache, malaise, myalgia, arthralgia, feverishness, nausea, fatigue, and measured fever). If these AEs occurred outside of the first seven days they were defined as unsolicited. Any solicited AEs occurring during the diary card period were defined as being at least possibly related to vaccination.

Volunteers graded all AEs as mild, moderate or severe:

GRADE 0: None.
GRADE 1: Transient or mild discomfort (< 48 h); no medical intervention/therapy required.
GRADE 2: Mild to moderate limitation in activity – some assistance may be needed; no or minimal medical intervention/therapy required.
GRADE 3: Marked limitation in activity, some assistance usually required; medical intervention/therapy required; hospitalization possible.

AE data also included the results of the hematology (full blood count) and biochemistry (liver function tests, urea and electrolytes) carried out at all visits during the diary card period, except at 2 days post-vaccination, when blood was not taken.

For each unsolicited AE, an assessment of the relationship of the AE to the study intervention(s) was undertaken. Alternative causes of the AE, such as the natural history of pre-existing medical conditions, concomitant therapy, other risk factors and the temporal relationship of the event to vaccination were considered. The likely causality of all unsolicited AEs was assessed as per the criteria below:

No Relationship: No temporal relationship to study product *and* alternate etiology (clinical state, environmental or other interventions); *and* does not follow known pattern of response to study product.
Unlikely: Unlikely temporal relationship to study product *and* alternate etiology likely (clinical state, environmental or other interventions) *and* does not follow known typical or plausible pattern of response to study product.
Possible: Reasonable temporal relationship to study product; *or* event not readily produced by clinical state, environmental or other interventions; *or* similar pattern of response to that seen with other vaccines.
Probable: Reasonable temporal relationship to study product; *and* event not readily produced by clinical state, environment, or other interventions *or* known pattern of response seen with other vaccines.
Definite: Reasonable temporal relationship to study product; *and* event not readily produced by clinical state, environment, or other interventions; *and* known pattern of response seen with other vaccines.

All unsolicited AEs that were assessed as being possibly, probably or definitely related to RH5.1/AS01_B_ are shown in Data S1B.

Data on AEs of special interest were also collected for Groups 1-5 and Group 7 (vaccinees) and data on serious adverse events (SAEs) were collected for all groups throughout the study period.

*—AEs in the post-CHMI period.* Post-CHMI, volunteers were asked if they had experienced solicited malaria symptoms, and asked to grade these (as above). Data on AEs occurring during and after the CHMI period (that may have related to CHMI or antimalarial treatment) were collected at clinic visits, from dC+1 up until 90 days post-CHMI. These CHMI-related safety data will be fully reported elsewhere (Y.T., unpublished data).

*Follow-up Schedule.* Vaccination visits occurred on days 0, 28 and 56 (Groups 1, 2, 4 and 5) and days 0, 28, 182 (Group 3). Group 7 received a fourth and final vaccination 98 days (fourteen weeks) post-CHMI (and 112 days, sixteen weeks post-third vaccination).

Reviews post-vaccination for Groups 1, 2 and 4 occurred on Days 1, 7, 14, 29, 35, 42, 57, 63, 70, 84, 140 and 240. Reviews for Group 3 were on Days 1, 7, 14, 29, 35, 42, 56, 183, 189, 196, 210, 266 and 366. Reviews for Group 5 were as per Groups 1, 2 and 4, until day 63. They then attended on day 69 – the day before challenge (dC-1 visit) – and underwent CHMI fourteen days post-final vaccination. Reviews post-vaccination for Group 7 – i.e., after the final and fourth vaccination – were as for Group 5, including a day 1 and day 7 visit post-vaccination, with a dC-1 visit and CHMI 14 days after the final vaccination. Volunteers in Groups 5-9 were reviewed in clinic the day after challenge (dC+1), twice a day from dC+2 until dC+12, then once daily from dC+13 (if not yet diagnosed) until dC+21 (or diagnosis). Upon malaria diagnosis or dC+21 being reached, subjects were reviewed approximately 24 and 48 h after diagnosis and / or starting antimalarial therapy, then at dC+28 and finally at dC+90. Group 5 additionally attended for a dC+170 visit.

Volunteers were invited back for a final “late bleed” approximately 1.5-2.5 years after the first vaccination in order to assess the longevity of the vaccine-induced response. Thirty of the vaccinated volunteers in Groups 1-4 (at day 875, 663 and 626 (median) for Groups 1, 2 and 4 respectively, and at day 687 (median) in Group 3) returned for a late bleed visit. Additionally, three of the volunteers in Group 5 (at day 643, median) and six in Group 7 (at day 643, median) underwent a late bleed.

*Vaccine Safety – Extra Information.* Solicited adverse events (AEs) in Groups 1-4, 5 and 7 were primarily mild to moderate in severity; however, occasional severe AEs occurred (Figures S2 and S7). The majority of these solicited AEs lasted for approximately 24-48 hours following vaccination. The unsolicited AEs considered possibly, probably or definitely related to the vaccine are shown in Data S1B. The majority were mild in nature and all resolved spontaneously. One Group 5 volunteer developed mild bilateral infraorbital swelling within 24 h of their second vaccination. They had no other features of an allergic reaction, required no clinical intervention, and the swelling had completely resolved within 48 hours. The volunteer was, however, withdrawn from the study due to the possibility that this represented a mild allergic reaction.

A single occurrence of scalp psoriasis in one Group 3 volunteer was retrospectively regraded a suspected unexpected serious adverse reaction (SUSAR) on the basis of i) its likely autoimmune nature (all new/suspected autoimmune events were collected as AEs of special interest and therefore reported as SAEs, as for previous clinical trials of vaccines using the AS01_B_ adjuvant); and ii) its potentially plausible time of onset in relation to vaccination (onset 1 month post-second vaccination). This made a causal relationship “possible,” despite being unlikely clinically.

Four grade 3 laboratory AEs were reported in vaccinated participants (prior to CHMI) (Data S1A): A grade 3 lymphocytopenia occurred in one volunteer in Group 1 occurring on day 6 post-first vaccination. This was not felt to be related to vaccination as the volunteer became unwell with malaise, nausea, muscle aches, feverishness and diarrhea starting *de novo* 6 days post-vaccination, having been completely well prior. The symptoms and lymphocytopenia resolved spontaneously within 72 hours. A grade 3 hyperkalaemia occurred in one Group 1 volunteer 7 days post-third vaccination, but was reported by the testing laboratory as a haemolysed sample and had normalized on repeat testing 72 hours later. A grade 3 hypoalbuminemia (9 g/dL) was reported in one Group 5 volunteer at day14 post-first vaccination. However, this was reported by the lab as a spurious result, and was within the normal range when re-checked 2 days later. Finally, a grade 3 anemia (Hb 88 g/dL) occurred in one Group 4 volunteer at days 7 and 14 post-first vaccination. However, this was subsequently found to be a preceding condition for which the volunteer was under investigation and treatment (not initially apparent at screening), and so the volunteer was withdrawn from the study.

One volunteer in Group 7 fell pregnant following her fourth 10 μg dose of the vaccine and her second blood-stage CHMI (pregnancy testing by urinary β-HCG prior to challenge was negative). Pregnancy was first detected by urinary β-HCG testing on day 9 post-challenge, following routine testing at time-point of malaria diagnosis, just prior to treatment. Following immediate discussion between the Chief Investigator and the Local Safety Monitor, this volunteer was treated with Riamet. Pregnancy was also confirmed within 24 hours by serum β-HCG testing. The volunteer was closely monitored over the treatment phase and afterward with no further clinical concerns arising. There was no detectable malaria DNA by qPCR after 3 weeks. She remained well and went on to have a termination of pregnancy about 2 months later for personal reasons.

*Blood-Stage Inoculum and CHMI.* The inoculum used for CHMI was produced at QIMR Berghofer Medical Research Institute in Brisbane, Australia in 1994 and consists of aliquots of *P. falciparum* (clone 3D7) infected erythrocytes taken from a single donor.^60^^,^^63^^,^^64^ Over 400 volunteers have been challenged with the inoculum since 1997 (79 in Oxford inclusive of the VAC063 trial), and the estimated number of infected erythrocytes has varied from 30 to 6000. CHMI of malaria-naive individuals using this inoculum has always resulted in parasitemia as detected by qPCR and/or microscopy.^63^^,^^64^ All volunteers within VAC063A and within VAC063B were challenged with the same preparation of the inoculum. The intended inoculum was 1000 infected erythrocytes per volunteer, thawed and prepared under strict aseptic conditions as previously described,^18^ with some modifications. Briefly, a single vial of cryopreserved erythrocytes was thawed in a derogated containment level III laboratory area using solutions licensed for clinical use and single-use disposable consumables. A class II microbiological safety cabinet (MSC) was used to prepare the inoculum, which was fumigated with hydrogen peroxide and decontamination validated prior to use. To prepare the inoculum, 0.2 volume 12% saline was added dropwise to ∼1.5 mL of thawed infected blood, left for 5 min, and an additional 10 volumes of 1.6% saline added dropwise. This was centrifuged for 4 min at 830 x*g*, the supernatant was removed, and 10 mL of 0.9% saline was added dropwise. The cell pellet was washed twice in 0.9% saline and resuspended in 0.9% saline in a sterile syringe for injection. The injection volume per volunteer was 5 mL containing an estimated 1000 parasitized erythrocytes based on microscopic estimates of the donor’s parasite density prior to freezing. The clinical inoculum was also cultured following preparation and shown to be negative for bacterial contamination. The order in which vaccinated and unvaccinated volunteers received the inoculum was interspersed in case of time effects on viability of the parasites. All subjects were inoculated intravenously, in a total volume of 5 mL of normal saline followed by a saline flush, within 2 h 6 min of inoculum preparation (VAC063A) and 2 h 8 min of inoculum preparation (VAC063B). Subjects were observed for 1 h before discharge from the clinical facility.

*Diagnostic Criteria.* For VAC063A (Groups 5 and 6), at each time-point thick blood films were evaluated by experienced microscopists and qPCR was performed in real time. Diagnosis of malaria required volunteers to fulfil two of three criteria: a positive thick blood film (one viable parasite in 200 fields) and/or qPCR ≥ 500 parasites/mL (p/mL) and/or symptoms consistent with malaria infection. However, in VAC063B (Groups 7-9) microscopy was removed as a diagnostic tool. This reduced the number of volunteers diagnosed prematurely (i.e., < 5,000 p/mL) in VAC063B (Figure S8E) without impacting on volunteer safety. In light of the above, the new criteria for diagnosis and immediate treatment of volunteers in VAC063B (Groups 7-9), were:

Asymptomatic with any available qPCR result ≥ 10,000 parasites/mL;
Symptomatic with any available qPCR result ≥ 5,000 parasites/mL.

At diagnosis volunteers were treated with Riamet (except for 4 volunteers who received Malarone due to documented sinus bradycardia pre-challenge, and one who received Malarone due to a potential concomitant medication interaction with Riamet). Follow-up visits took place at days dC+28 and dC+90.

### Method details

#### Inoculum Viability

Parasite viability was assayed by limiting dilution assay, as described previously,^18^ and was setup at the time the last volunteer was infected. In brief, the culture period was 10 days and after this time qPCR was used to score wells positive or negative for replicating parasites. Because the qPCR assay can also detect dead parasites, a plate of identical dilutions of the inoculum that had been frozen without incubation was used as a negative control; there was no detectable amplification from these wells, and unincubated control wells which had received a 100-fold greater parasite inoculum gave positive results. Cultured wells plated at an estimated 1.5 parasites/well gave a clear bimodal distribution, with wells showing negative results by qPCR (suggesting they contained no viable parasite), and wells that gave highly positive results by qPCR (suggesting they had contained at least one viable parasite at the start of the culture period). The number of viable parasites/mL of inoculum could then be calculated with reference to the Poisson distribution, and viability expressed as a percentage of the pre-freezing microscopy-estimated parasitemia calculated using the RBC count/mL of inoculum. For VAC063A, the limiting dilution assay demonstrated 45.2% viability (i.e., an effective inoculum of 452 parasites per volunteer), and for VAC063B this was 85.7% viability (i.e., 857 parasites per volunteer).

#### Parasite qPCR

qPCR was conducted as previously described,^18^ with the following modifications. Briefly, blood was collected at baseline and at clinical protocol defined time-points following CHMI for qPCR in 2.0 mL tubes containing EDTA. DNA was extracted from 0.4 mL EDTA whole blood using a QIAsymphony SP robot, utilizing the QIAGEN DSP Blood Midi Kit and the pre-loaded Blood 400 v6 extraction protocol, with a 100 μL elution in ATE buffer selected. 5 μL of each extraction was used per assay well and was run in triplicate for qPCR (equivalent to 60 μL blood directly assessed). Parasites per mL (p/mL) equivalent mean values were generated by a standard Taqman absolute quantitation, against a defined standard curve of diluted *P. falciparum* 3D7 DNA, qualified against DNA from counted parasites in whole blood, previously extracted by the same method. qPCR was conducted on an ABI StepOne Plus machine with v2.3 software, using default Universal qPCR and QC settings, apart from the use of 45 cycles and 25 μL reaction volume. This process has since been formally validated as suitable for diagnostic purposes and qPCR detection is regularly externally assessed by participation in the UKNEQAS Malaria (Molecular) scheme.

Based upon results obtained using dilution series of microscopically-counted cultured parasites, this method has a lower limit of quantification (LLQ, defined as %CV < 20%) of around 20 p/mL blood.^27^ Counted parasite dilution series results suggest that the lower limit of probable detection (LLD, i.e., a probability of > 50% of ≥ 1 positive result among three replicate qPCR reactions) is in the region of 5 p/mL, while samples at 1 p/mL are consistently negative (24/24 qPCR reactions). Positive results in this assay (even at very low level) are thus essentially 100% specific for genuine parasitemia, with positive results beneath the LLQ likely to signify parasitemia in the range 2-20 p/mL.

For quality control purposes, qPCR samples were re-tested if;

Replicates included a mixture of positive and negative (in terms of amplification) results with one or more positive results > 100 p/mL.
The % CV of any results were high outliers.

All ‘passed’ data following quality control QC steps above, including any 0 values, were used to generate the mean result for each time-point.

#### Peripheral Blood Mononuclear Cell (PBMC), Plasma and Serum Preparation

Blood samples were collected into lithium heparin-treated vacutainer blood collection systems (Becton Dickinson, UK). PBMC were isolated and used within 6 hours in fresh assays as previously described.^65^ Excess cells were frozen in fetal calf serum (FCS) containing 10% dimethyl sulfoxide (DMSO) and stored in liquid nitrogen. Plasma samples were stored at −80°C. For serum preparation, untreated blood samples were stored at room temperature (RT) and then the clotted blood was centrifuged for 5 min (1000 *xg*). Serum was stored at −80°C.

#### Peptides

Peptides for *ex-vivo* IFN-γ ELISPOT were purchased from NEO Scientific (Cambridge, MA, USA). Sequences have been reported previously,^11^ although eight of these were replaced with slight sequence changes to provide exact match to the RH5.1 vaccine. In brief, the peptides (20 amino acids (aa) in length and overlapping by 10 aa) covered the entire RH5 sequence present in the RH5.1 protein vaccine. Peptides were reconstituted in 100% DMSO at 50-200 mg/mL and combined into various pools for the ELISPOT assay.

#### Recombinant RH5 Protein

Recombinant RH5.1 protein was used for all ELISA assays, B and T cell ELISPOT assays and flow cytometry assays. The protein was produced and purified from a stably transfected *Drosophila* S2 cell line as previously described.^11^^,^^21^

#### Ex-vivo IFN-γ ELISPOT

Fresh PBMC were used in all assays using a previously described protocol.^11^ Spots were counted using an ELISPOT counter (Autoimmun Diagnostika (AID), Germany). Results are expressed as IFN-γ spot-forming units (SFU) per million PBMC. Background responses in unstimulated control wells were almost always less than 20 spots, and were subtracted from those measured in peptide-stimulated wells.

#### Flow Cytometry

RH5.1-specific CD4^+^, CD8^+^ and CD4^+^ Tfh cells were analyzed with an activation induced marker (AIM) assay as previously described,^66^ with the exception of stimulation with RH5.1 protein rather than a peptide pool, and the addition of brefeldin A (BFA; 00-4506-51, Life Technologies). Briefly, cryopreserved PBMC were thawed and rested before stimulation for 24 h with medium alone, 1 μg/mL RH5.1, or 1 μg/mL *Staphylococcal* enterotoxin B (SEB; S-4881, Sigma; positive control). BFA was included for the final 2 h of stimulation at 3 μg/mL. Medium only served as a negative control. Anti-human CD183-APC (550967, clone: 1C6/CXCR3; BD Biosciences) was included in the cell culture medium. Following incubation, PBMC were stained and fixed with Cytofix/Cytoperm (554714, BD Biosciences). The following anti-human antibodies / dyes were used: anti-PD1-BV421 (329920, clone: EH12.2H7), anti-CD14-BV510 (301842, clone: M5E2), anti-CCR6-BV711 (353436, clone: G034E3), anti-CD3-BV605 (300460, clone: UCHT1), anti-CD137-BV650 (309828, clone: 4B4-1), and anti-CD45RO-BV785 (304234, clone: UCHL1) – all Biolegend; anti-CD19-BV510 (562947, clone: SJ25C1), anti-CD69-PE-Cy5 (555532, clone: FN50), anti-CXCR5-AF700 (565191, clone: RF8B2), anti-CD4-APC-H7 (641398, clone: SK3), anti-OX40-PE (340420, clone: L106), anti-CD25-PE-Cy7 (335824, clone: 2A3), and streptavidin-BB515 (564453) – all BD Biosciences); anti-IFN-γ-PerCP-Cy5.5 (45-7319-42, clone: 42.B3), and anti-CD8-PE-TR (MHCD 0817, clone: 3B5) – both Life Technologies; Live/Dead Aqua (L34966), and anti-ICOS-biotin (13-9948-82, clone: ISA-3) – both Invitrogen. Samples were acquired on a Fortessa flow cytometer using BD FACSDiva (both BD Biosciences) and data were analyzed in FlowJo (v10, Treestar). For the Tfh cell analysis, RH5.1-specific cells were defined using Boolean gating as cells co-expressing CD25 with OX40 and/or CD137 and/or CD69 following stimulation with RH5.1. Frequencies of total CD4^+^ and CD8^+^ T cells producing IFN-γ in response to RH5.1 stimulation were measured in the same assay. For both Tfh cells and IFN-γ-producing T cells, the frequency of activated cells in sample-matched unstimulated wells was subtracted to control for non-specific activation. Samples were excluded from analysis if the parent population contained < 50 cells.

#### Total IgG ELISAs

ELISAs were performed against full-length RH5 protein (RH5.1) using standardized methodology as previously described.^11^ The reciprocal of the test sample dilution giving an optical density at 405nm (OD_405_) of 1.0 in the standardized assay was used to assign an ELISA unit value of the standard. A standard curve and Gen5 ELISA software v3.04 (BioTek, UK) was used to convert the OD_405_ of individual test samples into arbitrary units (AU). These responses in AU are reported in μg/mL following generation of a conversion factor by calibration-free concentration analysis (CFCA) as reported previously.^11^

#### Avidity and Isotype/Subclass ELISAs

Anti-RH5.1 IgG antibody avidity was assessed by sodium thiocyanate (NaSCN)-displacement ELISA using previously described methodology.^11^ The concentration of NaSCN required to reduce the OD_405_ to 50% of that without NaSCN was used as a measure of avidity (IC_50_). Anti-RH5.1 antibody isotype and subclass ELISAs were also performed using methodology described in detail elsewhere.^11^

#### Antibody-Secreting Cell (ASC) ELISPOT

*Ex-vivo* ASC ELISPOT assays were performed against RH5.1 protein as described in detail elsewhere^11^ using fresh PBMC. Plates were counted using an AID ELISPOT plate reader. Results are reported as RH5_FL-specific ASC as a % of the total number of measured IgG-secreting B cells.

#### Serum IgG Concentration

Total serum IgG concentrations were determined using a Bio-Monolith Protein G column on an Agilent 1260 HPLC system (Agilent, Cheshire, UK). Separation was performed at 1 mL/min using PBS and 0.2 M Glycine pH 2.0 as mobile phases with detection at UV 280 nm. A calibration curve was produced using purified human IgG.

#### Assay of Growth Inhibition Activity (GIA)

Standardized assays were performed by the GIA Reference Center, NIH, USA, using previously described methodology,^67^ with one modification. Here, each sample was tested in three independent replication assays, and the median of these three results was used to generate the final dataset. Otherwise for each assay, in brief, protein G purified IgG samples were incubated with red blood cells (RBC) infected with synchronized *P. falciparum* 3D7 clone parasites in a final volume of 40 μL for 40 h at 37°C, and the final parasitemia in each well was quantified by biochemical determination of parasite lactate dehydrogenase. All samples were tested at 10mg/mL in a final test well, followed by a dilution series for positive samples to determine the concentration that gave 50% GIA (EC_50_).

#### Viral-Vectored RH5 Trial Data

Where specifically referred to in the text, historical data were included for comparison from the VAC057 Phase Ia clinical trial of a viral-vectored RH5 (VV-RH5) vaccine.^11^ RH5-specific immune readouts were measured in an identical manner across the different clinical trials. Serology/GIA (day 84) and T cell ELISPOT (day 63) assay data for the VV-RH5 vaccine are shown from Group 2C in that trial.

#### FMP2.1 AMA1/AS01 Trial Data

Where specifically referred to in the text, historical data were included for comparison from the VAC054 Phase I/IIa clinical trial of the FMP2.1 AMA1/AS01 vaccine.^18^ PMR efficacy and GIA assay readouts were measured in an identical manner across the different clinical trials.

#### Systems Serology Analyses

Experimenters at Massachusetts General Hospital (MGH) were blinded as to the sample identity until all data had been collected. Assays performed at MGH using plasma samples from the VAC063 trial were deemed not human research following review by the MGH Institutional Review Board (protocol 2012P002452). Additionally, human whole blood and buffy coats were collected at MGH from healthy donors who did not participate in the VAC063 trial. Use of these internal samples as sources of uninfected primary neutrophils and NK cells was deemed not human research by the MGH IRB (protocols 2010P002121 and 2005P001218).

#### Fluorescent Primary and Secondary Antibodies

The following fluorescent antibodies were purchased from BD Biosciences: APC-Cy7 anti-huCD14 (#557831), PE-Cy7 anti-huCD3 (#563423), PE-Cy7 anti-huCD56 (#335791), and BV421 anti-huMIP-1β (#562900). Additional fluorescent antibodies were purchased from BioLegend: Pacific Blue anti-huCD66b (#305112), APC-Cy7 anti-huCD3 (#300426), BV605 anti-huCD107a (#328634), and PE anti-huIFN-γ (#506507). A FITC-conjugated, goat anti-guinea pig complement C3 polyclonal antibody was purchased from MP Biomedical (#0855385). PE-conjugated secondary antibodies were purchased from Southern Biotech for the detection of total huIgG (#9040-09), huIgM (#9020-09), huIgA1 (#9130-09), huIgA2 (#9140-09), huIgG1 (#9052-09), huIgG2 (#9070-09), huIgG3 (#9210-09), and huIgG4 (#9200-09).

#### Antigen Coupling to Fluorescent Beads

NeutrAvidin-labeled yellow-green (#F8776) and red (#F8775) fluorescent 1 μm microspheres were purchased from Thermo Fisher Scientific. For immune functional assays, 1.8 × 10^8^ NeutrAvidin-labeled fluorescent microspheres were coupled to 5 μg biotinylated PfRH5 antigen (exact construct described in Nielsen et al.^66^) by co-incubation in PBS/5% BSA (PBSA) overnight at 4°C, then the beads were washed twice with PBSA. Magplex-C microspheres (Luminex Corp) were covalently coupled to streptavidin (Jackson Immunoresearch, #016-000-113) using a two-step carbodiimide reaction. Magplex-C beads (9 × 10^8^) were washed, resuspended in 100 mM NaH_2_PO_4_ (pH 6.2), and activated by incubating with 500 μg Sulfo-NHS (Pierce, #A39269) and 500 μg EDC (Pierce, #A35391) for 30 min at room temperature (RT). The beads were washed three times with coupling buffer (50 mM MES, pH 5.0), then incubated with streptavidin in 500 μL of coupling buffer for 2 h at RT. The beads were washed with PBS/0.05% Tween-20, incubated overnight at 4°C with 100 μg/mL biotinylated PfRH5 in PBSA, washed, and stored in PBS/0.05% sodium azide.

#### THP-1 Monocyte Phagocytosis Assay

An assay for measuring antibody-dependent THP-1 monocyte / cellular phagocytosis (ADCP) was used as previously described.^68^ Briefly, 1 μm yellow-green fluorescent NeutrAvidin beads were coupled to biotinylated PfRH5 antigen and blocked overnight with PBSA. The beads were then washed twice with PBSA, diluted to 1.8 × 10^8^ beads/mL, and 10 μL beads/well were added to a 96-well round-bottom microplate. Diluted plasma from immunized subjects (10 μL/well) was added to the beads and incubated at 37°C for 2 h to allow the formation of immune complexes. Unbound antibodies were washed off, then 25,000 THP-1 cells/well (ATCC, #TIB-202) were added to the beads in 200 μL THP-1 medium (R-10 + 55 μM β-ME) and incubated overnight at 37°C. Cells were fixed and acquired on an Intellicyt iQue Screener PLUS flow cytometer. The phagocytic score for each sample was calculated as (% bead-positive cells) x (gMFI of bead-positive cells)/(10 x gMFI of first bead-positive peak).

#### Primary Neutrophil Phagocytosis Assay

An assay for measuring antibody-dependent neutrophil phagocytosis (ADNP) has been described previously.^69^ Briefly, 1 μm yellow-green fluorescent NeutrAvidin beads were coupled to biotinylated PfRH5 antigen and blocked with PBSA overnight at 4°C. The beads were then washed twice with PBSA and diluted to 1.8 × 10^8^ beads/mL. PfRH5-coupled beads (10 μL/well) and diluted test plasma (10 μL/well) were combined in a round-bottom 96-well plate, then incubated at 37°C for 2 h. Primary leukocytes were isolated from freshly drawn whole blood (collected from healthy donors in anticoagulant citrate dextrose tubes) by treatment with ACK red blood cell lysis buffer, then diluted in R-10 media to 250,000 cells/mL. After immune complex formation, the beads were washed, combined with 50,000 primary leukocytes/well, and incubated for 1 h at 37°C. Cells were stained for surface CD66b, CD14, and CD3, fixed, and acquired on an Intellicyt iQue Screener PLUS flow cytometer. Gates were drawn to identify singlet SSC^high^ CD66b^+^ CD14^-^ CD3^-^ cells, and phagocytic scores for each sample were calculated as (% bead-positive cells) x (gMFI of bead-positive cells)/(10 x gMFI of the first bead-positive peak).

#### Complement Deposition Assay

An assay for measuring antibody-dependent complement deposition (ADCD) was used as previously described.^70^ Briefly, 1 μm red fluorescent NeutrAvidin beads were incubated with biotinylated PfRH5 antigen, blocked with PBSA, then washed and diluted to 1.8 × 10^8^ beads/mL. PfRH5-coupled beads (10 μL/well) were combined with diluted test plasma (10 μL/well) in a 96-well round-bottom microplate, then incubated at 37°C for 2 h. Guinea pig complement (CedarLane, #CL4051) was diluted in gelatin veronal buffer containing calcium and magnesium (GVB++, Boston Bioproducts, #IBB-300). The beads were washed with PBS and incubated with diluted complement for 20 min at 37°C. The beads were then washed with 5 mM EDTA, stained with FITC-conjugated anti-complement C3, and acquired on an Intellicyt iQue Screener PLUS flow cytometer. Gates were drawn on singlet, red fluorescent particles, and complement deposition was reported as the median fluorescence intensity (MFI) on the FITC channel.

#### NK cell Activation Assay

An assay for measuring antibody-dependent NK cell activation (ADNKA) has been described previously.^71^^,^^72^ Flat-bottom 96-well ELISA plates (Thermo Fisher, #439454) were coated with biotinylated PfRH5 antigen, then blocked with PBSA. Plasma samples from test subjects were diluted in PBSA, added to the plates, and incubated for 2 h at 37°C. Primary human NK cells were purified from buffy coats from healthy donors using the RosetteSep human NK cell enrichment cocktail (StemCell, #15065), then resuspended in R-10 media containing 10 μg/mL brefeldin A (Sigma, #B7651), GolgiStop (BD Biosciences, #554724), and fluorescent anti-CD107a. The ELISA plates were washed three times with PBS, then isolated NK cells (25,000/well) were added and incubated at 37°C for 5 h. The cells were then stained for surface CD56 and CD3, permeabilized, stained with fluorescent antibodies to IFN-γ and MIP-1β, fixed, and acquired on an Intellicyt iQue Screener PLUS flow cytometer. Gates were drawn on singlet, CD56^+^/CD3^-^ cells, and results were reported as the percentages of these cells that expressed surface CD107a, intracellular MIP-1β, or intracellular IFN-γ.

#### Antibody Isotype and Subclass Analysis

The isotypes and subclasses of PfRH5 antigen-specific antibodies were quantified using a previously described method.^73^ Magplex-C microspheres were coupled to streptavidin via carbodiimide crosslinking with Sulfo-NHS and EDC. Streptavidin-coupled beads were then incubated overnight with biotinylated PfRH5 antigen, blocked with PBSA, and added to black flat-bottom 384-well plates (Greiner Bio-One, #781906) so that each well contained 1500 RH5-coupled beads. Plasma from test subjects was diluted in PBSA and co-incubated with the beads for 2 h at RT on a plate shaker (800 rpm). The beads were then washed and incubated with a PE-conjugated antibody to detect total human IgG, huIgG1, huIgG2, huIgG3, huIgG4, huIgM, huIgA1, or huIgA2 for 1 h at RT on a plate shaker (800 rpm). The beads were then washed and acquired on an Intellicyt iQue Screener PLUS flow cytometer. Results were reported as the median PE fluorescence intensity.

#### Fc-binding Protein Array

The binding of PfRH5 antigen-specific antibodies to human Fc receptors (FcR) and complement C1q was measured using a previously-described assay.^74^^,^^75^ Briefly, avi-tagged FCGR2A, FCGR2B, FCGR3A, and FCGR3B proteins were produced and purified by the Duke Human Vaccine Institute Protein Production Facility. These proteins were then biotinylated with BirA ligase using a commercially available kit (Avidity, #BirA500). Purified human C1q protein (Sigma, #C1740) was biotinylated using EZ-Link Sulfo-NHS-LC-LC-Biotin (Pierce, #A35358) according to the manufacturer’s instructions. These biotinylated Fc domain-binding proteins were then incubated with streptavidin-PE (Prozyme, #PJ31S) to generate the assay detection reagents. Magplex-C microspheres were coupled to biotinylated PfRH5 antigen as described above, blocked with PBSA, and added to 384-well plates so that each well contained 1500 RH5-coupled beads. Plasma from test subjects was diluted in PBSA, added to the beads, and incubated for 2 h at RT on a plate shaker (800 rpm). The beads were then washed, incubated with one of the PE/FcR conjugates for 1 h at RT on a plate shaker (800 rpm), washed again, and acquired on an Intellicyt iQue Screener PLUS flow cytometer. Results were reported as the median PE fluorescence intensity.

#### Fc glycan Analysis

The Fc glycans on RH5 antigen-specific IgG were analyzed using a previously described method.^76^^,^^77^ Briefly, 200 μL of plasma from each vaccinated subject was heat-inactivated at 56°C for 1 h, then centrifuged at 20,000 x*g* for 10 min at RT. The resulting supernatant samples were first pre-cleared by incubating with 1 μm magnetic streptavidin-coated microspheres (New England Biolabs, #S1420S) for 1 h at RT. A magnet was used to pellet the beads, and the supernatants were then transferred to new tubes containing streptavidin-coated beads that had been coupled to biotinylated PfRH5 antigen. The PfRH5-coupled beads were incubated with plasma samples for 1 h at 37°C, then washed and incubated with IdeZ enzyme (New England Biolabs, #P0770S) for 1 h at 37°C to remove the Fc fragments from the bead-bound antibodies. These Fc fragments were transferred to new tubes and incubated with PNGase F (Applied Biosystems, #A28404) for 1 h at 50°C to remove the glycans. The glycans were then isolated and labeled with APTS dye using a GlycanAssure kit (Thermo Fisher, #A28676) according to the manufacturer’s instructions. Finally, APTS-labeled glycan samples were analyzed by capillary electrophoresis on an ABI 3500xL Genetic Analyzer. The area under the peak for each Fc glycan structure was calculated using GlycanAssure data analysis software. Results were then reported as the frequency (%) of each glycan structure within a given sample.

### Quantification and statistical analysis

Unless otherwise stated, data were analyzed using GraphPad Prism version 8.3.1 for Windows (GraphPad Software Inc.). All tests used were 2-tailed and are described in the text, along with the protocol pre-specified analyses in the relevant Results sections. To analyze the relationship between GIA and ELISA assay data, a Richard’s five-parameter dose-response curve was fitted, constrained to 0% GIA at the bottom and 100% GIA at the top. A value of p < 0.05 was considered significant.

#### Modeling of PMR

qPCR-derived PMR was pre-specified in the VAC063 trial protocol as the primary efficacy endpoint, and the comparison of the endpoint between the two groups in VAC063A constituted the pre-specified primary analysis for RH5.1/AS01_B_ vaccine efficacy. PMR was modeled as previously reported;^44^ here, in order to model the PMR, the arithmetic mean of the three replicate qPCR results obtained for each individual at each time-point was used for model-fitting. Negative individual replicates were assigned a value of 0 p/mL for the purposes of calculating the arithmetic mean of triplicates (where at least one of the three readings was positive). All qPCR data points which, based upon the mean of the three replicates, are greater than the LLD (> 5 p/mL) were used for modeling. Any values ranging from 1-5 p/mL were replaced with a value = LLD (i.e., 5 p/mL). Any data point that was negative but preceded a positive data point was replaced with a value = LLD (i.e., 5 p/mL); otherwise negative data points occurring after any positive data point but not preceding a positive data point were treated as 0 p/mL. PMR was then calculated using a linear model fitted to log_10_-transformed qPCR data.^27^ On day of CHMI for both VAC063A and VAC063B, all volunteers were inoculated by midday. Therefore, for modeling purposes, morning follow-up bleeds were taken to occur at 9:00am (i.e., dC+1 was 0.9 d post-infection), and the evening bleeds were taken to occur at 6:00pm (i.e., 0.37 d later). As previously, fitted lines were constrained to pass through the known starting parasitemia, calculated from the results of the limiting-dilution-based assay of the number of viable parasites in the inoculum, and a weight-based estimate of each volunteer’s blood volume (70 mL/kg).^40^ PMR was modeled for all volunteers that underwent blood-stage CHMI, given they all had ≥ 5 data points above the LLQ (the criterion for proceeding to model the PMR).^40^

#### Modeling of Antibody Kinetics

See Data S2A.

#### Computational Analysis of Systems Serology Data

Spearman and Pearson correlation coefficients were used as indicated in the corresponding Figures. For assessing the statistical significance, Benjamini-Hochberg correction for multiple hypothesis testing was used. Random forest regression using the ‘randomForest’ package of R was used to model DOD or IVGI based on the systems serology data. To take into account that some volunteers are in both Group 5 and Group 7, we used a leave-one-volunteer-out cross-validation framework that yielded 13-folds. For each volunteer, the model was built on data of the remaining volunteers and then used to predict the phenotype of the holdout volunteer. The model prediction for the phenotype was compared to the observed phenotype based on the Pearson’s product moment correlation coefficient and corresponding *P value*s (R function ‘cor.test’). The random forest regression was repeated 100 times and the correlation coefficients and *P value*s are shown in Figures S10D and S10E. To assess the importance of the features within the leave-one-volunteer-out cross-validation framework, 100 repetitions of a recursive feature elimination (RFE) were performed for each of the 13-folds. In each repetition, a random forest model is generated for the full feature set, and recursively the feature with the lowest importance determined via the mean decrease in mean squared error is removed. For each of the recursively obtained models, the out-of-bag mean squared error in prediction is determined and the feature set corresponding to the best performing model is chosen. Overall, we count how often a feature is chosen in the final feature set. The regression model was compared to a model that was trained and tested for shuffled phenotype labels. An R notebook (analysis_Rh5.Rmd) for the analysis is available as Data S2B.

## Additional resources

ClinicalTrials.gov: https://clinicaltrials.gov/ct2/show/NCT02927145

## Supplemental information

Document S1. Figures S1–S10

Data S1 . Vaccine safety and qPCR data, related to Figures 1 and 3 (A) Laboratory AEs in vaccinated volunteers, Groups 1-5 and 7, considered possibly, probably or definitely related to vaccination. (B) Unsolicited AEs considered possibly, probably or definitely related to vaccination. As well as the list of solicited AEs collected at each visit, participants were also asked to report any other AEs that occurred, and these were recorded and assigned a MedDRA code. AEs occurring ≤ 28 days after each vaccination are shown, with maximum severity reported. Time-point(s) = days post-vaccination. (C) Raw qPCR data (parasites/mL) for VAC063A. (D) Raw qPCR data (parasites/mL) for VAC063B.

Data S2 . Modeling information, related to Figures 2 and 6 (A) Modeling of antibody kinetics. (B) R notebook (analysis_Rh5.Rmd) for the systems serology analysis.

Document S2. Article plus supplemental information
