# Supplementary material for: Reduced blood-stage malaria growth and immune correlates in humans following RH5 vaccination
Source: Med. 2021 Jun 11;2(6):701–719.e19. doi: 10.1016/j.medj.2021.03.014 (PMC8240500; doi:10.1016/j.medj.2021.03.014)
Supplement: Document S1. Figures S1–S10 [file mmc1.pdf]

Med, Volume *2*

Supplemental information

Reduced blood-stage malaria growth and immune correlates in humans following RH5 vaccination

Angela M. Minassian, Sarah E. Silk, Jordan R. Barrett, Carolyn M. Nielsen, Kazutoyo Miura, Ababacar Diouf, Carolin Loos, Jonathan K. Fallon, Ashlin R. Michell, Michael T. White, Nick J. Edwards, Ian D. Poulton, Celia H. Mitton, Ruth O. Payne, Michael Marks, Hector Maxwell-Scott, Antonio Querol-Rubiera, Karen Bisnauthsing, Rahul Batra, Tatiana Ogrina, Nathan J. Brendish, Yrene Themistocleous, Thomas A. Rawlinson, Katherine J. Ellis, Doris Quinkert, Megan Baker, Raquel Lopez Ramon, Fernando Ramos Lopez, Lea Barfod, Pedro M. Folegatti, Daniel Silman, Mehreen Datoo, Iona J. Taylor, Jing Jin, David Pulido, Alexander D. Douglas, Willem A. de Jongh, Robert Smith, Eleanor Berrie, Amy R. Noe, Carter L. Diggs, Lorraine A. Soisson, Rebecca Ashfield, Saul N. Faust, Anna L. Goodman, Alison M. Lawrie, Fay L. Nugent, Galit Alter, Carole A. Long, and Simon J. Draper

	Group	Intended Group Size *	Day 0	Day 28	Day 56	Day 182
Phase Ia	1	12	2 µg RH5.1 / 0.5mL AS01$_B$	2 µg RH5.1 / 0.5mL AS01$_B$	2 µg RH5.1 / 0.5mL AS01$_B$	
	2	12	10 µg RH5.1 / 0.5mL AS01$_B$	10 µg RH5.1 / 0.5mL AS01$_B$	10 µg RH5.1 / 0.5mL AS01$_B$	
	3	12	50 µg RH5.1 / 0.5mL AS01$_B$	50µg RH5.1 / 0.5mL AS01$_B$		10 µg RH5.1 / 0.5mL AS01$_B$
	4	12	50 µg RH5.1 / 0.5mL AS01$_B$	50 µg RH5.1 / 0.5mL AS01$_B$	50 µg RH5.1 / 0.5mL AS01$_B$	

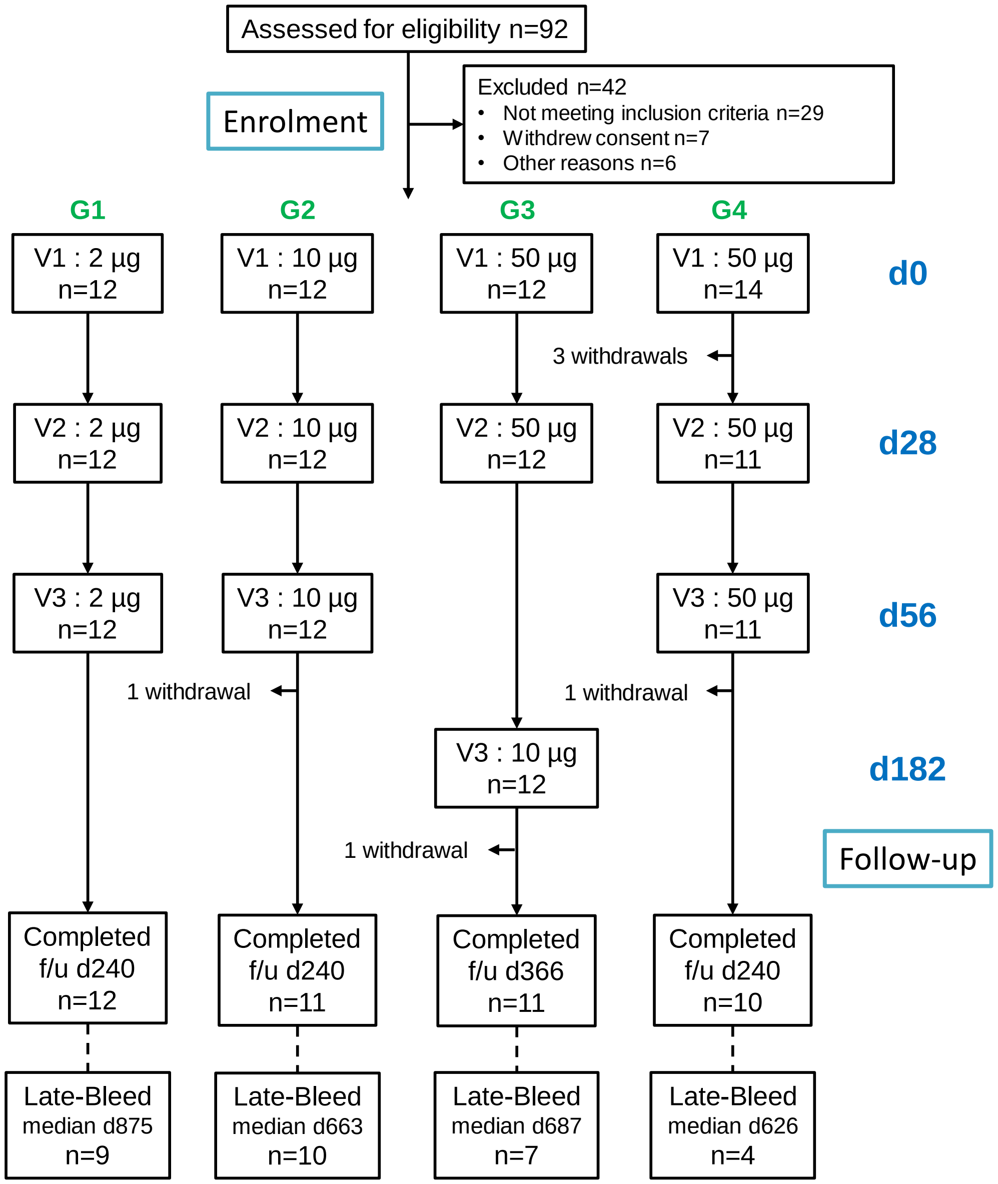

Figure S1. VAC063 Phase Ia Groups 1-4 table and flow chart of study design and volunteer recruitment; related to Figure 1.

Timing of the three vaccinations (V1-V3), dose of RH5.1 (in µg) and trial follow-up (f/u) period are shown. All adjuvant doses were 0.5 mL AS01$_B$. Enrolment into the VAC063 Phase Ia study began on 17 October 2016 and all primary follow-up visits (d240 or d366) were completed by 30 July 2018. All

immunizations were administered i.m. into the deltoid of the non-dominant arm preferentially. Seventeen males and 33 females were enrolled, the mean age was 30 years (range 18 – 45 years). Six volunteers withdrew from the trial during this period as indicated. One volunteer in Group 2 withdrew consent after d84 and one volunteer in Group 3 withdrew consent after d182. Three volunteers withdrew from Group 4 prior to the second vaccination; one volunteer was withdrawn by the Investigators on d14 due to anemia (a pre-existing condition that had not been disclosed at screening), and two withdrew consent just prior to d28. Two of these volunteers were subsequently replaced, thereby n=14 received the first vaccination but only n=11 went on to receive a second and third vaccination. A fourth volunteer withdrew consent after d140 in Group 4. Volunteers were subsequently invited back for a late-bleed and 30 consented. These bleeds began in February 2018 and were completed by 27 June 2019. * In the summary table, intended group size (as per protocol) is indicated.

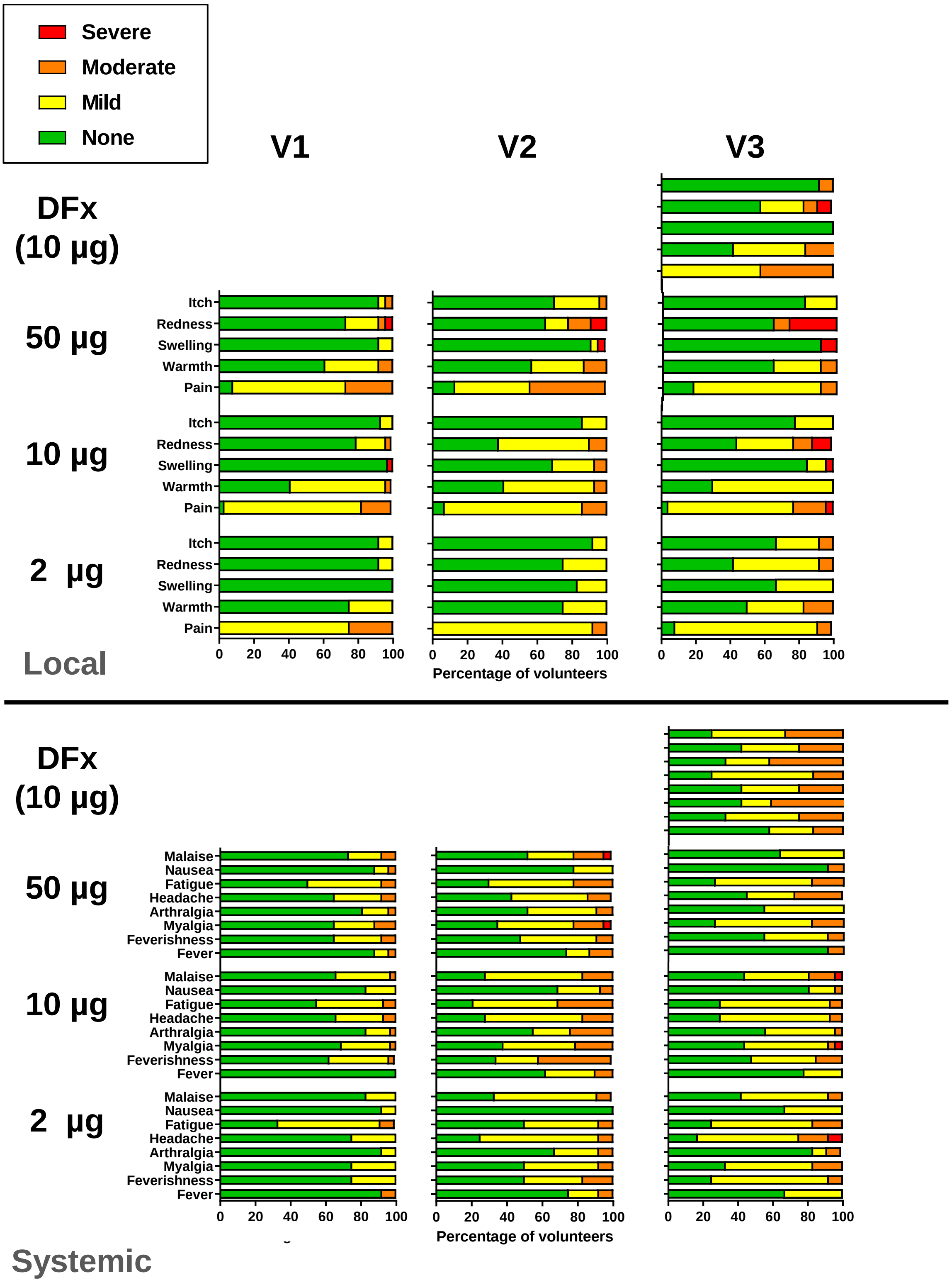

Figure S2. Solicited AEs following three vaccinations with 2, 10 or 50 µg RH5.1/AS01$_B$; related to Figure 1.

The solicited local and systemic adverse events (AEs) recorded for 7 days following RH5.1/AS01$_B$ vaccination are shown at the maximum severity reported by all volunteers and as a percentage of

volunteers reporting each individual AE. Data are shown following each of three vaccinations (V1, V2 and V3; running horizontally) at the stated dose and regimen of RH5.1 formulated in the same volume of $AS01_B$ adjuvant (running vertically). Data for this figure are combined across all volunteers who were vaccinated in the VAC063 study (i.e. including all available data from Groups 1-5).

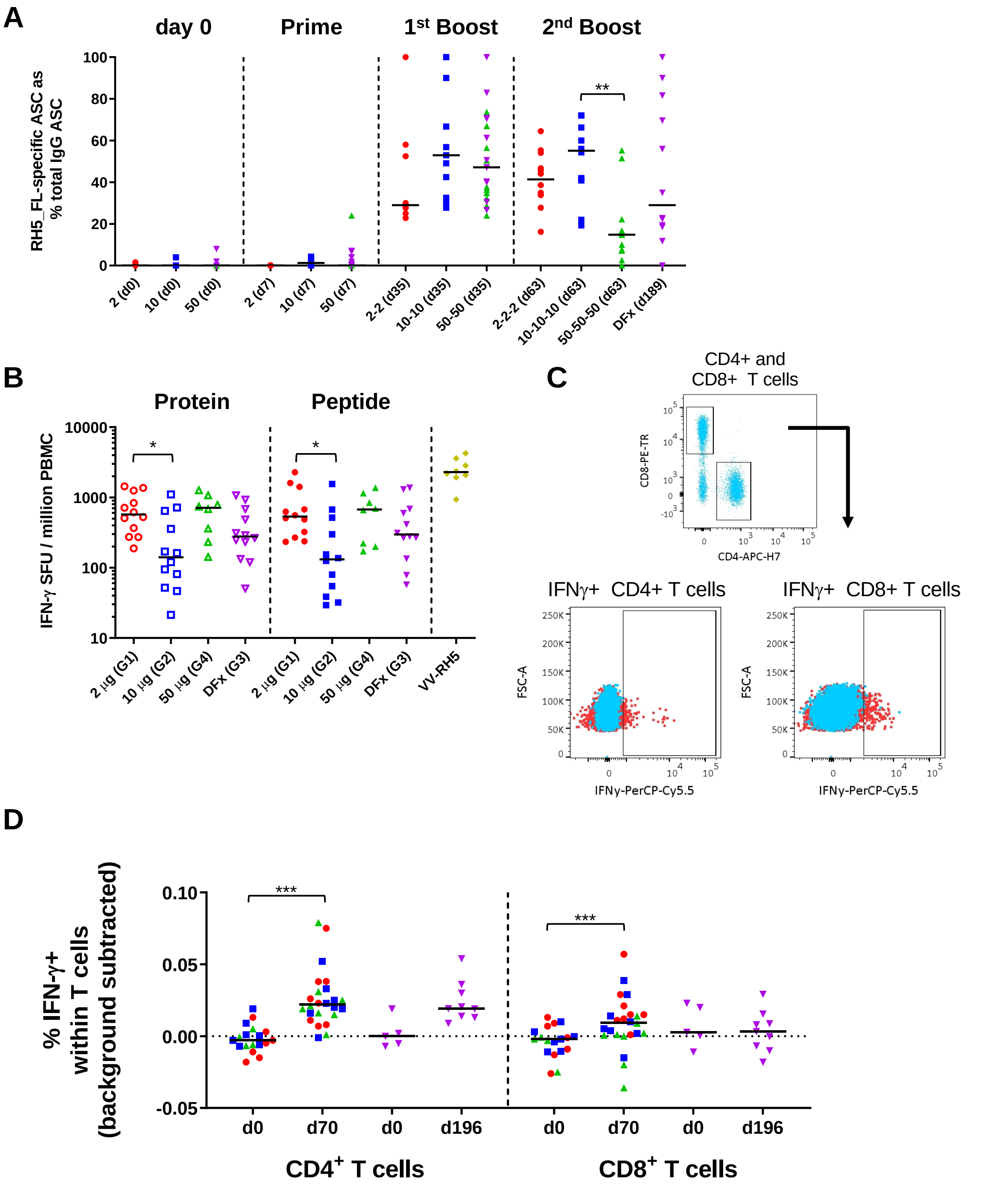

Figure S3. Anti-RH5 *ex-vivo* B cell and IFN-γ T cell responses; related to Figure 1.

(**A**) RH5-specific antibody-secreting cell (ASC) responses were assessed by *ex-vivo* ELISPOT using RH5_FL protein and fresh PBMC at baseline (d0) and 7 days after each of the three vaccinations (d7, d35 and d63/d189) in Groups 1-4. Individual and median responses are shown for each dosing

regimen and are reported as RH5_FL-specific ASC as a % of all IgG ASC detected. Statistical analysis of Groups 1-4 at d63/189 using Kruskal-Wallis test with Dunn's multiple comparison test, ** $P<0.01$. (**B**) RH5-specific T cell responses were assessed by *ex-vivo* IFN-γ ELISPOT in fresh PBMC following stimulation with either RH5.1 protein (open symbols) or overlapping peptides (closed symbols). For peptides, the summed response across six individual peptide pools is reported. Median and individual responses are shown from the 2 weeks post-final boost time-point (d70 for Groups 1, 2 and 4, and d196 for Group 3). Historical data for the VV-RH5 vaccine [11] are shown for comparison. Statistical analysis of Groups 1-4 using Kruskal-Wallis test with Dunn's multiple comparison test, * $P<0.05$ (tested separately for each stimulation condition). (**C**) Frozen PBMC from d0 or two-weeks post-final vaccination (Groups 1/2/4 = d70, Group 3 = d196) were stimulated with medium alone, RH5.1 protein (1 µg/mL) or SEB (1 µg/mL; positive control [not shown]) for 24 h, with brefeldin A at 3 µg/mL for the final 2 h, then stained and analyzed by flow cytometry. Gating strategy for definition of total $CD4^+$ and $CD8^+$ T cells within the live T cell population, and for IFN-γ^+ cells within the $CD4^+$ or $CD8^+$ T cell populations. Blue = medium alone; red = SEB stimulation. (**D**) Median and individual responses are shown for the % $CD4^+$ and $CD8^+$ T cells positive for IFN-γ. Data for Groups 1, 2 and 4 are combined (monthly dosing) versus the DFx regimen (Group 3). The frequency of IFN-γ^+ cells in sample-matched unstimulated wells was subtracted from the frequency in RH5.1-stimulated wells to control for non-specific activation. Samples were excluded from analysis if the parent population contained <50 cells. G1/2/4 d0 n=18, d70 n=24; G3 d0 n=5, d196 n=9. *** $P<0.001$; Wilcoxon matched-pairs signed rank test.

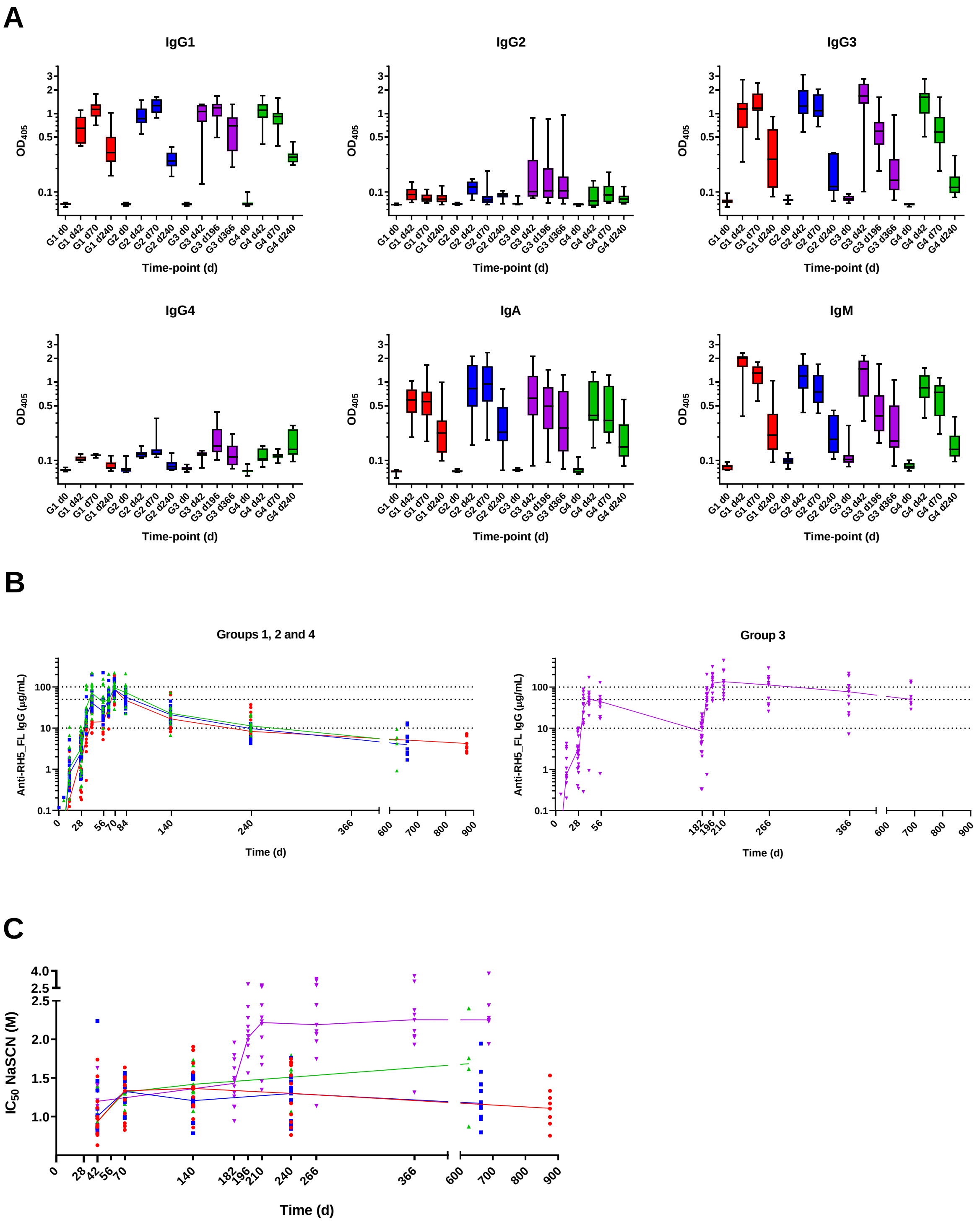

Figure S4. Anti-RH5 serum antibody response over time; related to Figure 2.

(**A**) Isotype and subclass profiles of serum antibody responses against RH5_FL were assessed by ELISA. Responses are shown for all Groups at baseline (d0) and at 14 days after two immunizations (d42), 14 days after three immunizations (d70 or d196) and at the completion of follow-up (d240 or

d366). Box and whisker plots show median, interquartile range and range. (**B**) Median and individual anti-RH5_FL serum total IgG responses shown for Groups 1-4 over time. Dotted lines highlight 10, 50 and 100 µg/mL levels. (**C**) Median and individual avidity of serum total IgG responses shown for Groups 1-4 over time as assessed by NaSCN-displacement RH5_FL ELISA. Avidity is reported as the molar (M) concentration of NaSCN required to reduce the starting OD in the ELISA by 50% (IC_{50}).

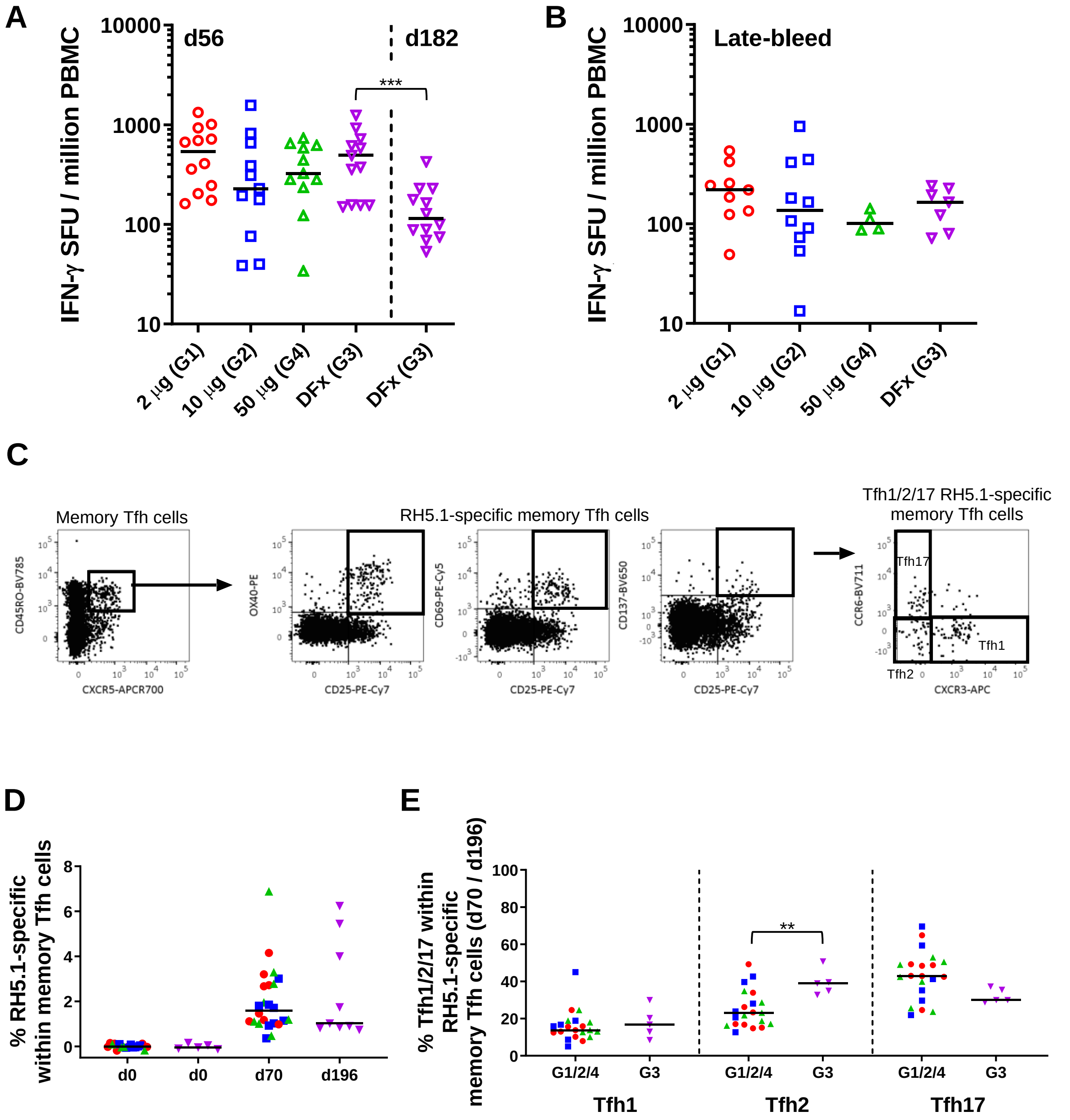

Figure S5. Anti-RH5 IFN-γ T cell responses and peripheral RH5.1-specific memory CD4⁺ Tfh cell responses after monthly versus DFx vaccine dosing; related to Figure 2.

RH5-specific T cell responses were assessed by *ex-vivo* IFN-γ ELISPOT in fresh PBMC and following stimulation with RH5.1 protein. Median and individual responses are shown for (**A**) the indicated groups at d56 (the day of the third immunization in Groups 1, 2 and 4) and d182 (the day of the third DFx immunization in Group 3); and (**B**) the late-bleed time-point ~2 years later. Statistical analysis of Group 3 at the two time-points using Wilcoxon matched-pairs signed rank test, ***

P<0.001. (**C**) Frozen PBMC from day 0 (d0) or two weeks post-final vaccination (Groups 1/2/4 = d70; Group 3 = d196) were stimulated with medium alone, RH5.1 protein (1 µg/mL) or SEB (1 µg/mL; positive control [not shown]) for 24 h, with brefeldin A at 3 µg/mL for the final 2 h, then stained and analyzed by flow cytometry. Gating strategy for definition of memory circulating Tfh cells as $CXCR5^{+}CD45RO^{+}$ cells within the $CD4^{+}$ T cell population, and for definition of RH5.1-specific cells as those co-expressing CD25 with OX40, or CD137 or CD69 after stimulation with RH5.1. Tfh1/2/17 cells within the RH5.1-specific memory Tfh cell population were defined based on expression of CCR6 and CXCR3. (**D**) Frequencies of RH5.1-specific cells within the memory Tfh cell population; G1/2/4 d0 n=18, d70 n=24; G3 d0 n=5, d196 n=9. The frequency of activated cells in sample-matched unstimulated wells was subtracted from the frequency in RH5.1-stimulated wells to control for non-specific activation. (**E**) Proportions of Tfh1/2/17 cells within the RH5.1-specific memory Tfh cells; G1/2/4 d70 n=21; G3 d196 n=5. Samples were excluded from analysis if the parent population contained <50 cells. Each point represents a single vaccinee and lines denote medians. ** *P*<0.01, Mann-Whitney test comparing G3 (DFx regimen) vs G1/2/4 (monthly dosing).

	Group	Intended Group Size *	Day 0	Day 28	Day 56	2 weeks post-third vaccination	~4 months post-third vaccination	2 weeks post-final vaccination
Phase IIa CHMI	5 (vaccinees)	15	10 µg RH5.1/ 0.5mL $AS01_B$	10 µg RH5.1/ 0.5mL $AS01_B$	10 µg RH5.1/ 0.5mL $AS01_B$	CHMI		
	6 (controls)	15				CHMI		
	7 (Subset of Group 5 vaccinees)	5-15	10 µg RH5.1/ 0.5mL $AS01_B$	10 µg RH5.1/ 0.5mL $AS01_B$	10 µg RH5.1/ 0.5mL $AS01_B$	CHMI	10 µg RH5.1/ 0.5mL $AS01_B$	CHMI
	8 (Subset of Group 6 controls)	5-15				CHMI		CHMI
	9 (New controls)	5-6						CHMI

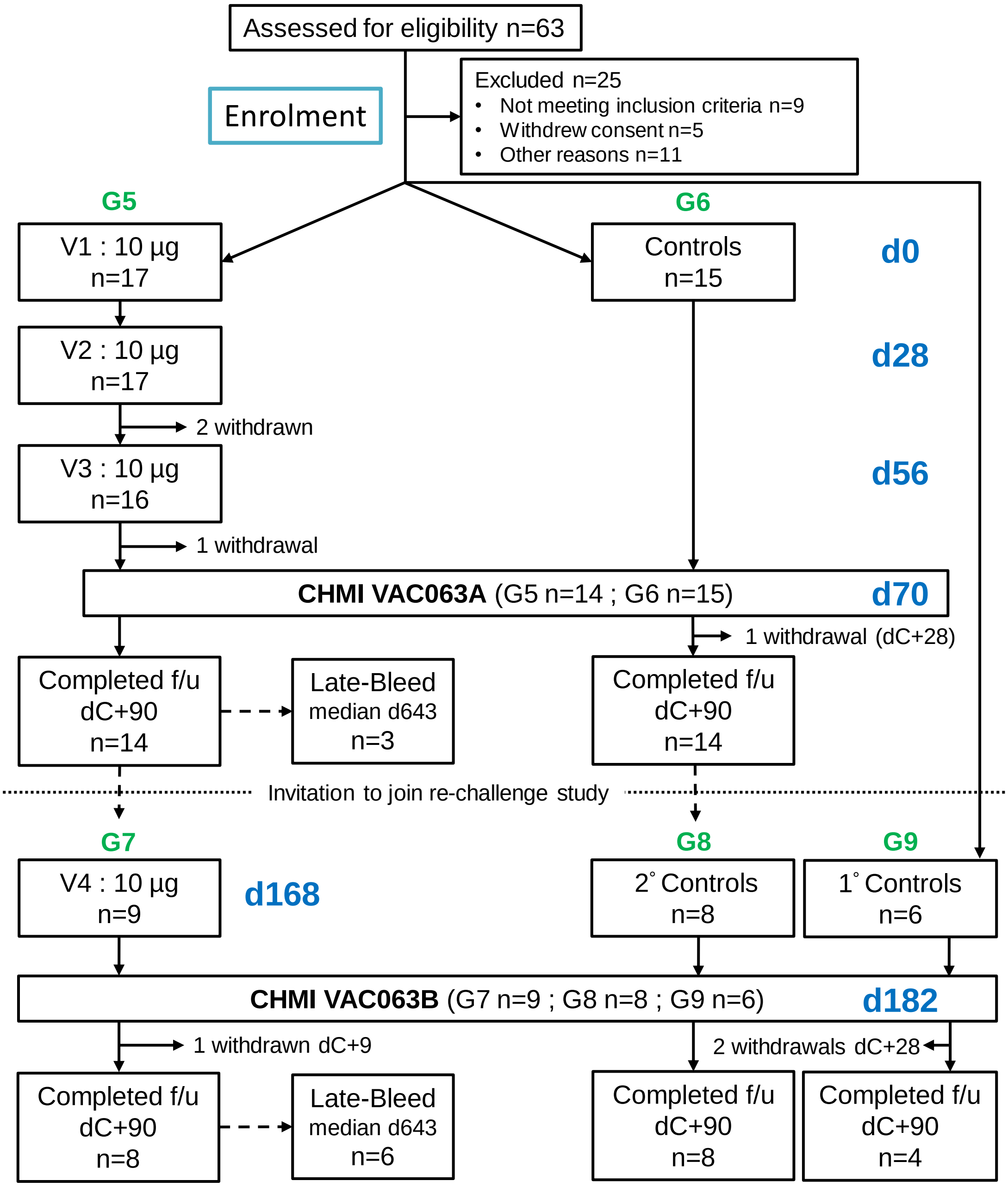

Figure S6. VAC063 Phase IIa Groups 5-9 table and flow chart of study design and volunteer recruitment; related to Figure 3.

Enrolment into the VAC063 Phase IIa study began on 4 September 2017. The timing of the three 10 µg RH5.1 vaccinations (V1-V3) in Group 5 are shown and all adjuvant doses were 0.5 mL $AS01_B$. All immunizations were administered i.m. into the deltoid of the non-dominant arm preferentially. Primary blood-stage CHMI (VAC063A) occurred in Groups 5 and 6 on 14 November 2017 with trial primary follow-up (f/u) until 90 days after challenge (dC+90) completed by 15 February 2018. Two volunteers in Group 5 were withdrawn after the second vaccination; one due to difficulty tolerating venepuncture and one due to development of mild bilateral infraorbital swelling within 24 h of second vaccination (which subsequently resolved within 48 h – also see STAR Methods Safety Analysis section). One volunteer in Group 5 withdrew consent at dC-1 and one volunteer in Group 6 withdrew consent at dC+28. Volunteers were subsequently invited back for a re-challenge and 17 consented (with Groups 5 and 6 being renamed Groups 7 and 8 respectively). Group 7 received a fourth 10 µg RH5.1 vaccination (V4) including the adjuvant dose of 0.5 mL $AS01_B$. A new group of unvaccinated infectivity controls (Group 9) was also recruited and enrolled on 5 March 2018. Secondary blood-stage CHMI (VAC063B) occurred in Groups 7-9 on 6 March 2018 with trial f/u until dC+90 completed by 6 June 2018. Three volunteers withdrew from the trial during this period as indicated – two of these withdrew consent at dC+28 and one volunteer was withdrawn on dC+9 post-diagnosis due to a detected pregnancy (also see STAR Methods Safety Analysis section). All volunteers in Group 5/7 were subsequently invited back for a late-bleed and nine consented. These bleeds began on 22 May 2019 and were completed by 20 June 2019. Across Groups 5, 6 and 9, 16 males and 22 females were enrolled, the mean age was 27 years (range 21 – 39 years). * In the summary table, intended group size (as per protocol) is indicated.

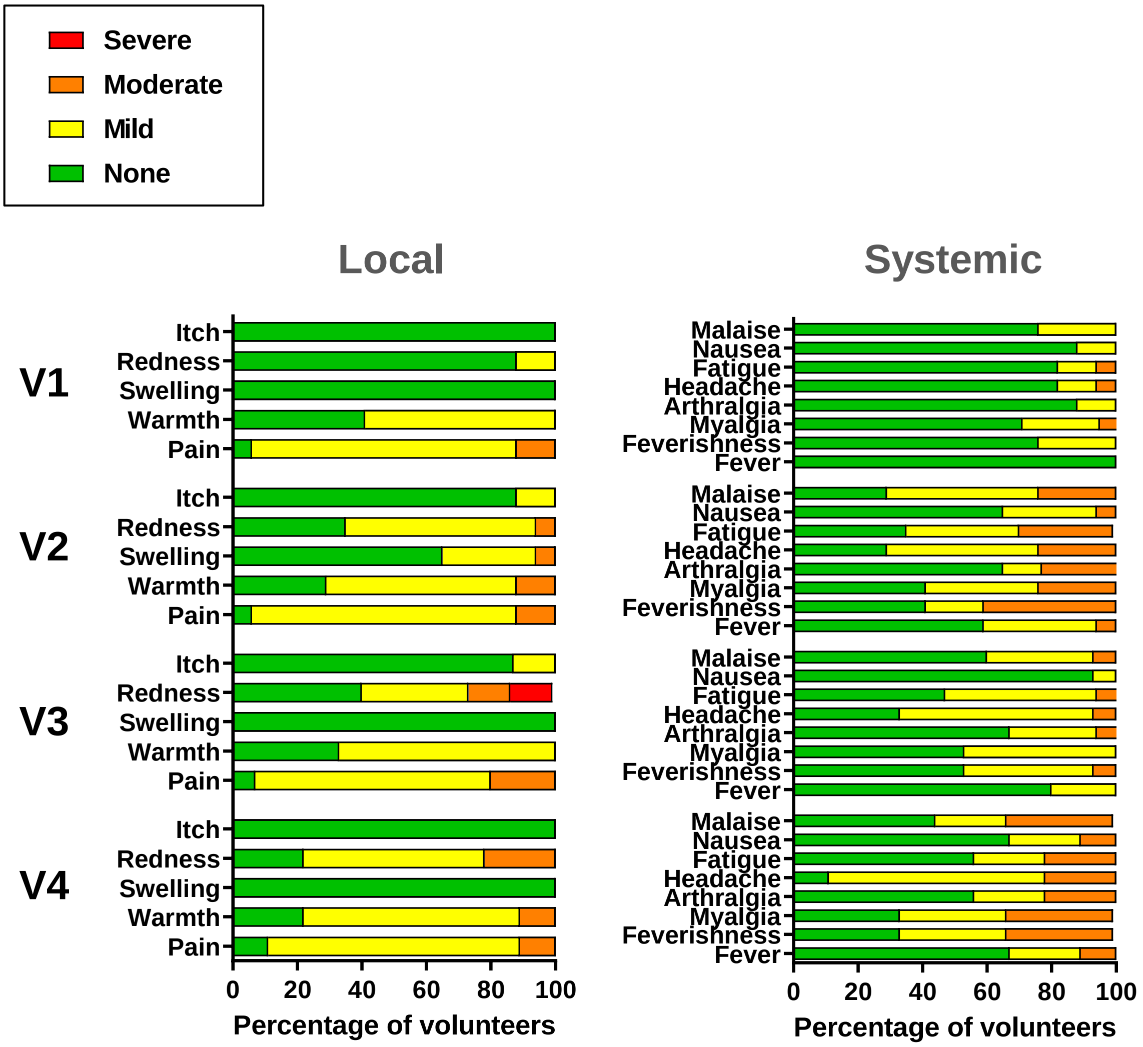

Figure S7. Solicited AEs following four vaccinations with 10 µg RH5.1/AS01$_B$ in Group 5/7; related to Figure 3.

The solicited local and systemic AEs recorded for 7 days following RH5.1/AS01$_B$ vaccination are shown at the maximum severity reported by all volunteers. Data are shown following each of the four 10 µg RH5.1 vaccinations formulated in the same volume (0.5 mL) of AS01$_B$ adjuvant and administered to the volunteers in Group 5/7.

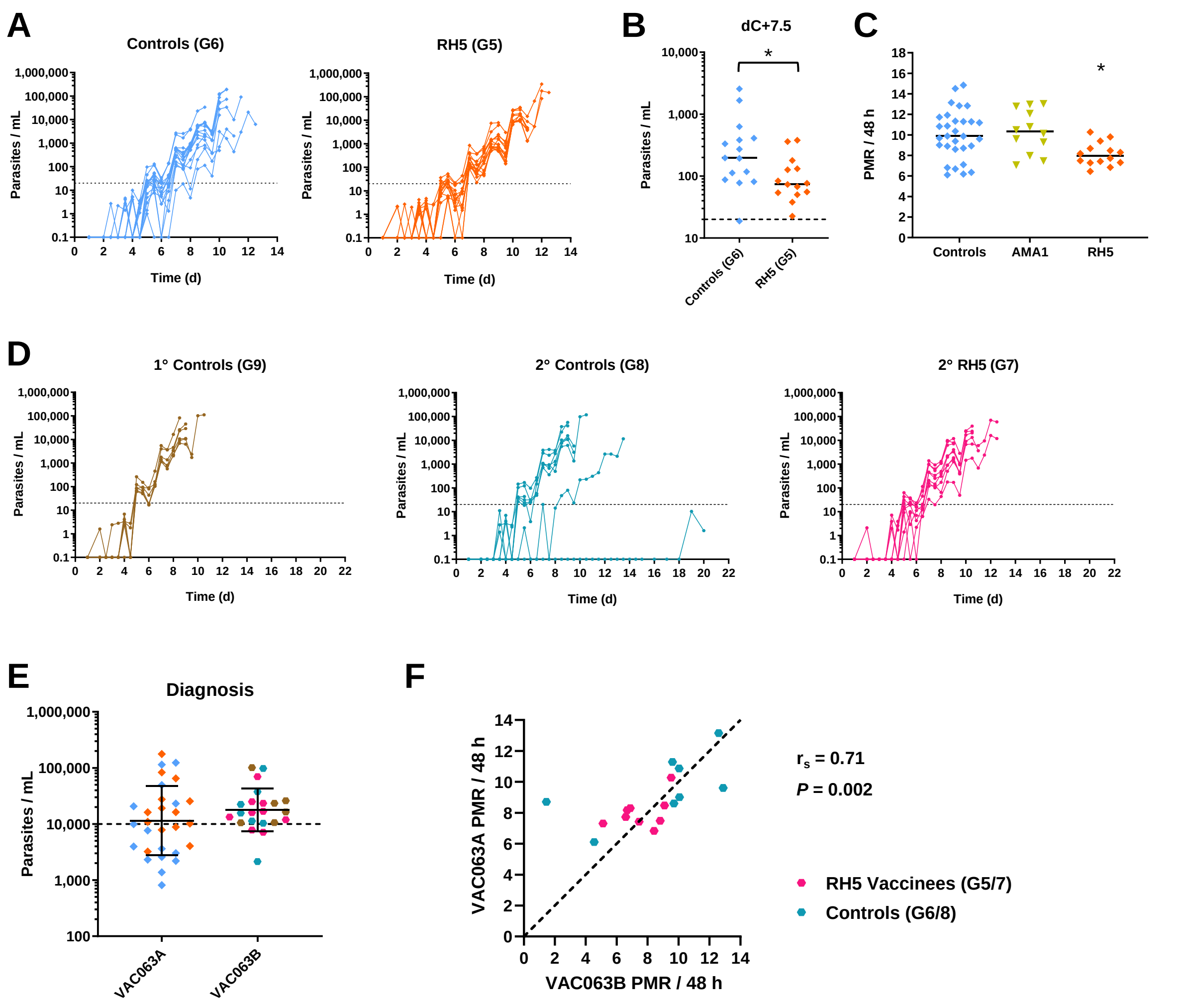

Figure S8. Parasitemia data following primary and secondary blood-stage CHMI; related to Figure 3.

(**A**) qPCR data for the VAC063A Phase IIa study; Group 5 vaccinees (*n*=14) and Group 6 controls (*n*=15). Individual parasitemia data are shown over time. The lower limit of quantification is indicated by the dotted line at 20 p/mL; values below this level are plotted for information only. Time=days (d) post blood-stage CHMI. (**B**) Individual and median p/mL data on dC+7.5. * P<0.05, Mann-Whitney test. (**C**) Post-hoc analysis combining the VAC063A dataset with the AMA1/$AS01_B$ trial (VAC054) data [18]. Median and individual PMRs are shown for controls (n=30); AMA1 vaccinees (n=12) and RH5 vaccinees (n=14). * P<0.05 for RH5 versus AMA1 and controls, using one-way ANOVA with Bonferroni correction for multiple comparisons. (**D**) qPCR data as in panel A for the VAC063B Phase IIa re-challenge study; Group 7 vaccinees (*n*=9), Group 8 secondary controls (n=8) and Group 9

primary controls (*n*=6). One individual in Group 8 reached a maximum parasitemia of 10 p/mL on dC+19. This volunteer requested treatment on dC+20, at which point a blood sample was placed into *in vitro* culture and outgrowth of parasites was confirmed (data not shown). (**E**) Parasitemia at diagnosis for all volunteers in VAC063A (n=29) and VAC063B (n=22, one volunteer excluded from analysis due to early malaria treatment for other reason). Geometric mean and SD are shown. Volunteers were diagnosed in VAC063A according to an algorithm based on patency by thick-film microscopy and/or pre-defined thresholds of p/mL blood by qPCR and/or symptoms, whereas in VAC063B they were diagnosed according to an algorithm based only on pre-defined thresholds of p/mL blood by qPCR and/or symptoms. Removal of thick-film microscopy from the diagnostic algorithm in VAC063B greatly reduced the number of volunteers diagnosed prematurely (i.e. <5,000 p/mL). This change gave similar mean p/mL at diagnosis but reduced the spread of the data. Geomean p/mL in VAC063A = 11,497 p/mL, SD = 43,395; versus VAC063B geomean = 17,860 p/mL, SD = 27,393. *P*=0.033, F test on $\log_{10}$ transformed data. (**F**) Correlation of PMR observed in Group 7 and 8 volunteers in VAC063B versus their PMR in VAC063A (when they were in Groups 5 and 6, respectively). Spearman's rank correlation coefficient and *P* value are shown, n=17. Line of identity (X=Y) is also shown (black dashed line).

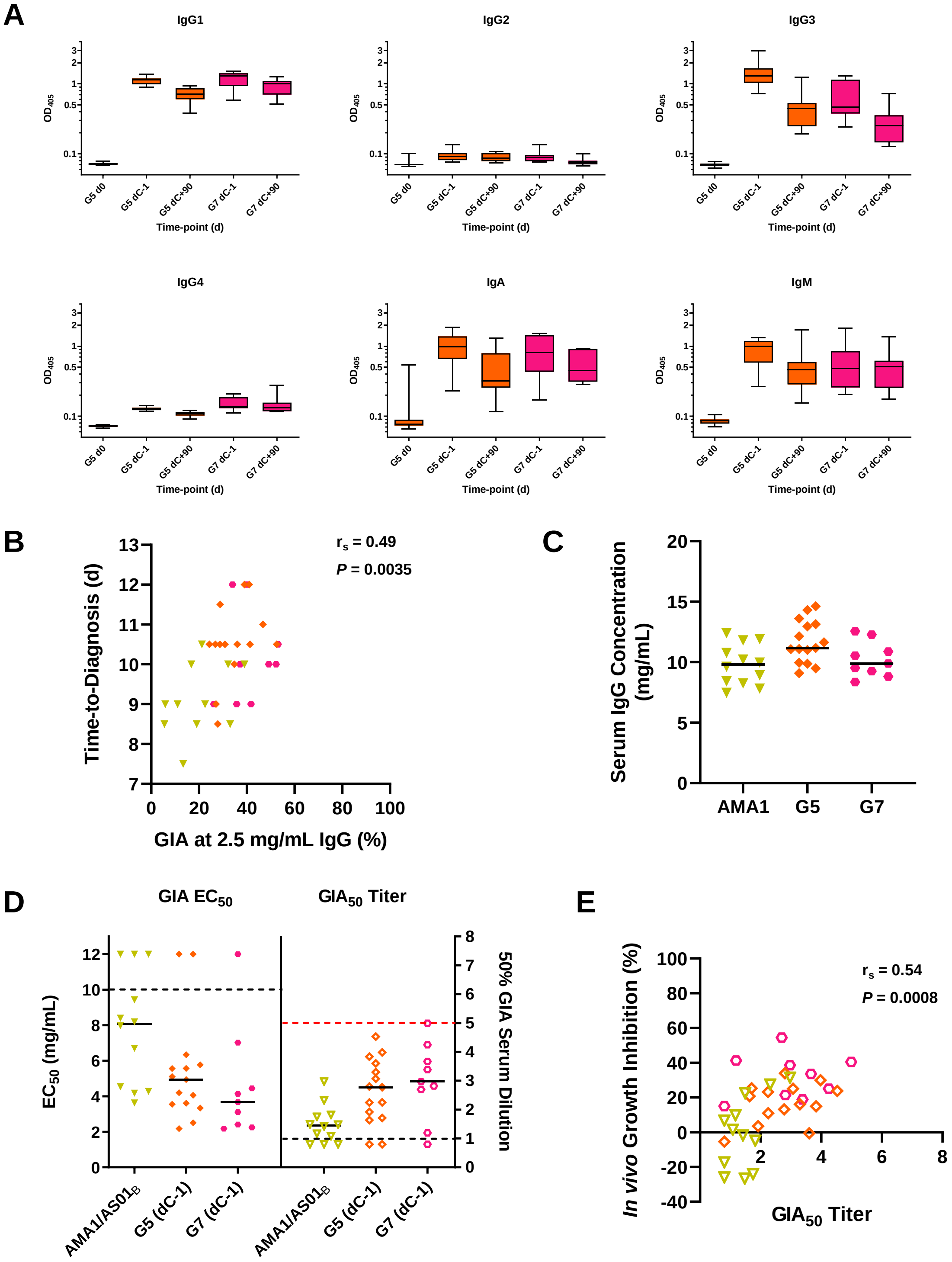

Figure S9. Serum antibody analyses of RH5 and AMA1 vaccinees undergoing CHMI; related to Figure 5.

(**A**) Isotype and subclass profiles of serum antibody responses against RH5_FL were assessed by ELISA. Responses are shown for Group 5 at baseline (G5 d0); following three RH5.1/AS01$_B$

immunizations and the day before primary CHMI (G5 dC-1); 90 days after primary CHMI (G5 dC+90); following the fourth RH5.1/AS01$_B$ immunization and the day before secondary CHMI (G7 dC-1); and 90 days after secondary CHMI (G7 dC+90). Box and whisker plots show median, interquartile range and range. (**B**) Correlation of diagnosis time-point (day; DOD) for each individual following blood-stage CHMI versus their individual *in vitro* GIA measured at dC-1 using 2.5 mg/mL purified IgG. Spearman's rank correlation coefficient and *P* value are shown, n=34 (including all vaccinated volunteers in the AMA1/AS01$_B$ vaccine trial [18], and G5 and G7 vaccinees). (**C**) Total serum IgG concentrations in mg/mL at the dC-1 time-point for the vaccinated volunteers in the AMA1/AS01$_B$ vaccine trial [18], and for G5 and G7 volunteers. Median and individual results are shown. (**D**) Individual GIA assay EC_{50} of each purified IgG in mg/mL is shown (left), i.e. the concentration of total IgG that, following titration, showed 50% GIA. Samples for which the GIA was <50% at 10 mg/mL (dashed black line) were plotted as 12 mg/mL. To relate the GIA assay results (using a normalized concentration of purified IgG) back to the original sera, the concentration of IgG in each original serum sample was also measured by HPLC (see panel C). This enabled calculation of the GIA_{50} serum titer (right), defined previously [17] as the dilution factor of each serum sample required to reach the concentration of purified IgG that gives 50% GIA. Samples for which the GIA_{50} serum titer could not be calculated (because they did not achieve ≥50% GIA using 10 mg/mL purified IgG) are plotted arbitrarily at 0.8. Individual data and median results are shown for each vaccinee in G5 and G7, and for comparison, data are also included from the previously reported AMA1 FMP2.1/AS01$_B$ vaccine trial. *Aotus* monkeys, previously vaccinated with RH5, were protected against blood-stage *P. falciparum* challenge if they achieved a GIA_{50} titer > 5 [17], indicated by the dashed red line. (**E**) Correlation of % IVGI observed in each individual following blood-stage CHMI versus their individual *in vitro* GIA_{50} titer. Spearman's rank correlation coefficient and *P* value are shown, n=35.

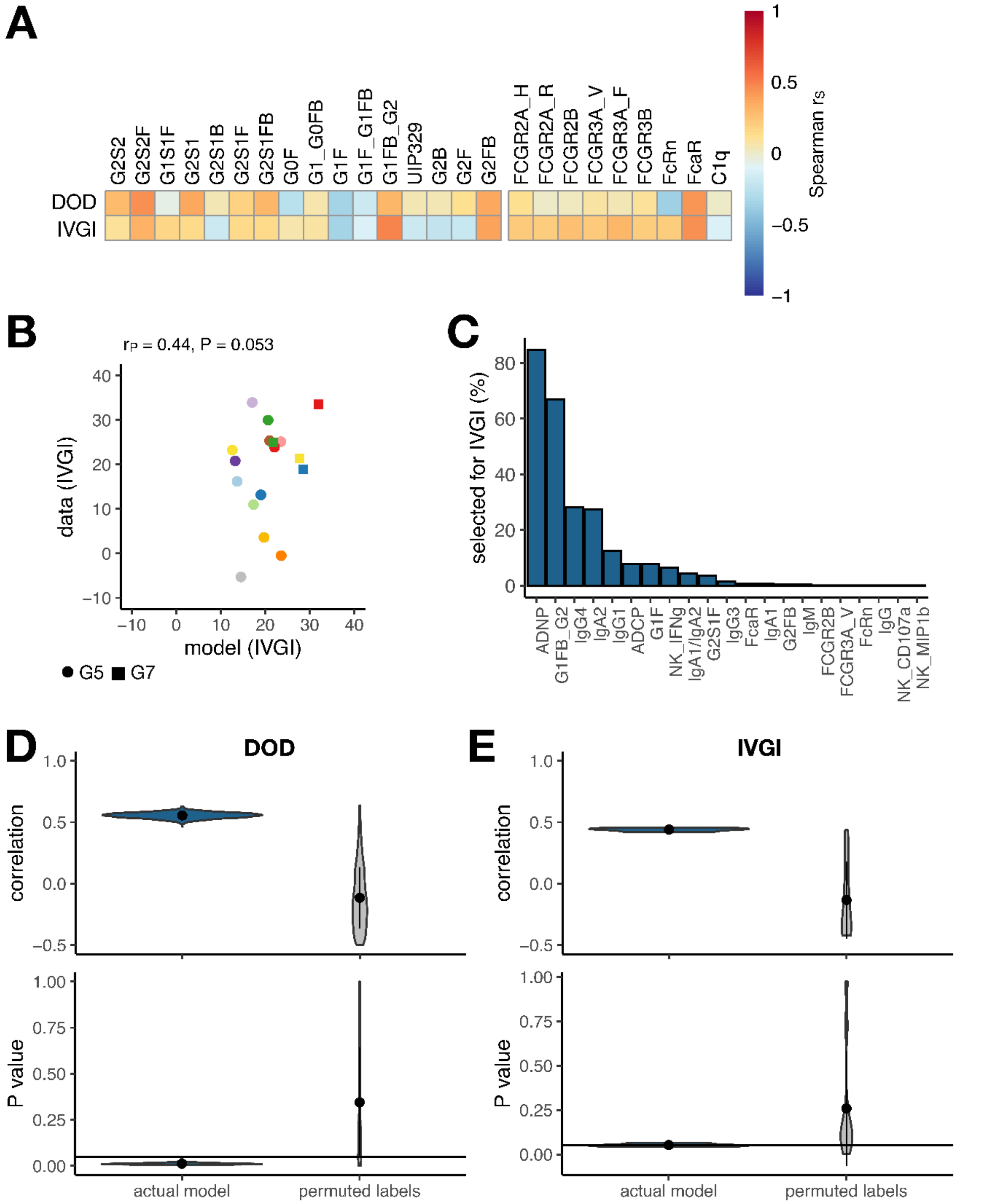

Figure S10. Systems serology analysis of RH5 vaccinees undergoing CHMI; related to Figure 6.

(**A**) Correlation heatmap showing the Spearman rank correlation coefficients (r_S) of the glycan and Fc- and complement receptor analyses to the CHMI readouts of DOD and IVGI. (**B**) Prediction for the random forest regression model plotted against the data for IVGI. The model was obtained using

leave-one-volunteer-out cross-validation. The Pearson correlation coefficient r_P and *P* value are shown. (**C**) The antibody features in the predictive model for IVGI are ranked according to how often they are chosen for 100 repetitions of recursive feature elimination (RFE) in the leave-one-volunteer-out cross-validation. (**D,E**) To assess the significance of the models, permutation tests were performed; for 'permuted labels', the model was trained on (**D**) shuffled DOD or (**E**) IVGI labels. The horizontal line shows the significance level of $P = 0.05$. For the predictive model for IVGI, the median of the *P* values over 1000 repetitions is > 0.05.
