# Supplementary material for: Reduced blood-stage malaria growth and immune correlates in humans following RH5 vaccination
Source: Med. 2021 Jun 11;2(6):701–719.e19. doi: 10.1016/j.medj.2021.03.014 (PMC8240500; doi:10.1016/j.medj.2021.03.014)
Supplement: Data S1 — . Vaccine safety and qPCR data, related to Figures 1 and 3 (A) Laboratory AEs in vaccinated volunteers, Groups 1-5 and 7, considered possibly, probably or definitely related to vaccination. (B) Unsolicited AEs considered possibly, probably or definitely related to vaccination. As well as the list of solicited AEs collected at each visit, participants were also asked to report any other AEs that occurred, and these were recorded and assigned a MedDRA code. AEs occurring ≤ 28 days after each vaccination are shown, with maximum severity reported. Time-point(s) = days post-vaccination. (C) Raw qPCR data (parasites/mL) for VAC063A. (D) Raw qPCR data (parasites/mL) for VAC063B. [file mmc2.zip › Data S1A-D/Data S1A.pdf]

| | | | Time-point at which the AE was first identified | | | |
|---|---|---|---|---|---|---|
| | | | Post-Vaccine 1 | Post-Vaccine 2 | Post-Vaccine 3 | Post-Vaccine 4 |
| **Group** | **AE** | **Max severity** | **n =** | **n =** | **n =** | **n =** |
| **1** | **Anaemia** | **Mild** | | 1 | 1 | n/a |
| | **Eosinophilia** | **Mild** | 1 | | | |
| | **Neutrophilia** | **Mild** | | | 1 | |
| | **leukopenia** | **Mild** | | | 1 | |
| | **Lymphopenia** | **Severe** | 1 | | | |
| | **Hypokalaemia** | **Mild** | 1 | 1 | | |
| | **Hyperkalaemia** | **Severe** | | | 1 | |
| | **Raised ALT** | **Mild** | | | 1 | |
| | **Raised bilirubin** | **Mild** | | | 1 | |
| **2** | **Anaemia** | **Mild** | 1 | | | n/a |
| | | **Moderate** | | 1 | 1 | |
| | **Eosinophilia** | **Mild** | 1 | 1 | 1 | |
| | **Lymphocytosis** | **Mild** | | | 1 | |
| | **Leukocytosis** | **Mild** | 1 | 1 | | |
| | | **Moderate** | | | 1 | |
| | **Leukopenia** | **Mild** | 1 | 1 | 1 | |
| | **Lymphopenia** | **Mild** | 1 | 1 | 1 | |
| | **Neutropenia** | **Mild** | | 1 | 1 | |
| | **Hypokalaemia** | **Moderate** | 1 | | | |
| | **Raised urea** | **Mild** | 1 | | | |
| | **Low albumin** | **Mild** | | 1 | | |
| **3** | **Anaemia** | **Mild** | | 1 | 1 | n/a |
| | **Leukopenia** | **Mild** | 2 | 3 | 3 | |
| | **Lymphopenia** | **Mild** | 1 | 1 | | |
| | **Neutropaenia** | **Mild** | 1 | 2 | 2 | |

| | | | | | | |
|---|---|---|---|---|---|---|
| | **Thrombocytopaenia** | **Mild** | | 1 | | |
| | **Raised urea** | **Mild** | | | 1 | |
| | **Raised creatinine** | **Mild** | 1 | 1 | 1 | |
| | **Raised bilirubin** | **Mild** | 1 | 1 | | |
| **4** | **Anaemia** | **Mild** | 1 | | | n/a |
| | | **Severe** | 1 | | | |
| | **Leukocytosis** | **Mild** | | 1 | | |
| | **Neutrophilia** | **Mild** | | 1 | | |
| | **Hypokalaemia** | **Mild** | 1 | | 1 | |
| | **Raised creatinine** | **Mild** | 1 | | | |
| **5** | **Leukocytosis** | **Mild** | | 1 | | n/a |
| | **Lymphopaenia** | **Mild** | | 1 | 1 | |
| | **Hypokalaemia** | **Mild** | | 1 | | |
| | **Raised ALT** | **Mild** | | 1 | | |
| | **Raised urea** | **Moderate** | 1 | | | |
| | **Raised bilirubin** | **Mild** | | | 1 | |
| | | **Moderate** | 1 | 1 | | |
| | **Low albumin** | **Severe** | 1 | | | |
| **7** | **Anaemia** | **Mild** | n/a | | | 1 |

**Data S1A. Laboratory AEs in vaccinated volunteers, Groups 1-5 and 7, considered possibly, probably or definitely related to vaccination.**

Table shows laboratory AEs recorded within 28 days of each vaccine administration, and considered possibly, probably or definitely related to vaccination. For Groups 1, 2, and 4, relevant blood tests were carried out on days 7, 14, 28, 35, 42, 56, 63, 70 and 84. For Group 3, relevant blood tests were carried out on days 7, 14, 28, 35, 42, 56, 189, 196 and 210. For Group 5, relevant blood tests were carried out as for Group 2 up until day 63; blood was then taken on day 69 (dC-1), prior to primary CHMI on day 70. For Group 7 blood tests were as for Group 5 post-third vaccination, with a d7 bleed

and a d14 bleed (the latter coinciding with the VAC063B dC-1 visit). Blood tests were also carried out at other time-points if clinically indicated. Maximum reported severity is shown, graded as per site-specific grading tables; see STAR Methods section on Experimental Model and Subject Details for further information. Time-point(s) indicates whether AEs occurred after the first, second, third or fourth vaccination. ALT = alanine aminotransferase.
