# Supplementary material for: Reduced blood-stage malaria growth and immune correlates in humans following RH5 vaccination
Source: Med. 2021 Jun 11;2(6):701–719.e19. doi: 10.1016/j.medj.2021.03.014 (PMC8240500; doi:10.1016/j.medj.2021.03.014)
Supplement: Data S1 — . Vaccine safety and qPCR data, related to Figures 1 and 3 (A) Laboratory AEs in vaccinated volunteers, Groups 1-5 and 7, considered possibly, probably or definitely related to vaccination. (B) Unsolicited AEs considered possibly, probably or definitely related to vaccination. As well as the list of solicited AEs collected at each visit, participants were also asked to report any other AEs that occurred, and these were recorded and assigned a MedDRA code. AEs occurring ≤ 28 days after each vaccination are shown, with maximum severity reported. Time-point(s) = days post-vaccination. (C) Raw qPCR data (parasites/mL) for VAC063A. (D) Raw qPCR data (parasites/mL) for VAC063B. [file mmc2.zip › Data S1A-D/Data S1C.pdf]

Group Number	Group	DoD	D1	D2	D2.5	D3	D3.5	D4	D4.5	D5	D5.5	D6	D6.5	D7	D7.5	D8	D8.5	D9	D9.5	D10	D10.5	D11	D11.5	D12	D12.5
**5**	**RH5**	**10.5**	N	2	N	N	2	4	N	11	27	17	27	227	68	452	1315	899	281	17579	19166	4602			
**5**	**RH5**	**10.5**	N	N	N	N	4	N	N	15	25	5	8	244	76	499	1540	1628	815	25481	27505				
**5**	**RH5**	**8.5**	N	2	N	N	N	5	N	27	22	7	9	415	361	619	3234	6109							
**5**	**RH5**	**11**	N	N	N	N	N	N	N	N	6	N	2	86	22	69	489	638	189	7802	9312	4051			
**5**	**RH5**	**12**	N	N	N	2	N	N	N	3	5	4	N	130	127	74	528	1577	661	6519	17921	8875	5389	177190	150156
**5**	**RH5**	**12**	N	N	N	N	N	N	N	N	5	N	N	120	50	44	656	477	141	8501	10346	1283	5424	82977	
**5**	**RH5**	**10.5**	N	N	N	N	2	N	N	27	14	2	3	129	73	268	969	943	290	15990	16323	4844			
**5**	**RH5**	**9**	N	N	N	N	2	2	N	36	52	22	44	854	378	753	7447	7875	3036						
**5**	**RH5**	**10**	N	N	3	N	3	N	N	14	41	20	25	251	179	402	1404	2117	1278	25476	35053				
**5**	**RH5**	**10.5**	N	N	N	N	N	N	N	8	6	7	2	149	38	51	700	543	146	8147	10925	1375			
**5**	**RH5**	**10.5**	N	N	N	N	1	2	N	10	24	1	10	136	56	185	696	751	405	8083	10189				
**5**	**RH5**	**10.5**	N	N	N	N	1	N	3	21	29	3	13	103	54	145	720	653	311	8259	8855	3717			
**5**	**RH5**	**10.5**	N	N	N	N	N	3	2	6	19	10	2	128	84	54	765	703	175	9292	16139	4066			
**5**	**RH5**	**11.5**	N	N	N	N	N	N	N	8	23	4	9	365	132	186	1313	2464	1081	27585	33052	14668	65187	346431	
**6**	**Control**	**8.5**	N	N	N	N	N	N	N	25	37	6	14	479	81	901	3030	2357							
**6**	**Control**	**11**	N	N	N	N	5	N	3	14	23	3	16	297	117	532	1634	1358	364	27742	33942	9921	90762		
**6**	**Control**	**9**	N	N	N	2	1	5	N	7	13	N	4	128	78	197	631	815	394						
**6**	**Control**	**8.5**	N	N	N	N	3	N	2	45	131	33	134	2313	1667	3965	23178	33738							
**6**	**Control**	**9.5**	N	N	N	N	N	N	N	1	25	3	9	160	112	90	846	2114	1375	15514					
**6**	**Control**	**9.5**	N	N	N	N	N	N	4	16	29	19	17	624	628	496	4937	6944	2316	56968					
**6**	**Control**	**9.5**	N	N	3	N	N	5	N	15	50	21	38	607	332	907	5815	7108	2590	87891					
**6**	**Control**	**10**	N	N	N	N	N	N	2	20	14	13	42	572	272	872	5583	5205	3375	124063	192971				
**6**	**Control**	**10**	N	N	N	N	N	N	3	18	54	8	33	513	196	758	3113	3544	1288	49512	73286				
**6**	**Control**	**8.5**	N	N	N	N	N	4	N	3	10	N	44	252	193	526	2193	1855							
**6**	**Control**	**8**	N	N	N	N	4	N	4	96	114	28	142	2691	2558	3636									
**6**	**Control**	**10**	N	N	N	N	N	N	1	23	17	7	35	533	410	650	5174	7710	2667	114317	189987				
**6**	**Control**	**10.5**	N	N	N	N	N	N	N	5	8	5	1	104	87	11	198	606	170	692	3972	2021			
**6**	**Control**	**12**	N	N	N	N	N	N	N	1	N	N	N	10	19	5	79	114	40	3118	1557	432	2957	20706	6314
**6**	**Control**	**9**	N	N	N	N	N	10	3	26	29	22	19	630	382	983	4455	7680	2986						

Data S1C: Raw qPCR data (parasites/mL) for VAC063A.

Top row represents day of follow-up visit post blood-stage CHMI. Data highlighted in red represent qPCR measurement at time-point of diagnosis according to protocol, also referred to as "day of diagnosis" (DoD) for a particular individual. N = PCR negative for all three triplicate readings in the assay. Squares highlighted in grey indicate negative or <20 p/mL below minimum positive reporting criteria. Squares highlighted in blue indicate a sample taken immediately pre-treatment at the next clinic visit following diagnosis; where this occurs, volunteers were officially diagnosed based on thick-film microscopy and/or qPCR data obtained in real time between clinic visits.
