# Supplementary material for: Reduced blood-stage malaria growth and immune correlates in humans following RH5 vaccination
Source: Med. 2021 Jun 11;2(6):701–719.e19. doi: 10.1016/j.medj.2021.03.014 (PMC8240500; doi:10.1016/j.medj.2021.03.014)
Supplement: Data S1 — . Vaccine safety and qPCR data, related to Figures 1 and 3 (A) Laboratory AEs in vaccinated volunteers, Groups 1-5 and 7, considered possibly, probably or definitely related to vaccination. (B) Unsolicited AEs considered possibly, probably or definitely related to vaccination. As well as the list of solicited AEs collected at each visit, participants were also asked to report any other AEs that occurred, and these were recorded and assigned a MedDRA code. AEs occurring ≤ 28 days after each vaccination are shown, with maximum severity reported. Time-point(s) = days post-vaccination. (C) Raw qPCR data (parasites/mL) for VAC063A. (D) Raw qPCR data (parasites/mL) for VAC063B. [file mmc2.zip › Data S1A-D/Data S1D.pdf]

Group number	Group	DoD	D1	D2	D2.5	D3	D3.5	D4	D4.5	D5	D5.5	D6	D6.5	D7	D7.5	D8	D8.5	D9	D9.5
7 (5)	RH5	10.5	N	N	N	N	N	N	3	23	3	11	18	206	145	388	884	1839	402
7 (5)	RH5	9	N	N	N	N	N	7	2	63	35	23	74	1380	916	1307	9836	7798	
7 (5)	RH5	10	N	2	N	N	N	N	N	16	25	15	12	448	249	307	1957	4012	1049
7 (5)	RH5	9	N	N	N	N	N	4	N	43	38	16	115	957	625	1071	8743	11900	2832
7 (5)	RH5	10	N	N	N	N	N	2	N	N	9	N	21	159	101	172	906	1649	388
7 (5)	RH5	10	N	N	N	N	N	N	N	13	19	4	14	459	335	496	2226	3320	943
7 (5)	RH5	12	N	N	N	N	N	N	N	9	N	2	6	33	20	43	176	169	49
7 (5)	RH5	12	N	N	N	N	N	N	N	1	10	7	6	118	128	65	501	1261	460
7 (5)	RH5	9	N	N	N	N	N	N	4	30	21	17	45	988	533	1115	6203	7137	
8 (6)	Re-ch Control	9	N	N	N	N	3	3	3	37	23	26	49	1039	890	1300	7730	11291	3145
8 (6)	Re-ch Control	8.5	N	N	N	N	N	4	N	104	122	27	210	2899	2349	3346	37627	40584	
8 (6)	Re-ch Control	20 (treated)	N	N	N	N	N	N	N	N	N	N	N	N	N	N	N	N	N
8 (6)	Re-ch Control	9	N	N	N	N	11	N	N	41	31	32	58	920	937	497	7605	15615	5739
8 (6)	Re-ch Control	13	N	N	N	N	N	N	N	N	2	N	N	20	N	14	47	80	23
8 (6)	Re-ch Control	10	N	N	N	N	1	N	N	27	19	24	57	707	358	945	5475	6140	1352
8 (6)	Re-ch Control	8.5	N	N	N	N	N	N	2	144	167	98	268	3839	4148	3772	22415	56741	
8 (6)	Re-ch Control	8.5	N	N	N	N	N	7	N	41	43	4	147	1077	668	2753	10306	10754	
9	Control	10	N	N	N	N	N	N	N	61	50	17	110	1171	569	2237	6877	6272	2365
9	Control	9	N	N	N	N	N	2	N	86	68	19	102	1398	743	2002	9020	10508	1717
9	Control	8	N	N	N	N	N	7	N	261	152	79	455	5521	3666	16339	81910		
9	Control	8.5	N	2	N	2	3	3	3	122	89	89	127	3958	3630	4573	25963	45327	
9	Control	8.5	N	N	N	N	N	3	2	83	95	43	128	1768	1369	3575	23363	28926	
9	Control	8.5	N	N	N	N	N	4	N	69	55	17	162	1150	797	3250	10585	11042	

Group number	Group	DoD	D10	D10.5	D11	D11.5	D12	D12.5	D13	D13.5	D14	D14.5	D15	D15.5	D16	D17	D18	D19	D20
7 (5)	RH5	10.5	9016	13303	3643														
7 (5)	RH5	9																	
7 (5)	RH5	10	24993	39290															
7 (5)	RH5	9																	
7 (5)	RH5	10	16847	20360															
7 (5)	RH5	10	23497	23911															
7 (5)	RH5	12	1460	1783	682	2379	16009	11880											
7 (5)	RH5	12	6716	6924	6016	9484	69677	59337											
7 (5)	RH5	9																	
8 (6)	Re-ch Control	9																	
8 (6)	Re-ch Control	8.5																	
8 (6)	Re-ch Control	20 (treated)	N	N	N	N	N	N	N	N	N	N	N		N	N	N	10	2
8 (6)	Re-ch Control	9																	
8 (6)	Re-ch Control	13	219	233	309	439	2624	2624	2148	11532									
8 (6)	Re-ch Control	10	97631	116963															
8 (6)	Re-ch Control	8.5																	
8 (6)	Re-ch Control	8.5																	
9	Control	10	100946	111129															
9	Control	9																	
9	Control	8																	
9	Control	8.5																	
9	Control	8.5																	
9	Control	8.5																	

Data S1D: Raw qPCR data (parasites/mL) for VAC063B.

Top row represents day of follow-up visit post blood-stage CHMI. Data highlighted in red represent qPCR measurement at time-point of diagnosis according to protocol, also referred to as "day of diagnosis" (DoD) for a particular individual. One volunteer was treated at dC+20 and is highlighted in orange. N = PCR negative for all three triplicate readings in the assay. Squares highlighted in grey indicate negative or <20 p/mL below minimum positive reporting criteria. Squares highlighted in blue indicate a sample taken immediately pre-treatment at the next clinic visit following diagnosis; where this occurs, volunteers were officially diagnosed based on qPCR data obtained in real time between clinic visits.
