# Supplementary material for: Reduced blood-stage malaria growth and immune correlates in humans following RH5 vaccination
Source: Med. 2021 Jun 11;2(6):701–719.e19. doi: 10.1016/j.medj.2021.03.014 (PMC8240500; doi:10.1016/j.medj.2021.03.014)
Supplement: Data S2 — . Modeling information, related to Figures 2 and 6 (A) Modeling of antibody kinetics. (B) R notebook (analysis_Rh5.Rmd) for the systems serology analysis. [file mmc3.zip › Data S2/Data S2A/Data S2A Modelling.pdf]

Data S2A Modelling Methods

Information on modelling of antibody kinetics following vaccination with RH5.1/AS01$_B$.

* Technical queries to michael.white@pasteur.fr

Antibody Kinetics Model *

Following vaccination with dose j, we assume that the proliferation and differentiation of B cells leads to a boost in antibody secreting cells (ASC) of size β_j following a delay of time δ. A proportion ρ of these ASCs are assumed to be short-lived with half-life d_s, with a proportion $1 - \rho$ being long-lived with half-life d_l. It is assumed that all ASC secrete IgG molecules which decay with a half-life d_a. The antibody level of an individual at time t after vaccination with dose j is given by:

$$Ab_j(t) = Ab_0 + \beta_j \left(\rho_j \frac{e^{-r_s(t-\delta)} - e^{-r_a(t-\delta)}}{r_a - r_s} + (1 - \rho_j) \frac{e^{-r_l(t-\delta)} - e^{-r_a(t-\delta)}}{r_a - r_l} \right)$$

where $r_a = \log(2)/d_a$ is the rate of decay of IgG molecules, $r_s = \log(2)/d_s$ is the rate of decay of short-lived plasma B cells, and $r_l = \log(2)/d_l$ is the rate of decay of long-lived plasma B cells. It is assumed that the measured antibody level before vaccination was Ab_0. This equation can be used to describe the antibody response generated following each dose. In particular, we assume the proportion of short-lived plasma cells is fixed at ρ, except in Group 3 following dose 3 where the proportion of short-lived plasma cells is given by ρ_{g3}. The total antibody response is obtained by summing the response generated following each dose as follows:

$$Ab(t) = Ab_1(t) + Ab_2(t) + Ab_3(t)$$

Fitting the Model to Data

The model was fitted to longitudinal antibody level measurements from all participants. Mixed effects methods were used to capture the natural variation in antibody kinetics between individual participants, whilst estimating the average value and variance of the immune parameters across the entire population of individuals. The models were fitted in a Bayesian framework using Markov Chain Monte Carlo (MCMC) methods. Mixed effects methods allow individual-level parameters to be estimated for each participant separately, with these individual-level (or mixed effects) parameters being drawn from global distributions. For example, for each participant *n* the half-life of the short-lived ASCs may be estimated as d_s^n (an individual-level parameter). These *N* estimates of the local parameters d_s^n will be drawn from a probability distribution. A log-Normal distribution is suitable as it has positive support on $[0, \infty)$. Thus we have $log(d_s^n) \sim N(\mu_s, \sigma_s^2)$. The mean d_s and the variance Σ_s^2 of the estimates of d_s^n are given by $d_s = e^{\mu_s + \frac{\sigma_s^2}{2}}$ and $\Sigma_s^2 = \left(e^{\sigma_s^2} - 1\right)e^{2\mu_s + \sigma_s^2}$. The relationship between the parameters describing the population-level distribution and the parameters for each individual in the population are depicted in the schematic diagram in **Modelling Fig. 1**.

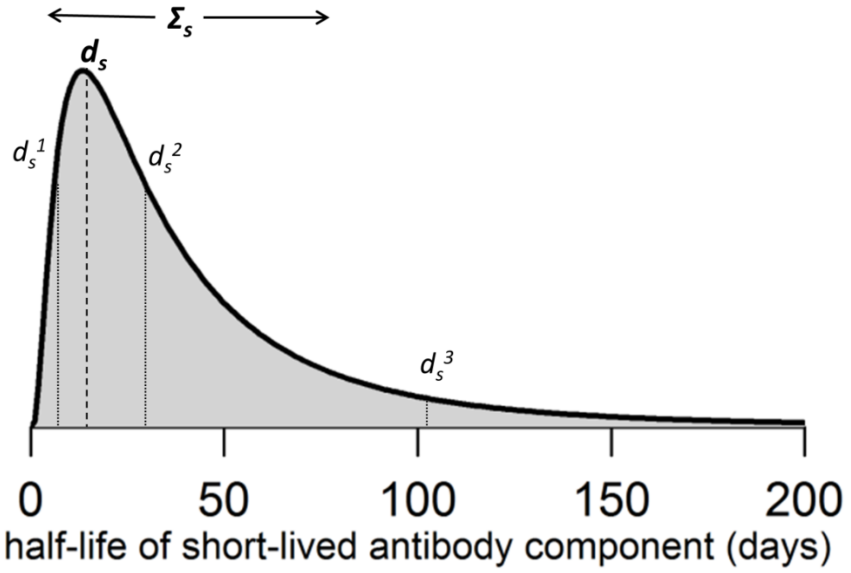

Modelling Figure 1. Schematic representation of the relationship between the population-level and individual-level parameters for the half-life of the short-lived ASC. It is assumed that half-lives are log-Normally distributed throughout the population. The population-level parameters define the mean d_s and standard deviation Σ_s of this distribution. The individual-level parameters d_s^n for each of the n participants follow the log-Normal distribution defined by the population-level parameters. Three representative individual-level parameters are shown for illustration.

Model Likelihood

For individual *n* we have data on observed antibody levels $A^n = \{a_1, \ldots, a_K\}$ at times $T^n = \{t_1, \ldots, t_K\}$. We denote $D^n = (A^n, T^n)$ to be the vector of data for individual *n*. For individual *n*, the parameters Ab_0^n, β_j^n, δ^n, d_s^n , d_l^n , d_a^n and ρ^n are estimated. For individuals in Group 3, the proportion of short-lived plasma cells following the third dose is ρ_{g3}^n. These parameters are denoted $\theta^n = \{Ab_0^n, \beta_j^n, \delta^n, d_s^n, d_l^n, d_a^n, \rho^n, \rho_{g3}^n\}$. The model predicted antibody levels will be $\{A(t_1), A(t_2), \ldots, A(t_K)\}$. We assume log-Normally distributed measurement error such that the difference between log(a_j) and log($Ab(t_j)$) is Normally distributed with variance σ_{obs}^2. For model predicted antibody levels $Ab(t_j)$ the data likelihood for individual *n* is given by:

$$L^n(\theta^n|D^n) = \prod_{k\in K} \frac{e^{-\frac{\left(log(a_j)-log\left(Ab(t_j)\right)\right)^2}{2\sigma_{obs}^2}}}{a_k\sigma_{obs}\sqrt{2\pi}}$$

Mixed Effects Likelihood

As described above, for each individual the following parameters are to be estimated: $\theta^n = \{Ab_0^n, \beta_1^n, \beta_2^n, \beta_3^n, \delta^n, d_s^n, d_l^n, d_a^n, \rho_{1,2}^n, \rho_3^n\}$. For volunteers in Group 3, the mixed effects likelihood can be written as follows:

$$L_{mix}^n(\theta^n|D^n) = \left(\frac{e^{-\frac{\left(log(Ab_0^n)-\mu_{Ab,0}\right)^2}{2\sigma_{Ab,0}^2}}}{\sqrt{2\pi}Ab_0^n\sigma_{Ab,0}}\right)\left(\frac{e^{-\frac{\left(log(\beta_1^n)-\mu_{\beta_1}\right)^2}{2\sigma_{\beta_1}^2}}}{\sqrt{2\pi}\beta_1^n\sigma_{\beta_1}}\right)\left(\frac{e^{-\frac{\left(log(\beta_2^n)-\mu_{\beta_2}\right)^2}{2\sigma_{\beta_2}^2}}}{\sqrt{2\pi}\beta_2^n\sigma_{\beta_2}}\right)\left(\frac{e^{-\frac{\left(log(\beta_3^n)-\mu_{\beta_3}\right)^2}{2\sigma_{\beta_3}^2}}}{\sqrt{2\pi}\beta_3^n\sigma_{\beta_3}}\right)$$

$$\left(\frac{e^{-\frac{\left(log(d_s^n)-\mu_{d,s}\right)^2}{2\sigma_{d,s}^2}}}{\sqrt{2\pi}d_s^n\sigma_{d,s}}\right)\left(\frac{e^{-\frac{\left(log(d_l^n)-\mu_{d,l}\right)^2}{2\sigma_{d,l}^2}}}{\sqrt{2\pi}d_l^n\sigma_{d,l}}\right)\left(\frac{e^{-\frac{\left(log(d_a^n)-\mu_{d,a}\right)^2}{2\sigma_{d,a}^2}}}{\sqrt{2\pi}d_a^n\sigma_{d,a}}\right)\left(\frac{e^{-\frac{\left(log\left(\frac{\rho_{1,2}^n}{1-\rho_{1,2}^n}\right)-\mu_{\rho_{1,2}}\right)^2}{2\sigma_{\rho_{1,2}}^2}}}{\sqrt{2\pi}\rho_{1,2}^n(1-\rho_{1,2}^n)\sigma_{\rho_{1,2}}}\right)\left(\frac{e^{-\frac{\left(log\left(\frac{\rho_3^n}{1-\rho_3^n}\right)-\mu_{\rho_3}\right)^2}{2\sigma_{\rho_3}^2}}}{\sqrt{2\pi}\rho_3^n(1-\rho_3^n)\sigma_{\rho_3}}\right)$$

As the proportion of the ASCs that are long-lived must be bounded by 0 and 1, the individual-level parameters $\rho_{1,2}^n$ and ρ_3^n are assumed to be drawn from logit-Normal distributions. Note that the pre-existing antibody level Ab_0^n will be variable, depending on a large number of covariates such as age and past exposure. We therefore do not attempt to constrain pre-existing antibody levels using mixed effects.

Total Model Likelihood

Denote $D = \{D^1, \dots, D^N\}$ to be the vector of data for all N volunteers. We denote θ to be the combined vector of population-level parameters and individual-level parameters to be estimated. The total likelihood is obtained by multiplying the likelihood for each volunteer

$$L_{total}(\theta|D) = \prod_{n\epsilon N} L_{mix}^{n}(\theta^{n}|D^{n}) L_{mod}^{n}(\theta^{n}|D^{n})$$

Markov Chain Monte Carlo parameter update

The model was fitted to the data using Markov Chain Monte Carlo (MCMC) methods using software for Bayesian statistical inference of non-linear mixed-effects models. This utilises a Metropolis-within-Gibbs sampler, whereby population-level parameters are updated using a Gibbs sampler, and individual-level and observational parameters are updated using a Metropolis-Hastings sampler.

Description	Prior estimate	Posterior estimate
Group 1		
boost dose 1 (day 0): 2µg RH5.1 / 0.5mL AS01	7.07 (1.04, 48.09)	1.06 (0.29, 31.73)
boost dose 2 (day 28): 2µg RH5.1 / 0.5mL AS01	7.07 (1.04, 48.09)	2.73 (1.77, 4.80)
boost dose 3 (day 56): 2µg RH5.1 / 0.5mL AS01	7.07 (1.04, 48.09)	5.17 (2.45, 11.27)
proportion of short-lived ASCs (all doses)	93.3% (79.1%, 98.0%)	93.2% (86.5%, 96.5%)
Group 2		
boost dose 1 (day 0): 10µg RH5.1 / 0.5mL AS01	7.07 (1.04, 48.09)	0.72 (0.35, 2.82)
boost dose 2 (day 28): 10µg RH5.1 / 0.5mL AS01	7.07 (1.04, 48.09)	6.03 (3.83, 10.42)
boost dose 3 (day 56): 10µg RH5.1 / 0.5mL AS01	7.07 (1.04, 48.09)	2.79 (0.89, 7.61)
proportion short-lived ASCs (all doses)	93.3% (79.1%, 98.0%)	95.9% (92.1%, 97.8%)
Groups 3 & 4		
boost dose 1 (day 0): 50µg RH5.1 / 0.5mL AS01	7.07 (1.04, 48.09)	0.96 (0.40, 4.63)
boost dose 2 (day 28): 50µg RH5.1 / 0.5mL AS01	7.07 (1.04, 48.09)	8.97 (5.73, 16.52)
Group 3		
boost dose 3 (day 182): 50µg RH5.1 / 0.5mL AS01	7.07 (1.04, 48.09)	20.62 (12.63, 37.23)
proportion of short-lived ASCs (doses 1 & 2)	93.3% (79.1%, 98.0%)	94.2% (84.9%, 97.3%)
proportion of short-lived ASCs (dose 3)	93.3% (79.1%, 98.0%)	87.7% (77.1%, 93.5%)
Group 4		
boost dose 3 (day 56): 50µg RH5.1 / 0.5mL AS01	7.07 (1.04, 48.09)	2.35 (0.69, 7.37)
proportion short-lived ASCs (all doses)	93.3% (79.1%, 98.0%)	95.4% (90.7%, 97.7%)
All groups		
delay in boosting of antibody responses (days)	3.55 (1.03, 12.19)	7.90 (6.94, 9.14)
half-life of short-lived ASCs (days)	8.84 (3.06, 26.18)	17.03 (11.98, 24.52)
half-life of long-lived ASCs (days)	707.8 (28.4, 17560.9)	2203.4 (758.8, 25086.1)
half-life of IgG molecules (days)	20.43 (15.20, 27.46)	19.00 (14.73, 24.16)

Modelling Table 1: Parameter estimates.

Parameters of the antibody kinetics model are presented as posterior medians with 95% credible intervals. The model is fitted in a mixed-effects framework, so for every parameter we estimate the distribution within the entire population rather than a fixed value. We present the mean of the estimated distributions.

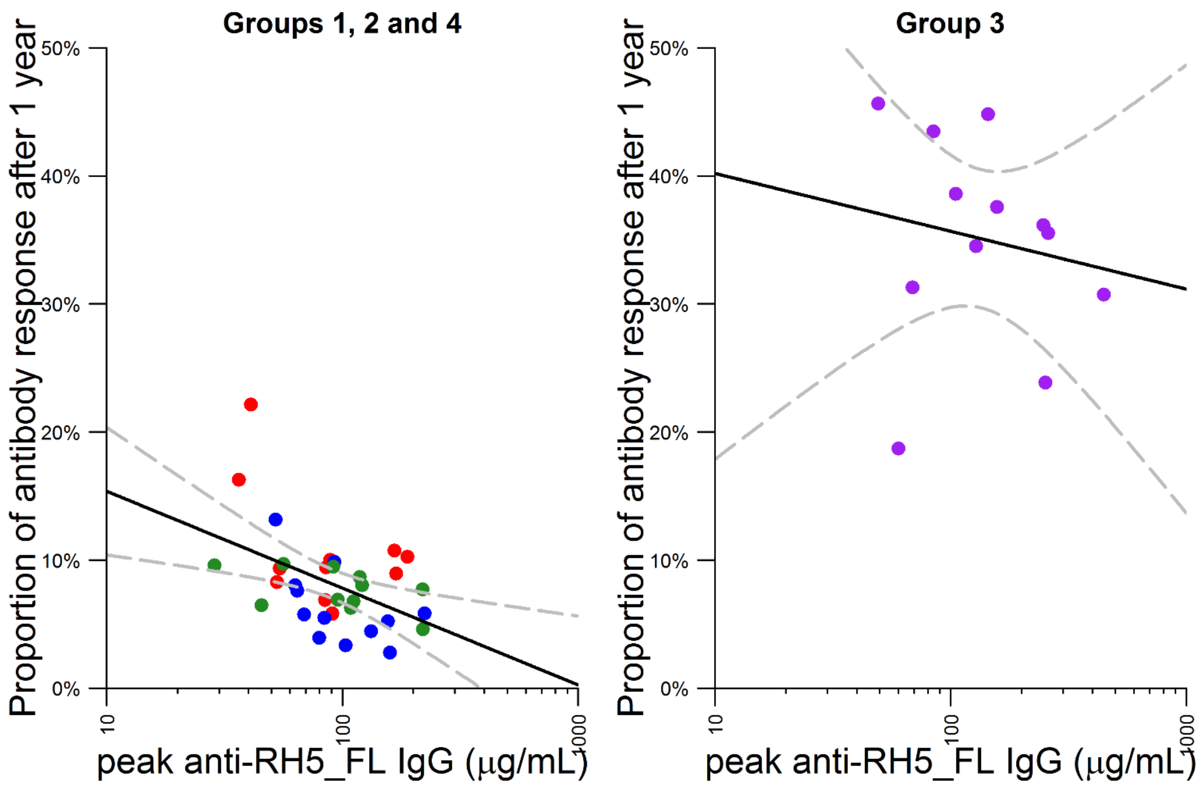

Modelling Figure 1: Association peak immunogenicity and longevity of antibody response.

(**Left**) In Groups 1, 2 and 4, the peak antibody response and longevity (measured as the proportion of response remaining at 1 year) are negatively associated ($P = 0.0045$). (**Right**) In Group 3, there is no statistically significant association between peak antibody response and longevity ($P = 0.61$).

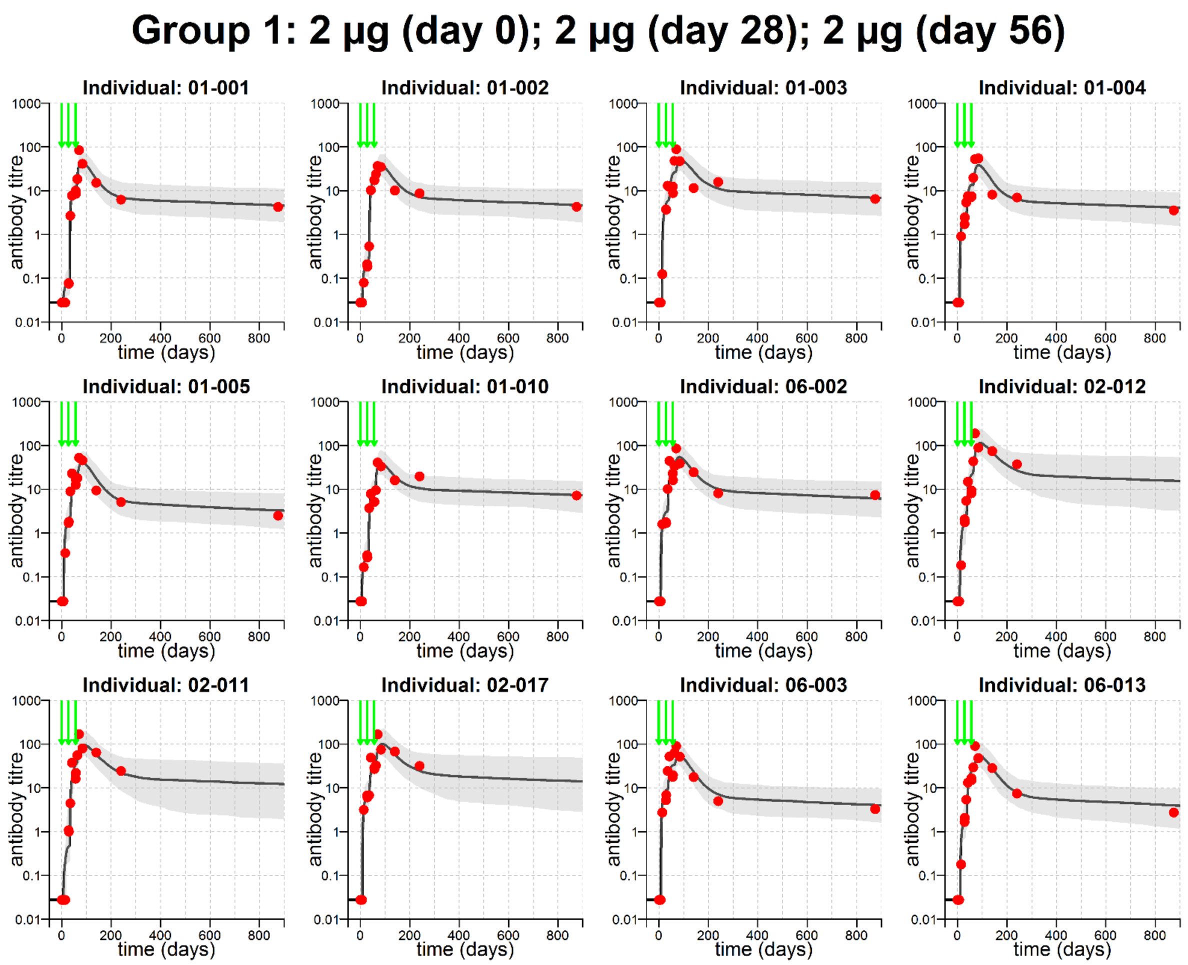

Modelling Figure 2: Fits of antibody kinetic model to Group 1.

Red points represent measured data. Green arrows represent the timing of vaccine doses. Black lines represent posterior median model prediction. Grey shaded region represents the 95% credible interval.

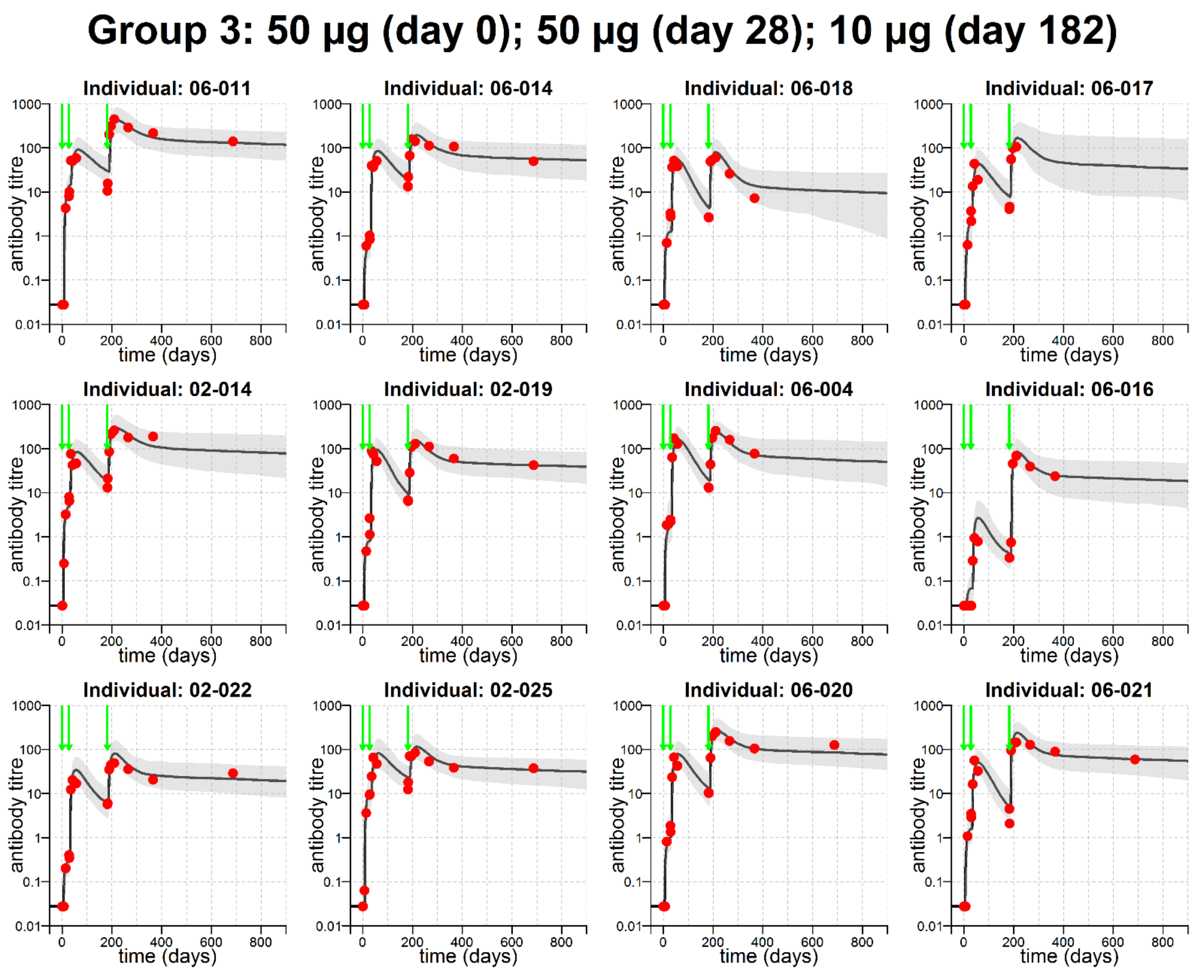

Modelling Figure 3: Fits of antibody kinetic model to Group 3.

Red points represent measured data. Green arrows represent the timing of vaccine doses. Black lines represent posterior median model prediction. Grey shaded region represents the 95% credible interval.

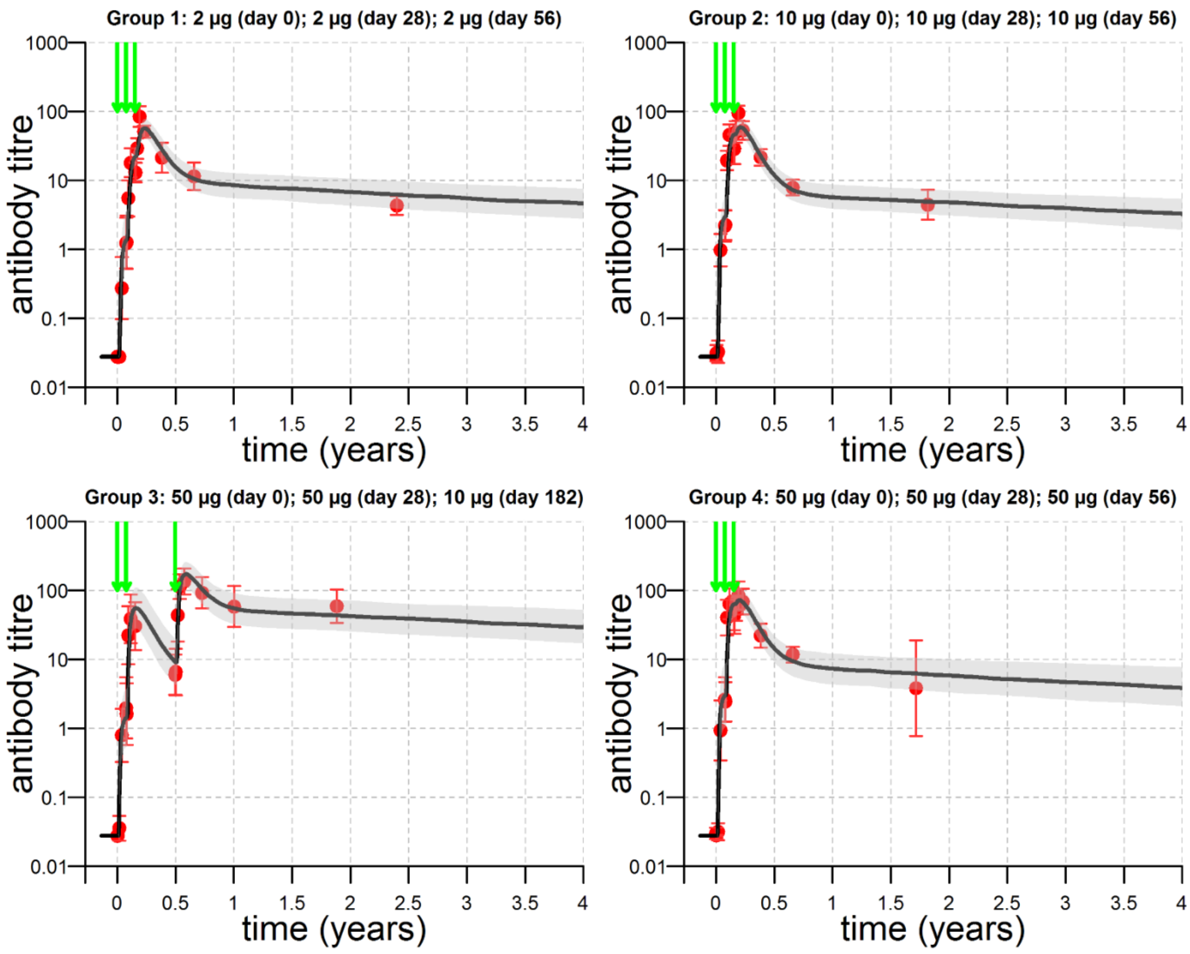

Modelling Figure 4: Comparison of antibody kinetic model fits between Groups.

Red points represent the geometric mean antibody level for all participants in a Group at a given time-point. Red vertical bars denote 95% confidence intervals. Green arrows represent the timing of vaccine doses. Black lines represent posterior median model prediction. Grey shaded region represents the 95% credible interval.
